# Diastereoselective Reformatsky Reaction Mediated by Dichlorocyclopentadienyltitanium(III)

**DOI:** 10.3390/molecules30193893

**Published:** 2025-09-26

**Authors:** Josefa L. López-Martínez, Irene Torres-García, Manuel Muñoz-Dorado, Miriam Álvarez-Corral, Ignacio Rodríguez-García

**Affiliations:** Organic Chemistry, University of Almería, CIAIMBITAL, 04120 Almería, Spain; pepaloma91@hotmail.com (J.L.L.-M.); mdorado@ual.es (M.M.-D.)

**Keywords:** Reformatsky reaction, CpTiCl_2_, diastereoselectivity, β-hydroxyesters, cross-coupling reaction

## Abstract

The Reformatsky reaction, first reported in 1887, has long been recognized as a fundamental method for carbon–carbon bond construction due to its mild conditions and functional group tolerance. Over the past few decades, this transformation has undergone a notable revival, with modern catalytic variants addressing limitations of stoichiometric protocols and expanding its role in complex molecule synthesis. Yet, despite its versatility, achieving stereoselective control remains a longstanding challenge. Herein we report the use of dichlorocyclopentadienyltitanium(III) (CpTiCl_2_), generated in situ from CpTiCl_3_ and manganese, as an efficient catalyst for Reformatsky-type couplings of aldehydes with α-haloesters and α-iodonitriles. Under mild conditions, CpTiCl_2_ promotes the formation of β-hydroxy esters in high yields and with significant diastereoselective preference for the *syn* isomer (up to 100:0 *syn:anti*). This behavior contrasts sharply with the poor or *anti*-selective outcomes previously observed with titanocene(III) chloride (Cp_2_TiCl). Mechanistic analysis suggests that the unique steric and electronic environment of CpTiCl_2_—characterized by enhanced Lewis acidity and increased coordination vacancies—favors a Zimmerman–Traxler-type transition state that enforces *syn* stereocontrol. The methodology tolerates a wide variety of substrates, including aliphatic and aromatic aldehydes as well as α-iodonitriles, extending the scope of titanium-mediated Reformatsky chemistry. These findings establish CpTiCl_2_ as a sustainable, selective, and robust organotitanium catalyst for stereoselective carbon–carbon bond formation, providing a promising alternative to the Nugent reagent and paving the way for new applications in complex molecule synthesis.

## 1. Introduction

The Reformatsky reaction, discovered in 1887 by Serhii Mykolayovych Reformatskyi, stands as one of the most enduring and adaptable transformations in organic chemistry [1]. By introducing a zinc-mediated coupling of ethyl haloacetates with aldehydes or ketones to yield β-hydroxyesters, Reformatsky offered a method of carbon–carbon bond formation that was both milder and more selective than the strongly basic organometallic reagents available at the time [2]. Unlike early nucleophiles, zinc enolates tolerated functional groups and enabled access to sterically hindered products, giving the reaction a distinct role in synthetic methodology. This complementarity to the later-emerging Grignard chemistry ensured its longevity, and more than a century later its core principle —the in situ generation of a metal enolate from an α-halo carbonyl compound, followed by nucleophilic addition to a carbonyl electrophile—continues to inspire innovation [3].

Over the past three decades, the Reformatsky reaction has experienced a revival [4,5]. Classical stoichiometric protocols, though effective, were hampered by metal waste, purification issues, and limited substrate scope. Modern developments have reimagined the process as a versatile catalytic platform [6,7] capable of operating under milder and more sustainable conditions. This shift has redefined the meaning of the “Reformatsky reaction” now understood as a family of transformations in which α-halo carbonyls engage electrophiles in the presence of a wide range of metals. Transition metals such as chromium [8], iridium [9,10], or palladium [11] have been harnessed to tackle challenges of selectivity, reactivity, and sustainability, demonstrating how a traditional transformation can be redefined by contemporary design. Other metals have also attracted considerable attention for their ability to mediate the coupling between α-haloesters and carbonyl compounds while minimizing competing reactions and byproduct formation, like indium [12], germanium [13,14] or tin [15,16]. The modern Reformatsky reaction has become a versatile tool in complex molecule construction, demonstrating advances in stereocontrol and efficiency [17]. Once limited to simple β-hydroxyesters, it now provides powerful strategies for assembling intricate architectures and enantioenriched building blocks, securing its place in contemporary natural product and pharmaceutical synthesis [8,10,18,19,20].

Among these modern advances, titanium(III) reagents hold a particularly prominent place. Titanocene(III) chloride (Cp_2_TiCl), known as the Nugent–RajanBabu reagent, has proven to be a versatile tool in organic synthesis [21]. Functioning as a powerful single-electron transfer (SET) agent, it promotes a wide variety of radical and organometallic transformations under mild conditions. In addition to its efficiency, Cp_2_TiCl is often considered environmentally friendly, as it can be regenerated from the widely available Cp_2_TiCl_2_, aligns with the principles of Green Chemistry, and operates without the need for stoichiometric additives [22,23]. These features, combined with its low toxicity and operational simplicity, have contributed to its prominent position in contemporary synthetic methodology, which spans multiple domains of carbon–carbon bond formation [24]. Its applications range from umpolung processes [25,26], to radical-mediated ring-opening and metallation reactions [27], cooperative catalysis [28,29], and even hydrogen-atom transfer and photochemical water splitting [21,30].

**Scheme 1:**
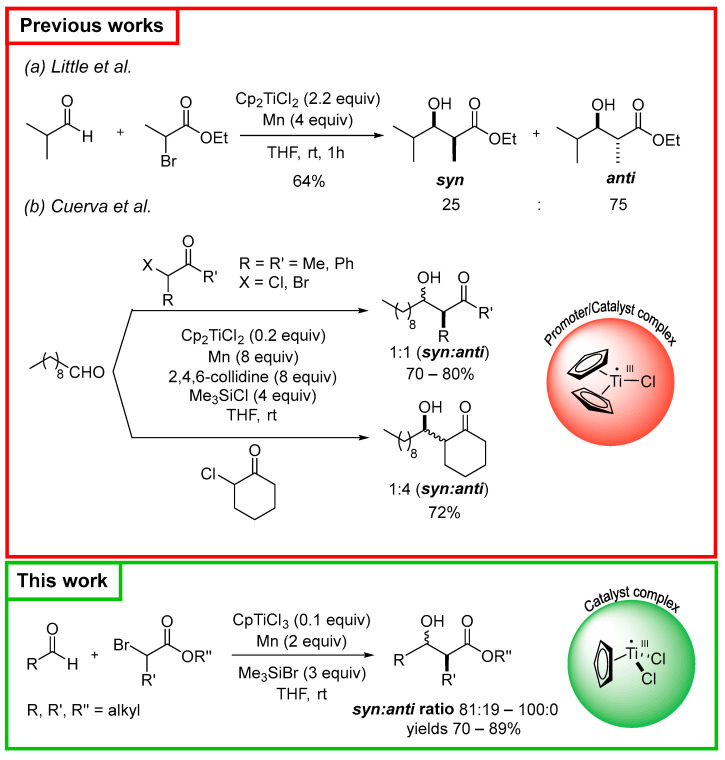
Previous ((**a**): [31] and (**b**): [33]) and current works on diastereoselective versions of the Reformatsky reaction mediated and/or promoted by titanium(III).

The utility of Cp_2_TiCl also extends to Reformatsky-type reactions [31,32,33]. Compared to zinc-based protocols, the titanium(III)-catalyzed variant proceeds under milder, homogeneous conditions and typically affords shorter reaction times and cleaner product mixtures. Early work by Little and co-workers in 2003 demonstrated the feasibility of these transformations with α-haloesters and aldehydes, although stoichiometric amounts of titanocene were required (Scheme 1a) [31]. Later, catalytic versions were developed [32], being specially successful the use of the combination of TMSCl and collidine as titanocene regenerating system introduced by Cuerva and co-workers (Scheme 1b) [33].

However, despite these advantages, the diastereoselective outcome remains a critical limitation: most reported cases deliver poor *syn*/*anti* ratios or are even biased toward the *anti* isomer, severely restricting the applicability of this otherwise valuable methodology [31,32,33] (Scheme 1).

To address this challenge, we turned our attention to the closely related half-sandwich complex CpTiCl_2_ [34]. Unlike Cp_2_TiCl, this mono-cyclopentadienyl species possesses an 11-electron coordination sphere (vs. 15 for Cp_2_TiCl), providing enhanced Lewis acidity, greater coordination flexibility, and reduced steric hindrance. These features are expected to exert a decisive influence on both reactivity and stereocontrol. Indeed, CpTiCl_2_, generated in situ by the reduction of CpTiCl_3_ with Mn, has already demonstrated excellent performance in Barbier-type allylation and propargylation of aldehydes with high regio- and stereoselectivity [35,36]. Building on this precedent, we envisaged that CpTiCl_2_ could overcome the long-standing stereoselectivity issues in titanium(III)-mediated Reformatsky reactions. Herein we report the first systematic study of CpTiCl_2_ in Reformatsky-type couplings, demonstrating that this reagent not only matches the reactivity of Cp_2_TiCl but also delivers a remarkable diastereoselective preference for the syn isomer. This work highlights how subtle changes in the ligand environment and electron count of titanium complexes can translate into substantial improvements in stereochemical control, thereby expanding the synthetic scope and significance of Reformatsky chemistry (Scheme 1).

## 2. Results and Discussion

**Scheme 2:**
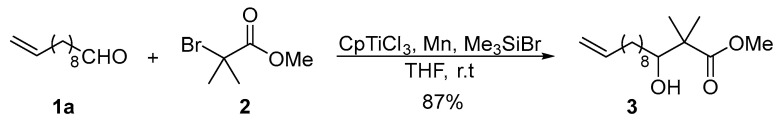
Reformatsky reaction catalyzed by CpTiCl_2_.

To evaluate the performance of CpTiCl_2_ in Reformatsky-type couplings, we first examined the benchmark reaction between undec-10-enal (**1a**) and methyl 2-bromo-2-methylpropanoate (**2**) (Scheme 2) under catalytic conditions similar to those previously developed for Barbier-type processes in our group [35,36]. CpTiCl_2_ is obtained by reduction with Mn of CpTiCl_3_, and as a regenerating agent we used only Me_3_SiBr instead of the regenerating mixture of Me_3_SiBr and Et_3_N·HBr used in previous works [35]. The desired β-hydroxy ester **3** was obtained in excellent yield (87%, Scheme 2).

The overall efficacy of the catalytic method was evaluated with a variety of aldehydes (**1a**–**1d**) and α-halo esters (**4a**–**4c**), obtaining a collection of β-hydroxy esters with good to high yields (70–89% Table 1).

The aromatic aldehyde **1d** (Table 1, entry 8) required stoichiometric conditions to achieve acceptable conversion, as the reaction was sluggish under catalytic conditions. This outcome aligns with previous observations that such substrates are prone to undergo pinacol coupling in the presence of Cp_2_TiCl [35,37]. However, under stoichiometric conditions with CpTiCl_2_ no pinacol-type by products were detected, and the desired β-hydroxy ester was obtained in notable yield (70%) with outstanding diastereoselectivity (100% *syn*).

The key feature of this transformation is its diastereoselective outcome. Across a wide substrate range, the syn isomer predominated in ratios of approximately 8:2. Even more strikingly, reactions involving the bulky *tert*-butyl ester **4c** (Table 1, entries 6–8) yielded exclusively the *syn* isomer (100:0), underscoring the powerful stereocontrol achievable with CpTiCl_2_. This stands in sharp contrast to Cp_2_TiCl-mediated Reformatsky reactions, where either poor selectivity or anti-bias is generally reported [31,32,33].

**Scheme 3:**
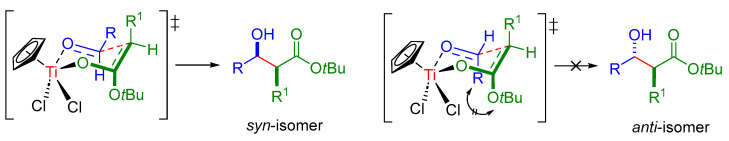
“Zimmerman–Traxler”-like transition state.

We propose that the observed stereochemical bias originates from a Zimmerman–Traxler-type transition state (Scheme 3). In this chair-like six-membered arrangement, the ester substituent occupies a pseudo-equatorial position, thereby minimizing steric repulsions with the aldehyde substituent. The result is a predictable *syn* orientation of the new stereocenters. The ability of CpTiCl_2_ to enforce this ordered geometry likely arises from its enhanced Lewis acidity, reduced steric hindrance, and greater coordination flexibility compared to Cp_2_TiCl. These features facilitate strong coordination to both the ester and the aldehyde, favoring a cyclic transition state and rationalizing the exceptional *syn*-selectivity observed. In contrast, other titanium(III) systems reported previously probably operate through less ordered, non-cyclic pathways, which could account for their limited or even *anti*-selective diastereocontrol.

**Scheme 4:**
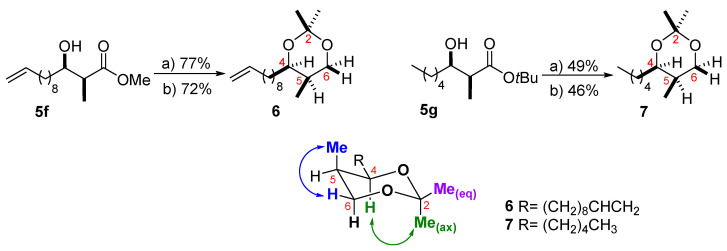
Synthesis of acetal derivatives and diagnostic NOES of **6** and **7**. (a) LiAlH_4_ (4 equiv), THF, 0 °C, 7 hr. (b) 2,2-dimethoxypropane (3 equiv), camphor sulfonic acid (0.3 equiv), DCM, 0 °C, overnight.

To elucidate the stereochemistry of the hydroxyesters, the acetals **6** and **7** were prepared in two steps starting from **5f** and **5g** (Scheme 4). First, the reduction with lithium aluminum hydride in THF at 0 °C allows the corresponding diol, and then, the acetal formation with 2,2-dimethoxypropane and camphorsulfonic acid in DCM is achieved.

**Figure 1:**
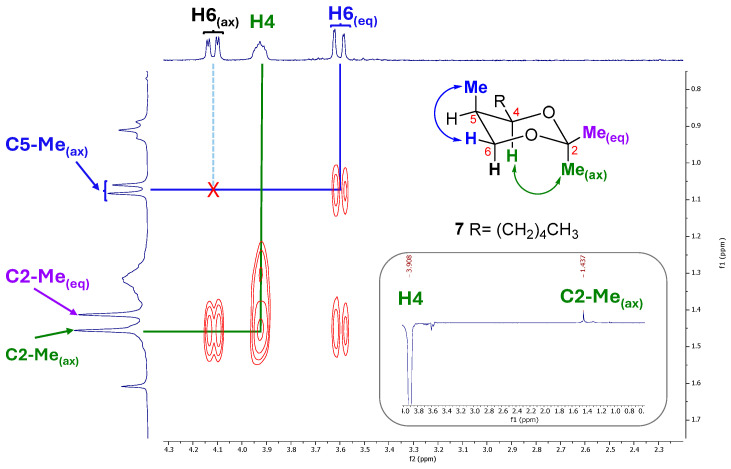
Expansions of mono and bidimensional NOE spectra of **7**.

Once the signals of acetals **6** and **7** were assigned using bidimensional NMR experiments such as HSQC and HMBC, the mono and bidimensional NOE spectra (Figure 1) enabled the identification of correlations between H4 and the axial methyl at position 2 (Scheme 4, green arrow), and the equatorial proton H6 with the methyl at position 5 (Scheme 4, blue arrow). The absence of correlation between the methyl at C5 and the axial proton H6 (Figure 1) also supports the *syn* configuration of the Reformatsky products **5f** and **5g**.

**Scheme 5:**
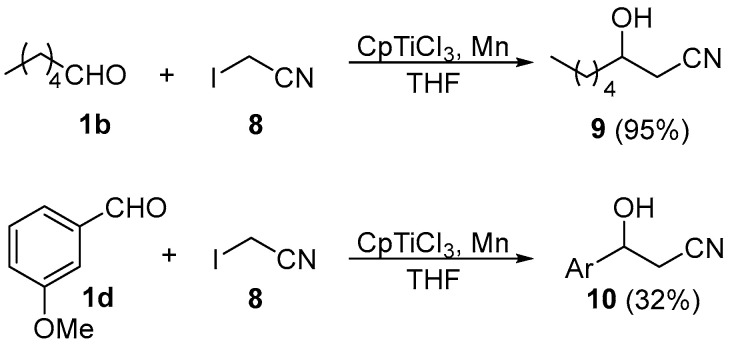
Reformatsky reaction with α-iodonitriles mediated by CpTiCl_2_.

We have also extended the scope of the reaction to α-halo nitriles (Scheme 5). CpTiCl_2_ promoted the Reformatsky reaction between iodoacetonitrile (**8**) and two electronically different aldehydes, one aliphatic and the other aromatic. The reaction with the aliphatic aldehyde **1b** proceeded efficiently, affording the β-hydroxy nitrile **9** in good yield. However, in the case of the aromatic aldehyde **1d**, pinacol coupling partially competed with the desired Reformatsky reaction, reflecting substrate-dependent limitations [35].

Taken together, these results establish CpTiCl_2_ as a robust, selective, and general catalyst for titanium-mediated Reformatsky reactions. The system overcomes the long-standing issue of poor or anti-selective diastereocontrol associated with Cp_2_TiCl, delivering instead a strong and predictable syn bias. This enhanced performance highlights the importance of subtle changes in ligand environment and electron count in dictating the outcome of organotitanium catalysis. From a broader perspective, the predictable syn-selectivity has significant synthetic implications. β-Hydroxy esters and nitriles are versatile building blocks in polyketide and macrolide synthesis, and the ability to access them stereocontrolled under mild, catalytic conditions positions CpTiCl_2_ as a valuable addition to the toolbox of sustainable C–C bond-forming methodologies.

## 3. Materials and Methods

### 3.1. General Remarks

In all experiments involving Ti(III), reactions were performed under an argon atmosphere, using oven-dried glassware in all cases. THF was distilled from Na/benzophenone under argon and was deoxygenated prior to use. NMR spectra were acquired at ambient temperature on Bruker Nanobay Avance III HD 300 MHz, Avance III HD 500 MHz, and Avance III HD 600 MHz spectrometers (Bruker Corporation, Billerica, MA, USA). ^1^H NMR, proton-decoupled ^13^C NMR and DEPT-135 were recorded in all cases. In some instances, quantitative ^13^C NMR was used for the determination of the ratio of *syn*:*anti* isomers. When required, edited HSQC was used for signal assignation and disambiguation and NOESY-1D/2D for relative configuration determination. Chemical shifts (δ) are expressed in ppm and coupling constants (*J*) in hertz (Hz). Chemical shifts are reported using CDCl_3_ as internal reference. IR Spectra were recorded on a Bruker Alpha spectrometer (Bruker Corporation, Billerica, MA, USA). Mass spectra were obtained on a Waters Xevo LC-QTof-MS instruments (Waters Corporation, Milford, MA, USA) by electrospray ionization (ESI). Reactions were monitored by thin-layer chromatography (TLC) carried out on 0.2 mm DC-Fertigfolien Alugram^®^ (Southend-On-Sea, UK) XtraSil G/UV254 silica gel plates. The TLC plates were visualized with UV light and 7% phosphomolybdic acid/heat. Flash chromatography was performed on silica gel 60 (0.04–0.06 mm) using different eluents depending on the compound to be separated. Commercially available reagents were purchased from Aldrich Chemical Co. (Saint Louis, MO, USA), Acros (Fukuoka, Japan), Alfa Aesar (Karlsruhe, Germany), and TCI (Zwijndrecht, Belgium) and used as received.

### 3.2. General Procedure A for Ti-Catalyzed Reformatsky Reaction

Under an Ar atmosphere, dry THF (8 mL/1.4 mmol of aldehyde) previously deoxygenated was added to a miscellany of CpTiCl_3_ (0.1 equiv) and Mn dust (2 equiv) resulting in a dark blue suspension. Then, Me_3_SiBr (3 equiv) was added and the mixture turned turquoise. A solution of aldehyde or ketone (1 equiv) and α-halo ester (2 equiv) in THF (2 mL/1.4 mmol of aldehyde) was added dropwise, and the reaction was stirred overnight. The mixture was filtered, diluted with EtOAc, washed with HCl 3% and brine, and dried (anhydrous MgSO_4_), and the solvent was removed. Products were purified by silica gel flash column chromatography (hexane/EtOAc mixtures).

*Methyl 3-hydroxy-2,2-dimethyltridec-12-enoate* (**3**). Reaction of undec-10-enal (**1a**) (0.24 mL, 1.19 mmol) and methyl 2-bromo-2-methylpropanoate (**2**) (0.31 mL, 2.38 mmol), according to the general procedure A, afforded product **3** (279 mg, 87%) isolated as light-yellow oil (hexane/EtOAc, 8:2; R*f:* 0.33). IR (film) *v* (cm^−1^) 3513, 2925, 2854, 1728, 1641, 1463, 1440, 1391, 1264, 1194, 1138, 1077, 992, 910, 862, 771. ^1^H NMR (300 MHz, CDCl_3_) δ (ppm) 5.90–5.77 (m, 1H), 5.04–4.93 (m, 2H), 3.72 (s, 3H), 3.67 (s, 1H), 3.62 (d, *J =* 9.7 Hz, 1H), 2.09–2.02 (m, 2H), 1.61–1.58 (m, 1H), 1.41–1.28 (m, 13H), 1.21 (s, 3H), 1.19 (s, 3H).^13^C NMR (75 MHz, CDCl_3_, DEPT) δ (ppm) 178.2 (C), 139.2 (CH), 114.1 (CH_2_), 76.7 (CH), 51.9 (CH_3_), 47.2 (C), 33.8 (CH_2_), 31.7 (CH_2_), 29.6 (CH_2_), 29.4 (CH_2_), 29.1 (CH_2_), 28.9 (CH_2_), 26.7 (CH_2_), 22.2 (CH_3_), 20.4 (CH_3_). HRMS (ESI/Q-TOF) *m*/*z*: [M+H]^+^ calcd for C_16_H_31_O_3_ 271.2273; found: 271.2258.

*Methyl 3-hydroxy-2-methyltridec-12-enoate* (**5a**). Reaction of undec-10-enal (**1a**) (0.12 mL, 0.60 mmol) and methyl 2-bromopropanoate (**4a**) (0.27 mL, 2.38 mmol), according to the general procedure A, afforded product **5a** (137 mg, 89%) as a light-yellow oil (hexane/EtOAc, 8:2; R*f:* 0.30) and an inseparable mixture of isomers (82:18 *syn:anti*). IR (film) *v* (cm^−1^) 3471, 2925, 2854, 1736, 1720, 1640, 1459, 1436, 1256, 1197, 1170, 1043, 992, 909. ^1^H NMR (600 MHz, CDCl_3_) δ (ppm) common signals: 5.82 (ddt, *J* = 17.4, 10.2, 7.2 Hz, 1H), 5.02–4.98 (m, 1H), 4.95–4.92 (m, 1H), 3.72 (s, 3H), 2.57–2.52 (m, 1H), 2.06–2.03 (m, 2H), 1.52–1.45 (m, 2H), 1.41–1.36 (m, 3H), 1.29 (br s, 9H); characteristic signals of **5a *syn***: 3.89 (dt, *J* = 8.4, 4.2 Hz, 1H), 1.19 (d, *J* = 7.2 Hz, 3H); characteristic signals of **5a *anti***: 3.67–3.65 (m, 1H), 1.22 (d, *J* = 7.2 Hz, 3H). ^13^C NMR (150 MHz, CDCl_3_, DEPT) δ (ppm) common signals: 139.2 (CH), 114.1 (CH_2_), 33.8 (CH_2_), 29.5 (CH_2_), 29.5 (CH_2_), 29.4 (CH_2_), 29.1 (CH_2_), 28.9 (CH_2_); signals of **5a *syn***: 176.6 (C), 71.8 (CH), 51.8 (CH_3_), 44.2 (CH), 33.8 (CH_2_), 26.0 (CH_2_), 10.6 (CH_3_); signals of **5a *anti***: 176.5 (C), 73.4 (CH), 51.7 (CH_3_), 45.2 (CH), 34.7 (CH_2_), 25.5 (CH_2_), 14.3 (CH_3_).

*Methyl 3-hydroxy-2-methyloctanoate* (**5b**). Reaction of hexanal (**1b**) (0.25 mL, 1.96 mmol) and methyl 2-bromopropanoate (**4a**) (0.45 mL, 3.92 mmol), according to general procedure A, afforded compound **5b** (266 mg, 72%) as a light-yellow oil (hexane/EtOAc, 9:1; R*f:* 0.26) and an inseparable mixture of isomers (84:16 *syn:anti*). IR (film) *v* (cm^−1^) 3455, 2931, 2862, 1723, 1459, 1371, 1346, 1259, 1198, 1121, 1040, 935. ^1^H NMR (600 MHz, CDCl_3_) δ (ppm) common signals: 3.64 (s, 3H), 2.47 (qd, *J =* 7.2, 3.6 Hz, 1H), 1.44–1.37 (m, 2H), 1.36–1.19 (m, 7H), 0.82 (t, *J =* 6.6 Hz, 3H); characteristic signals of **5b *syn***: 3.82 (dt, *J =* 8.4, 4.2 Hz, 1H), 1.11 (d, *J =* 7.2 Hz, 3H); characteristic signals of **5b *anti***: 3.60–3.57 (m, 1H), 1.14 (d, *J =* 7.2 Hz, 3H). ^13^C NMR (150 MHz, CDCl_3_, DEPT) δ (ppm) common signals: 22.6 (CH_2_), 14.0 (CH_3_); signals of **5b *syn***: 176.7 (C), 71.8 (CH), 51.8 (CH_3_), 44.2 (CH), 33.8 (CH_2_), 31.8 (CH_2_), 25.7 (CH_2_), 10.6 (CH_3_); signals of **5b *anti***: 176.5 (C), 73.4 (CH), 51.7 (CH_3_), 45.2 (CH), 34.7 (CH_2_), 31.8 (CH_2_), 25.2 (CH_2_), 14.3 (CH_3_). IR, ^1^H NMR and ^13^C NMR spectral data of **5b *syn*** and **5b *anti*** are in agreement with literature values [38,39].

Reaction of hexanal (**1b**) (0.13 mL, 0.98 mmol) and methyl (*R*)-2-bromopropanoate (0.23 mL, 1.96 mmol), according to general procedure A, afforded compound **5b** (123 mg, 65%) as a light-yellow oil and an inseparable mixture of isomers (79:21 *syn:anti*).

*Methyl 3-hydroxy-2-methyl-5-phenylpentanoate* (**5c**). Reaction of hydrocinnamaldehyde (**1c**) (0.22 mL, 1.49 mmol) and methyl 2-bromopropanoate (**4a**) (0.34 mL, 2.98 mmol), according to general procedure A, afforded compound **5c** (232 mg, 70%) as a yellow oil (hexane/EtOAc, 1:1; R*f:* 0.30) as an inseparable mixture of isomers (81:19 *syn:anti*). IR (film) *v* (cm^−1^) 3442, 3027, 2944, 1721, 1453, 1199, 1165, 1040, 933, 745, 698, 496. ^1^H NMR (600 MHz, CDCl_3_) δ (ppm) common signals: 7.20–7.18 (m, 2H), 7.12–7.10 (m, 3H), 2.80–2.75 (m, 1H), 2.57 (ddd, *J* = 13.8, 9.7, 6.8 1H), 2.48–2.46 (m, 1H), 1.75–56 (m, 2H); characteristic signals of **5c *syn***: 3.82 (dt, *J* = 9.4, 3.6 Hz, 1H), 3.60 (s, 3H), 1.10 (d, *J* = 7.2 Hz, 3H); characteristic signals of **5c *anti***: 3.61 (s, 3H), 3.56–3.59 (m, 1H), 1.12 (d, *J* = 7.2 Hz, 3H). ^13^C NMR (150 MHz, CDCl_3_, DEPT) δ (ppm) common signals: 176.5 (C), 141.9 (C), 128.5 (CH), 128.4 (CH), 125.9 (CH); signals of **5c *syn***: 71.1 (CH), 51.8 (CH_3_), 44.5 (CH), 35.7 (CH_2_), 32.3 (CH_2_), 10.8 (CH_3_); signals of **5c *anti***: 72.7 (CH), 51.8 (CH_3_), 45.3 (CH), 36.6 (CH_2_), 31.9 (CH_2_), 14.3 (CH_3_). IR, ^1^H NMR and ^13^C NMR spectral data of **5c *syn*** and **5c *anti*** are in agreement with literature values [40].

*Ethyl 3-hydroxy-2-isopropyltridec-12-enoate* (**5d**). Reaction of undec-10-enal (**1a**) (0.24 mL, 1.19 mmol) and ethyl 2-bromo-3-methylbutanoate (**4b**) (0.39 mL, 2.38 mmol), according to the general procedure A, afforded product **5d *syn*** (209 mg, 59%) and **5d *anti*** (43 mg, 12%) (hexane/EtOAc, 9:1; *Rf syn:* 0.20, *Rf anti:* 0.30). Compound **5d *syn***, isolated as yellow oil: IR (film) *v* (cm^−1^) 3469, 2925, 2854, 1729, 1710, 1641, 1465, 1372, 1178, 1026, 909. ^1^H NMR (300 MHz, CDCl_3_) δ (ppm) 5.81 (ddt, *J* = 17.1, 10.2, 6.6 Hz, 1H, H12), 5.02–4.90 (m, 2H, H13), 4.16 (dq, *J =* 7.2, 3.6 Hz, 2H, OCH_2_CH_3_), 3.90–3.85 (m, 1H, H3), 2.34 (t, *J* = 6.8 Hz, 1H, H2), 2.23–2.11 (m, 2H, OH, CH(CH_3_)_2_), 2.04 (q, *J =* 7.2 Hz, 2H, H11), 1.46–1.28 (m, 17H), 0.99 (d, *J* = 6.3 Hz, 3H, CH(CH_3_)_2_), 0.97 (d, *J* = 6.3 Hz, 3H, CH(CH_3_)_2_). ^13^C NMR (75 MHz, CDCl_3_, DEPT) δ (ppm) 173.9 (C, C1), 139.2 (CH, C12), 114.1 (CH_2_, C13), 70.8 (CH, C3), 60.1 (CH_2_, OCH_2_CH_3_), 57.6 (CH, C2), 34.5 (CH_2_), 33.8 (CH_2_, C11), 29.5 (CH_2_), 29.4 (CH_2_), 29.1 (CH_2_), 28.9 (CH_2_), 26.6 (CH, CH(CH_3_)_2_), 26.0 (CH_2_), 21.6 (CH_3_, CH(CH_3_)_2_), 19.4 (CH_3_, CH(CH_3_)_2_), 14.4 (CH_3_, OCH_2_CH_3_). HRMS (ESI/Q-TOF) *m*/*z*: [M+H]^+^ calcd for C_18_H_35_O_3_ 299.2586; found: 299.2598. Compound **5d *anti***, isolated as yellow oil: IR (film) *v* (cm^−1^) 3522, 2926, 2855, 1712, 1463, 1377, 1176, 1027, 910. ^1^H NMR (300 MHz, CDCl_3_) δ (ppm) 5.83 (ddt, *J* = 16.8, 10.2, 6.6 Hz, 1H, H12), 5.04–4.92 (m, 2H, H13), 4.21 (q, *J* = 7.2 Hz, 2H, OCH_2_CH_3_), 3.80–3.72 (m, 1H, H3), 2.74 (d, *J* = 10.2 Hz, 1H, OH), 2.22–2.02 (m, 4H, H2, H11, CH(CH_3_)_2_), 1.46–1.25 (m, 17H), 1.04 (d, *J* = 6.4 Hz, 3H, CH(CH_3_)_2_), 0.94 (d, *J* = 6.4 Hz, 3H, (CH(CH_3_)_2_). ^13^C NMR (75 MHz, CDCl_3_, DEPT) δ (ppm) 175.7 (C, C1), 139.2 (CH, C12), 114.1 (CH_2_, C13), 70.1 (CH, C3), 60.3 (CH_2_, OCH_2_CH_3_), 57.3 (CH, C2), 36.6 (CH_2_), 33.8 (CH_2_, C11), 29.5 (CH_2_), 29.5 (CH_2_), 29.4 (CH_2_), 29.1 (CH_2_), 28.9 (CH_2_), 27.8 (CH, CH(CH_3_)_2_), 26.0 (CH_2_), 20.9 (CH_3_, CH(CH_3_)_2_), 20.6 (CH_3_, CH(CH_3_)_2_), 14.4 (CH_3_, OCH_2_CH_3_). HRMS (ESI/Q-TOF) *m*/*z*: [M+H]^+^ calcd for C_18_H_35_O_3_ 299.2586; found: 299.2602.

*Ethyl 3-hydroxy-2-isopropyloctanoate* (**5e**). Reaction of hexanal (**1b**) (0.25 mL, 1.96 mmol) and ethyl 2-bromo-3-methylbutanoate (**4b**) (0.64 mL, 3.92 mmol), according to general procedure A, afforded compounds **5e *syn*** (293 mg, 65%) and **5e *anti*** (63 mg, 14%) (hexane/EtOAc, 9:1; *Rf syn:* 0.20, *Rf anti:* 0.27). Compound **5e *syn***, isolated as yellow oil: IR (film) *v* (cm^−1^) 3469, 2958, 2932, 2873, 1728, 1709, 1465, 1373, 1228, 1177, 1025, 924. ^1^H NMR (300 MHz, CDCl_3_) δ (ppm) 4.16 (dq, *J* = 7.2, 3.5 Hz, 2H, OCH_2_CH_3_), 3.91–3.86 (m, 1H, H3), 2.36–2.32 (m, 1H, H2), 2.12–215 (m, 1H, CH(CH_3_)_2_), 1.54–1.40 (m, 3H), 1.36–1.24 (m, 5H), 1.28 (t, *J* = 7.2 Hz, 3H, OCH_2_CH_3_), 0.99 (d, *J* = 6.3 Hz, 3H, CH(CH_3_)_2_), 0.97 (d, *J* = 6.3 Hz, 3H, CH(CH_3_)_2_), 0.89 (t, *J* = 6.9 Hz, 3H, H8). ^13^C NMR (75 MHz, CDCl_3_, DEPT) δ (ppm) 173.9 (C, C1), 70.7 (CH, C3), 60.1 (CH_2_, OCH_2_CH_3_), 57.7 (CH, C2), 34.5 (CH_2_), 31.7 (CH_2_), 26.6 (CH, CH(CH_3_)_2_), 25.6 (CH_2_), 22.6 (CH_2_), 21.5 (CH_3_, CH(CH_3_)_2_), 19.3 (CH_3_, CH(CH_3_)_2_), 14.3 (CH_3_, C8), 14.0 (CH_3_, OCH_2_CH_3_). HRMS (ESI/Q-TOF) *m*/*z*: [M+H]^+^ calcd for C_13_H_27_O_3_ 231.1960; found 231.1945. Compound **5e *anti***, isolated as yellow oil: IR (film) *v* (cm^−1^) 3523, 2959, 2932, 2873, 1709, 1465, 1376, 1178, 1126, 1025, 921. ^1^H NMR (300 MHz, CDCl_3_) δ (ppm) 4.20 (q, *J* = 7.2 Hz, 2H, OCH_2_CH_3_), 3.80–3.71 (m, 1H, H3), 2.75 (d, *J* = 10.2 Hz, 1H, OH), 2.21–2.07 (m, 2H, H2, CH(CH_3_)_2_), 1.46–1.27 (m, 8H), 1.30 (t, *J* = 7.2 Hz, 3H, OCH_2_CH_3_), 1.02 (d, *J* = 6.3 Hz, 3H, CH(CH_3_)_2_), 0.93 (d, *J* = 6.3 Hz, 3H, CH(CH_3_)_2_), 0.89 (t, *J* = 6.9 Hz, 3H, H8). ^13^C NMR (75 MHz, CDCl_3_, DEPT) δ (ppm) 175.7 (C, C1), 70.0 (CH, C3), 60.3 (CH_2_, OCH_2_CH_3_), 57.3 (CH, C2), 36.5 (CH_2_), 31.7 (CH_2_), 27.7 (CH, CH(CH_3_)_2_), 25.7 (CH_2_), 22.6 (CH_2_), 20.8 (CH_3_, CH(CH_3_)_2_), 20.6 (CH_3_, CH(CH_3_)_2_), 14.3 (CH_3_, C8), 14.0 (CH_3_, OCH_2_CH_3_). HRMS (ESI/Q-TOF) *m*/*z*: [M+H]^+^ calcd for C_13_H_27_O_3_ 231.1960; found 231.1950.

tert-Butyl (2S*,3R*)-3-hydroxy-2-methyltridec-12-enoate (**5f**). Reaction of undec-10-enal (**1a**) (0.24 mL, 1.19 mmol) and *tert*-butyl 2-bromopropanoate (**4c**) (0.41 mL, 2.38 mmol), according to the general procedure A, afforded product **5f** (274 mg, 78%) isolated as light-yellow oil (hexane/EtOAc, 9:1; R*f*: 0.22). IR (film) *v* (cm^−1^) 3465, 2976, 2926, 2855, 1722, 1458, 1367, 1254, 1151, 909, 847. ^1^H NMR (300 MHz, CDCl_3_) δ (ppm) 5.83 (ddt, *J* = 17.1, 10.2, 6.6 Hz, 1H, H12), 5.04–4.92 (m, 2H, H13), 3.88–3.82 (m, 1H, H3), 2.68 (d, *J* = 4.5 Hz, 1H, OH), 2.43 (dq, *J* = 7.2, 3.6 Hz, 1H, H2), 2.05 (m, 2H, H11), 1.48 (s, 9H, C(CH_3_)_3_), 1.41–1.30 (m, 14H), 1.15 (d, *J* = 7.2 Hz, 3H). ^13^C NMR (75 MHz, CDCl_3_, DEPT) δ (ppm) 175.8 (C, C1), 139.2 (CH, C12), 114.1 (CH_2_, C13), 80.9 (C, C(CH_3_)_3_), 71.8 (CH, C3), 44.9 (CH, C2), 33.8 (CH_2_), 33.7 (CH_2_, C11), 29.6 (CH_2_), 29.5 (CH_2_), 29.4 (CH_2_), 29.1 (CH_2_), 28.9 (CH_2_), 28.1 (CH_3_, C(CH_3_)_3_), 26.0 (CH_2_), 10.8 (CH_3_). HRMS (ESI/Q-TOF) *m*/*z*: [M+H]^+^ calcd for C_18_H_35_O_3_ 299.2586; found: 299.2564.

tert-Butyl (2S*,3R*)-3-hydroxy-2-methyloctanoate (**5g**). Reaction of hexanal (**1b**) (0.25 mL, 1.96 mmol) and *tert*-butyl 2-bromopropanoate (**4c**) (0.67 mL, 3.92 mmol), according to general procedure A, afforded compound **5g** (363 mg, 80%), isolated as yellow oil (hexane/EtOAc, 9:1; R*f*: 0.22). IR (film) *v* (cm^−1^) 3437, 2931, 2865, 1719, 1459, 1367, 1255, 1152, 1024, 847. ^1^H NMR (300 MHz, CDCl_3_) δ (ppm) 3.88–3.82 (m, 1H, H3) 2.69 (br s, 1H, OH), 2.43 (dq, *J* = 7.2, 3.6 Hz, 1H, H2), 1.48 (s, 9H, C(CH_3_)_3_), 1.44–1.27 (m, 8H), 1.15 (d, *J* = 7.2 Hz, 3H), 0.90 (t, *J* = 6.9 Hz, 3H, H8). ^13^C NMR (75 MHz, CDCl_3_, DEPT) δ (ppm) 175.8 (C, C1), 80.9 (C, C(CH_3_)_3_), 71.8 (CH, C3), 44.9 (CH, C2), 33.7 (CH_2_), 31.8 (CH_2_), 28.1 (CH_3_, C(CH_3_)_3_), 25.7 (CH_2_), 22.6 (CH_2_), 14.0 (CH_3_, C8), 10.8 (CH_3_). HRMS (ESI/Q-TOF) *m*/*z*: [M+H]^+^ calcd for C_13_H_27_O_3_ 231.1960; found.231.1974.

### 3.3. General Procedure B for Ti-Induced Reformatsky Reaction

Under an Ar atmosphere, dry THF (8 mL/1.4 mmol of aldehyde) previously deoxygenated was added to a miscellany of CpTiCl_3_ (1 equiv) and Mn dust (2 equiv) resulting in a green suspension. A solution of aldehyde (1 equiv) and α-halo ester or 2-iodoacetonitrile (2 equiv) in THF (2 mL/1.4 mmol of aldehyde) was added dropwise, and the reaction was stirred overnight. The mixture was filtered, diluted with EtOAc, washed with HCl 3% and brine, and dried (anhydrous MgSO_4_), and the solvent was removed. Products were purified by silica gel flash column chromatography (hexane/EtOAc mixtures).

tert-Butyl (2R*,3R*)-3-hydroxy-3-(3-methoxyphenyl)-2-methylpropanoate (**5h**). Reaction of 3-methoxybenzaldehyde (**1d**) (0.18 mL, 1.47 mmol) and *tert*-butyl 2-bromopropanoate (**4c**) (0.50 mL, 2.94 mmol), according to the general procedure B, afforded compound **5h** (308 mg, 78%) isolated as colorless oil (hexane/EtOAc, 7:3; R*f*: 0.40). ^1^H NMR spectral data are in agreement with literature values [41]. IR (film) *v* (cm^−1^) 3479, 2976, 2937, 2838, 1714, 1596, 1485, 1458, 1254, 1151, 1032, 845, 786, 700.^13^C NMR (75 MHz, CDCl_3_, DEPT) δ (ppm) 175.4 (C), 159.6 (C), 143.3 (C), 129.2 (CH), 118.5 (CH), 113.0 (CH), 111.7 (CH), 81.1 (C), 73.6 (CH), 55.2 (CH_3_), 47.0 (CH), 28.0 (CH_3_), 11.0 (CH_3_). HRMS (ESI/Q-TOF) *m*/*z*: [M+H]^+^ calcd for C_14_H_21_O_3_ 237.1491; found: 237.1509.

*3-Hydroxyoctanenitrile* (**9**). Reaction of hexanal (**1b**) (0.13 mL, 0.98 mmol) and 2-iodoacetonitrile (**8**) (0.33 g, 1.96 mmol), according to general procedure B, afforded compound **9** (131 mg, 95%) isolated as yellow oil (hexane/EtOAc, 8:2; R*f*: 0.22). ^1^H NMR and ^13^C NMR spectral data are in agreement with literature values [42]. IR (film) *v* (cm^−1^) 3446, 2930, 2863, 2253, 1714, 1461, 1415, 1267, 1078, 1041, 911, 731.

*3-Hydroxy-3-(3-methoxyphenyl)propanenitrile* (**10**) and *1,2-bis(3-methoxyphenyl)ethane-1,2-diol*. Reaction of 3-methoxybenzaldehyde (**1d**) (0.10 g, 0.74 mmol) and 2-iodoacetonitrile (**8**) (0.25 g, 1.48 mmol), according to the general procedure B, afforded products **10** (32 mg, 32%) and 1,2-bis(3-methoxyphenyl)ethane-1,2-diol (36 mg, 18%). Compound **10** was isolated as a light-yellow oil (hexane/EtOAc, 1:1; R*f*: 0.40). ^1^H NMR and ^13^C NMR spectral data are in agreement with literature values [43]. IR (film) *v* (cm^−1^) 3434, 3005, 2924, 2842, 2255, 1711, 1594, 1460, 1259, 1154, 1039, 788, 698. 1,2-bis(3-methoxyphenyl)ethane-1,2-diol was isolated as a pale-yellow solid (*dl*/*meso* mixture), and spectral data are in agreement with literature values [35].

### 3.4. Reduction of the β-Hydroxy Esters

A solution of **5f** or **5g** (1 equiv) in dry THF (15 mL/1.5 mmol) at 0 °C was added dropwise to a solution of LiAlH_4_ (4 equiv) in dry THF (15 mL/1.5 mmol). The mixture was stirred under N_2_ at room temperature for 7 h. Then, Na_2_SO_4_·10H_2_O was added at 0 °C and diluted with Et_2_O. The solution was filtered, and the two phases were separated. The organic phase was dried over MgSO_4_, filtered and concentrated in vacuum. The products were purified by flash chromatography (hexane/EtOAc mixtures).

(2R*,3R*)-2-methyltridec-12-ene-1,3-diol (269 mg, 77%) was isolated as colorless oil (hexane/EtOAc, 6:4; R*f*: 0.30). IR (film) *v* (cm^−1^) 3331, 2924, 2853, 1641, 1461, 1089, 1028, 911, 908, 722. ^1^H NMR (300 MHz, CDCl_3_) δ (ppm) 5.79 (ddt, *J* = 17.1, 10.2, 6.9 Hz, 1H), 4.99–4.89 (m, 2H), 3.79–3.73 (m, 1H), 3.62–3.60 (m, 2H), 2.05–1.98 (m, 2H), 1.76–1.70 (m, 1H), 1.48–1.27 (m, 14H), 0.85 (d, *J* = 7.0 Hz, 3H). ^13^C NMR (75 MHz, CDCl_3_, DEPT) δ (ppm) 139.1 (CH), 114.1 (CH_2_), 73.7 (CH), 66.4 (CH_2_), 38.9 (CH), 33.9 (CH_2_), 33.8 (CH_2_), 29.7 (CH_2_), 29.6 (CH_2_), 29.5 (CH_2_), 29.1 (CH_2_), 28.9 (CH_2_), 26.3 (CH_2_), 10.0 (CH_3_). HRMS (ESI/Q-TOF) *m*/*z*: [M+H]^+^ calcd for C_14_H_29_O_2_ 229.2168; found: 229.2154.

(2R*,3R*)-2-methyloctane-1,3-diol (82 mg, 49%) was isolated as colorless oil (hexane/EtOAc, 6:4; R*f*: 0.36). IR (film) *v* (cm^−1^) 3351, 2928, 2863, 1460, 1370, 1227, 1026, 979. ^1^H NMR (300 MHz, CDCl_3_) δ (ppm) 3.80–3.75 (m, 1H), 3.63–3.62 (m, 2H), 1.77–1.70 (m, 1H), 1.49–1.21 (m, 8H), 0.89–0.84 (m, 6H). ^13^C NMR (75 MHz, CDCl_3_, DEPT) δ (ppm) 74.0 (CH), 66.6 (CH_2_), 39.0 (CH), 33.9 (CH_2_), 31.9 (CH_2_), 25.9 (CH_2_), 22.6 (CH_2_), 14.0 (CH_3_), 10.0 (CH_3_). HRMS (ESI/Q-TOF) *m*/*z*: [M+H]^+^ calcd for C_9_H_21_O_2_ 161.1542; found 161.1558.

### 3.5. Synthesis of the Ketal Derivatives

To a solution of the diol ((2*R*,3*R*)-2-methyltridec-12-ene-1,3-diol or (2*R*,3*R*)-2-methyloctane-1,3-diol (1 equiv)) and 2,2-dimethoxypropane (3 equiv) in dry DCM (10 mL/0.3 mmol), camphor sulfonic acid (0.3 equiv) was added at 0 °C. The mixture was stirred under N_2_ at room temperature overnight. Then, an aqueous solution of NaHCO_3_ sat. was added. The aqueous layers were extracted with EtOAc, and the combined organic layers were dried over MgSO_4_, filtered and concentrated in vacuum. The products were purified by flash chromatography (hexane/EtOAc mixtures).

(4R*,5S*)-4-(dec-9-en-1-yl)-2,2,5-trimethyl-1,3-dioxane (**6**) (58 mg, 72%) isolated as colorless oil (hexane/EtOAc, 8:2; R*f*: 0.26). IR (film) *v* (cm^−1^) 2991, 2925, 2854, 1641, 1460, 1378, 1271, 1242, 1196, 1103, 1006, 907, 850. ^1^H NMR (500 MHz, CDCl_3_) δ (ppm) 5.82 (ddt, *J* = 17.0, 10.2, 6.7 Hz, 1H, H9′), 5.00 (ddt, *J* = 17.0, 2.2, 1.6 Hz, 1H, H10′a), 4.94 (ddt, *J* = 10.2, 2.2, 1.3 Hz, 1H, H10′b), 4.11 (dd, *J* = 11.5, 2.8 Hz, 1H, H6_ax_), 3.91 (ddd, *J* = 7.6, 5.5, 2.5 Hz, 1H, H4), 3.59 (dd, *J* = 11.5, 1.6 Hz, 1H, H6_eq_), 2.07–2.03 (m, 2H, H8′), 1.45 (s, 3H, CH_3ax_), 1.40 (s, 3H, CH_3eq_), 1.29–1.20 (m, 15H), 1.06 (d, *J* = 6.9 Hz, 3H). ^13^C NMR (75 MHz, CDCl_3_, DEPT) δ (ppm) 139.2 (CH, C9′), 114.1 (CH_2_, C10′), 98.5 (C, C2), 71.5 (CH, C4), 67.0 (CH_2_, C6), 33.8 (CH_2_, C8′), 32.9 (CH_2_), 31.6 (CH_,_ C5), 29.8 (CH_3_, CH_3eq_), 29.6 (CH_2_), 29.5 (CH_2_), 29.4 (CH_2_), 29.1 (CH_2_), 28.9 (CH_2_), 25.2 (CH_2_), 19.1 (CH_3_, CH_3ax_), 10.5 (CH_3_). HRMS (ESI/Q-TOF) *m*/*z*: [M+H]^+^ calcd for C_17_H_33_O_2_ 269.2481; found: 269.2475.

(4R*,5R*)-2,2,5-trimethyl-4-pentyl-1,3-dioxane (**7**) (51 mg, 46%) isolated as colorless oil (hexane/EtOAc, 8:2; R*f*: 0.30). IR (film) *v* (cm^−1^) 2932, 2861, 1460, 1378, 1270, 1240, 1196, 1105, 1006, 860. ^1^H NMR (300 MHz, CDCl_3_) δ (ppm) 4.12 (dd, *J* = 11.4, 3.0 Hz, 1H, H6_ax_), 3.95–3.90 (m, 1H, H4), 3.60 (dd, *J* = 11.4, 1.7 Hz, 1H, H6_eq_), 1.46 (s, 3H, CH_3ax_), 1.41 (s, 3H, CH_3eq_), 1.51–1.24 (m, 9H), 1.07 (d, *J* = 6.9 Hz, 3H, CH_3_), 0.93–0.88 (m, 3H, H5′). ^13^C NMR (75 MHz, CDCl_3_, DEPT) δ (ppm) 98.5 (C, C2), 71.5 (CH, C4), 67.0 (CH_2_, C6), 32.8 (CH_2_), 31.8 (CH_2_), 31.6 (CH, C5), 29.8 (CH_3_, CH_3eq_), 24.9 (CH_2_), 22.6 (CH_2_), 19.1 (CH_3_, CH_3ax_), 14.1 (CH_3_, C5′), 10.5 (CH_3_). HRMS (ESI/Q-TOF) *m*/*z*: [M+H]^+^ calcd for C_12_H_25_O_2_ 201.1855 found 201.1849.

## 4. Conclusions

In summary, we have demonstrated that the half-sandwich titanium(III) complex CpTiCl_2_ represents a highly effective alternative to the Nugent–RajanBabu reagent in Reformatsky-type couplings. The methodology affords β-hydroxy esters and nitriles in consistently high yields and, more importantly, with a remarkable diastereoselective bias towards the *syn* isomer. This outcome clearly contrasts with the limited or *anti*-selective stereocontrol previously reported for Cp_2_TiCl-mediated systems. The improved performance of CpTiCl_2_ can be attributed to its lower steric hindrance, greater Lewis acidity, and increased coordination flexibility, which favor a Zimmerman–Traxler-type transition state that enforces *syn* selectivity.

The scope of the reaction, encompassing aliphatic, unsaturated, and aromatic aldehydes as well as α-bromoesters and α-iodonitriles, underscores the robustness and synthetic utility of this titanium system. Taken together, these results reveal CpTiCl_2_ as a powerful, sustainable, and stereoselective catalyst for Reformatsky reactions, with potential implications for the construction of complex molecular architectures.

## Figures and Tables

**Table 1:** Titanium catalyzed Reformatsky reaction with different substrates.

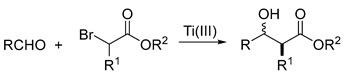					
Entry	Aldehyde	Bromoester	Hydroxyester	*Syn:Anti* Ratio	Yield ^(c)^
1 ^(a)^	**1a**	**4a** R^1^ = R^2^ = Me	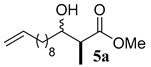	82:18	89%
2 ^(a)^	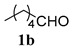	**4a** R^1^ = R^2^ = Me	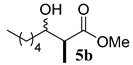	84:16	72%
3 ^(a)^	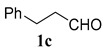	**4a** R^1^ = R^2^ = Me	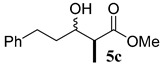	81:19	70%
4 ^(a)^	**1a**	**4b** R^1^ = *i*Pr R^2^ = Et	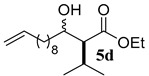	83:17	71%
5 ^(a)^	**1b**	**4b** R^1^ = *i*Pr R^2^ = Et	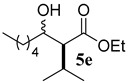	82:18	79%
6 ^(a)^	**1a**	**4c** R^1^ = Me R^2^ = *t*Bu	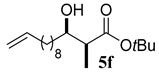	100:0	78%
7 ^(a)^	**1b**	**4c** R^1^ = Me R^2^ = *t*Bu	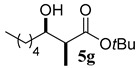	100:0	80%
8 ^(b)^	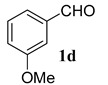	**4c** R^1^ = Me R^2^ = *t*Bu	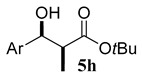	100:0	78%

^(a)^ **Procedure A**: CpTiCl_3_ (0.1 equiv), Mn (2 equiv), Me_3_SiBr (3 equiv), THF, r.t. ^(b)^ **Procedure B**: CpTiCl_3_ (1 equiv), Mn (2 equiv), THF, r.t. ^(c)^ overall yield after purification by column chromatography.

## Data Availability

Data is contained within the article and Supplementary Materials.

## Supplementary Materials

The following supporting information can be downloaded at: https://www.mdpi.com/article/10.3390/molecules30193893/s1, Figures S1–S70: NMR and IR spectra of compounds (**3**), (**5a**–**5h**), (**6**–**7**), (**9**–**10**), 1,2-bis(3-methoxyphenyl)ethane-1,2-diol, (2*R**,3*R**)-2-methyltridec-12-ene-1,3-diol and (2*R**,3*R**)-2-methyloctane-1,3-diol.
