# Supplementary material for: Diastereoselective Reformatsky Reaction Mediated by Dichlorocyclopentadienyltitanium(III)
Source: Molecules. 2025 Sep 26;30(19):3893. doi: 10.3390/molecules30193893 (PMC12525768; doi:10.3390/molecules30193893)
Supplement: Supplementary file 1 [file molecules-30-03893-s001.zip › molecules-3869609-supplementary.pdf]

Supporting Information

DDiastereoselective Reformatsky Reaction Mediated by Dichlorocyclopentadienyltitanium(III)

Josefa L. López-Martínez, Irene Torres-García, Manuel Muñoz-Dorado, Miriam Álvarez-Corral * and Ignacio Rodríguez-García *

Organic Chemistry, University of Almería, CIAIMBITAL, 04120 Almería, Spain; pepaloma91@hotmail.com (J.L.L.-M.); itg491@ual.es (I.T.-G.); mdorado@ual.es (M.M.-D.)

*Correspondence: malvarez@ual.es (M.Á.-C.); irodrigu@ual.es (I.R.-G.)

NMR AND IR SPECTRA

^{1}H NMR, DEPT 135, ^{13}C NMR and IR of **3**........1
^{1}H NMR, DEPT 135, ^{13}C NMR and IR of **5a**........4
^{1}H NMR, DEPT 135, ^{13}C NMR and IR of **5b**........7
^{1}H NMR, DEPT 135, ^{13}C NMR and IR of **5c**........10
^{1}H NMR, DEPT 135, ^{13}C NMR, HSQC and IR of **5d** ***syn***........13
^{1}H NMR, DEPT 135, ^{13}C NMR, HSQC and IR of **5d** ***anti***........17
^{1}H NMR, DEPT 135, ^{13}C NMR, HSQC, NOESY 1D and IR of **5e** ***syn***........21
^{1}H NMR, DEPT 135, ^{13}C NMR, HSQC, NOESY 1D and IR of **5e** ***anti***........26
^{1}H NMR, DEPT 135, ^{13}C NMR, HSQC, NOESY 1D and IR of **5f**........31
^{1}H NMR, DEPT 135, ^{13}C NMR, HSQC, NOESY 1D and IR of **5g**........36
^{1}H NMR, DEPT 135, ^{13}C NMR and IR of **5h**........41
^{1}H NMR, DEPT 135, ^{13}C NMR and IR of **9**........44
^{1}H NMR, DEPT 135, ^{13}C NMR and IR of **10**........47
^{1}H NMR and DEPT 135, ^{13}C NMR of 1,2-bis(3-methoxyphenyl)ethane-1,2-diol........50
^{1}H NMR, DEPT 135, ^{13}C NMR and IR of (2*R**,3*R**)-2-methyltridec-12-ene-1,3-diol........52
^{1}H NMR, DEPT 135, ^{13}C NMR, HSQC, NOESY 2D and NOESY 1D of **6**........55
^{1}H NMR, DEPT 135, ^{13}C NMR and IR of (2*R**,3*R**)-2-methyloctane-1,3-diol........61
^{1}H NMR, DEPT 135, ^{13}C NMR, HSQC, NOESY 2D, NOESY 1D and IR of **7**........64

^{1}H NMR, DEPT 135, ^{13}C NMR and IR of 3

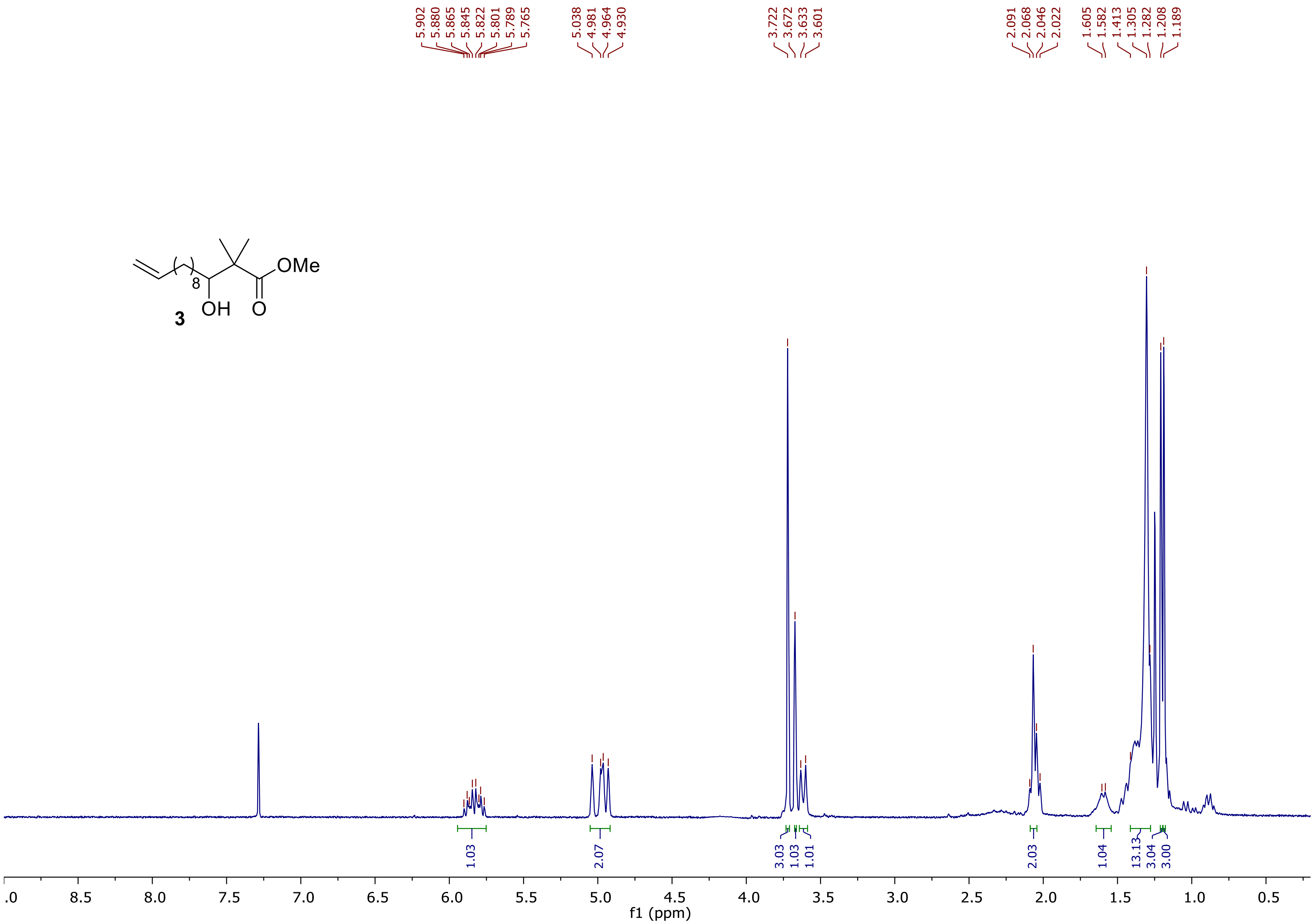

Figure S1

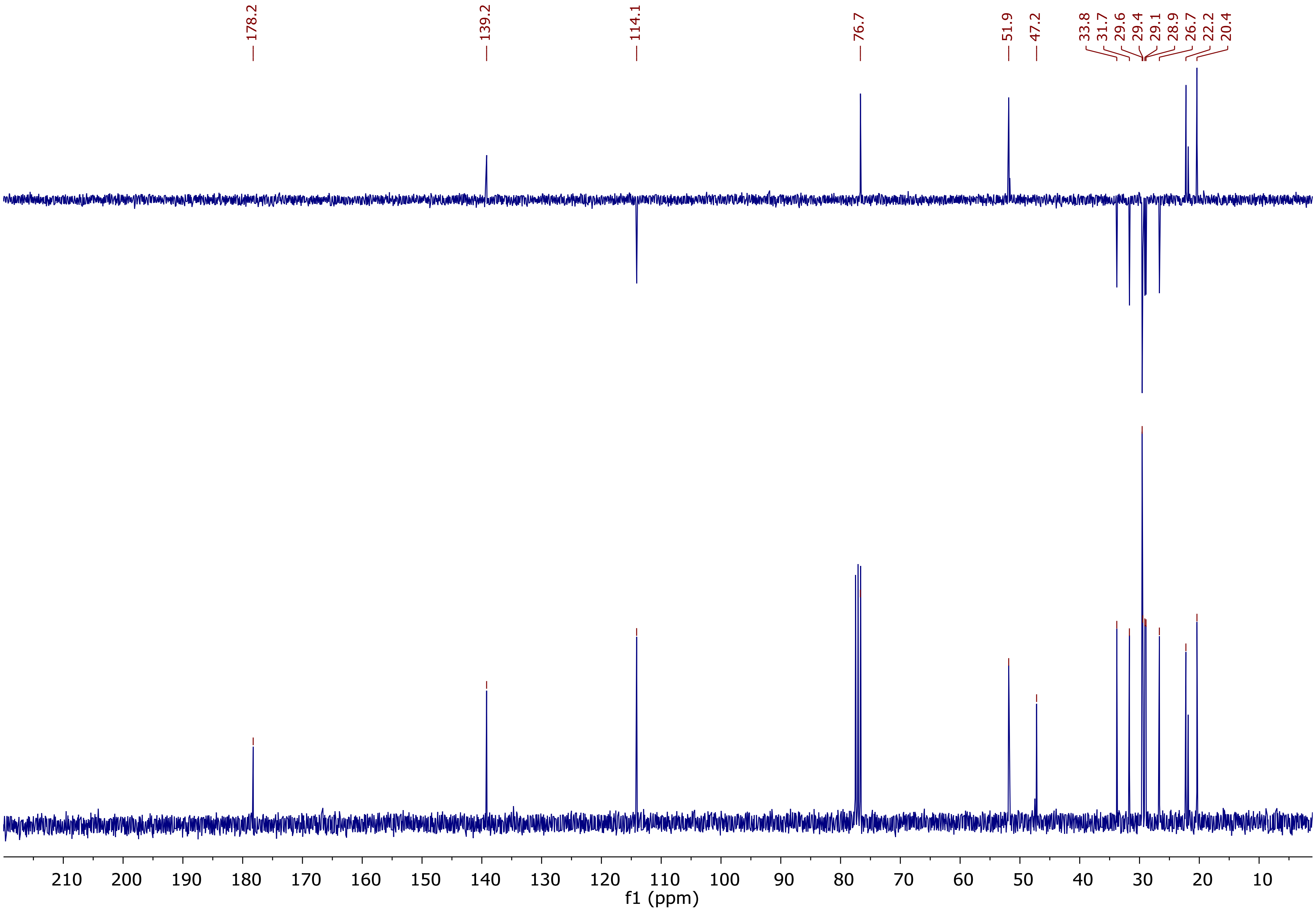

178.2
139.2
114.1
76.7
51.9
47.2
33.8
31.7
29.6
29.4
29.1
28.9
26.7
22.2
20.4
210
200
190
180
170
160
150
140
130
120
110
100
90
80
70
60
50
40
30
20
10
f1 (ppm)

Figure S2

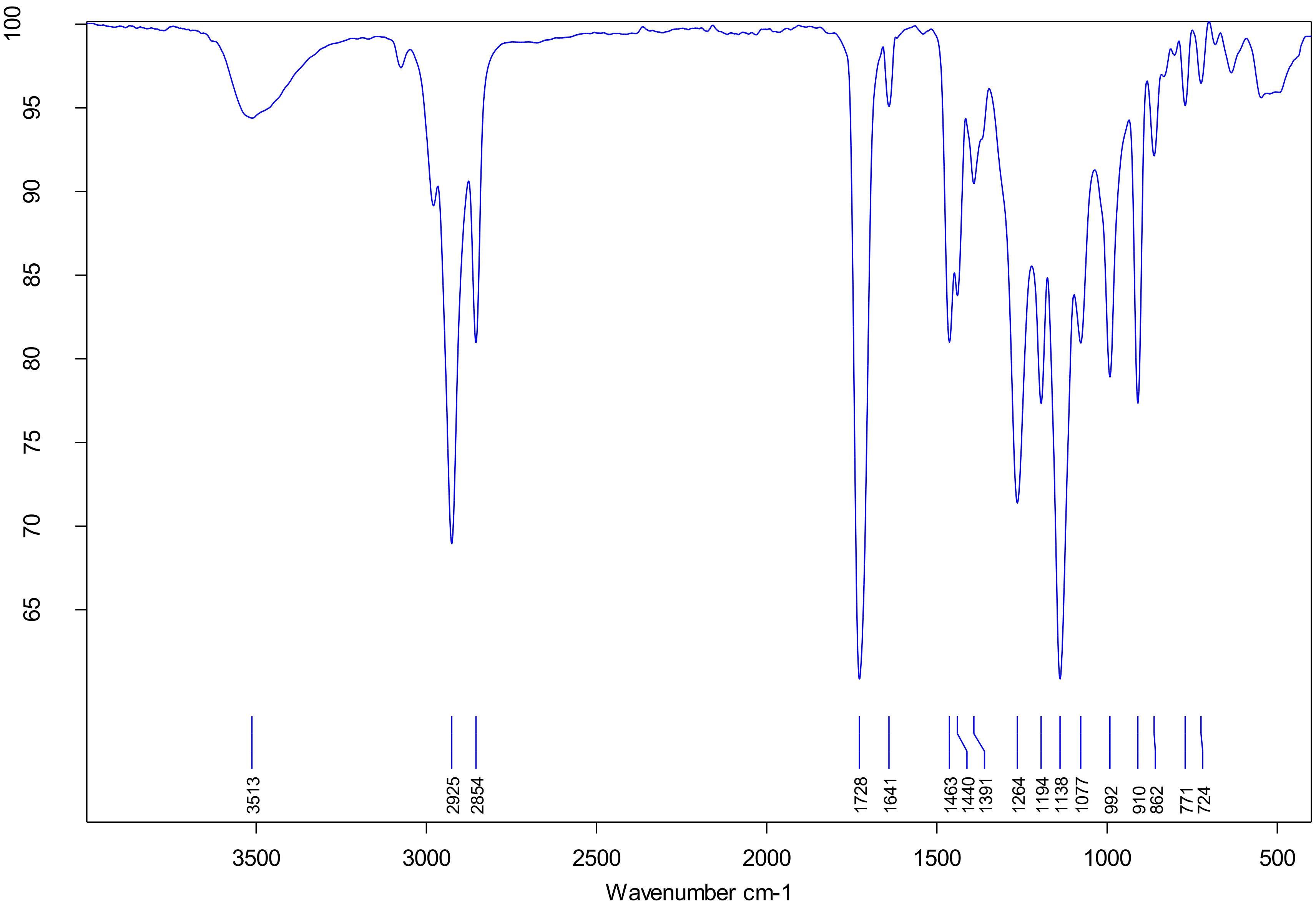
100
95
90
85
80
75
70
65
3513
2925
2854
1728
1641
1463
1440
1391
1264
1194
1138
1077
992
910
862
771
724
3500
3000
2500
2000
1500
1000
500
Wavenumber cm-1

Figure S3

^{1}H NMR, DEPT 135, ^{13}C NMR and IR of 5a

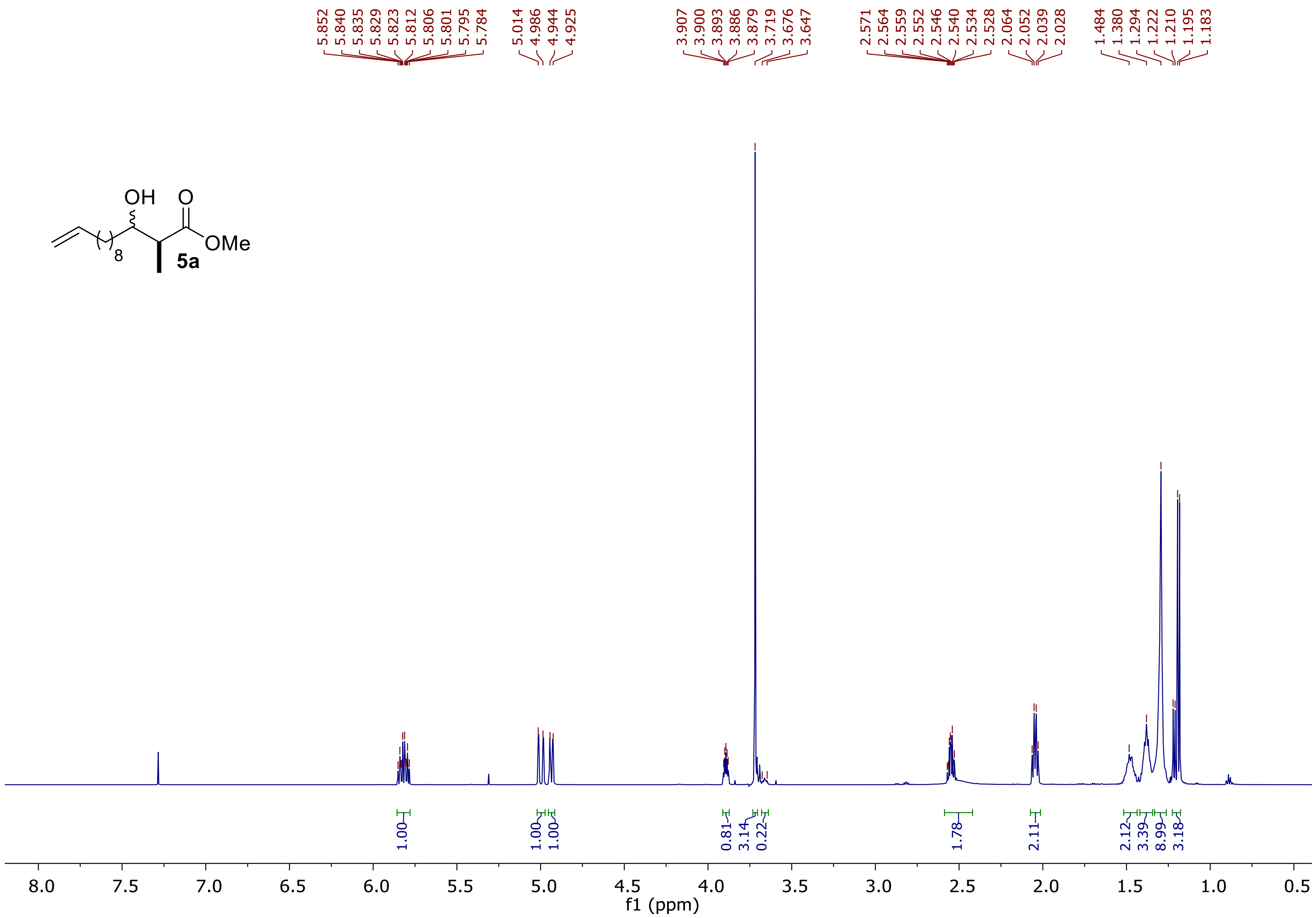

Figure S4

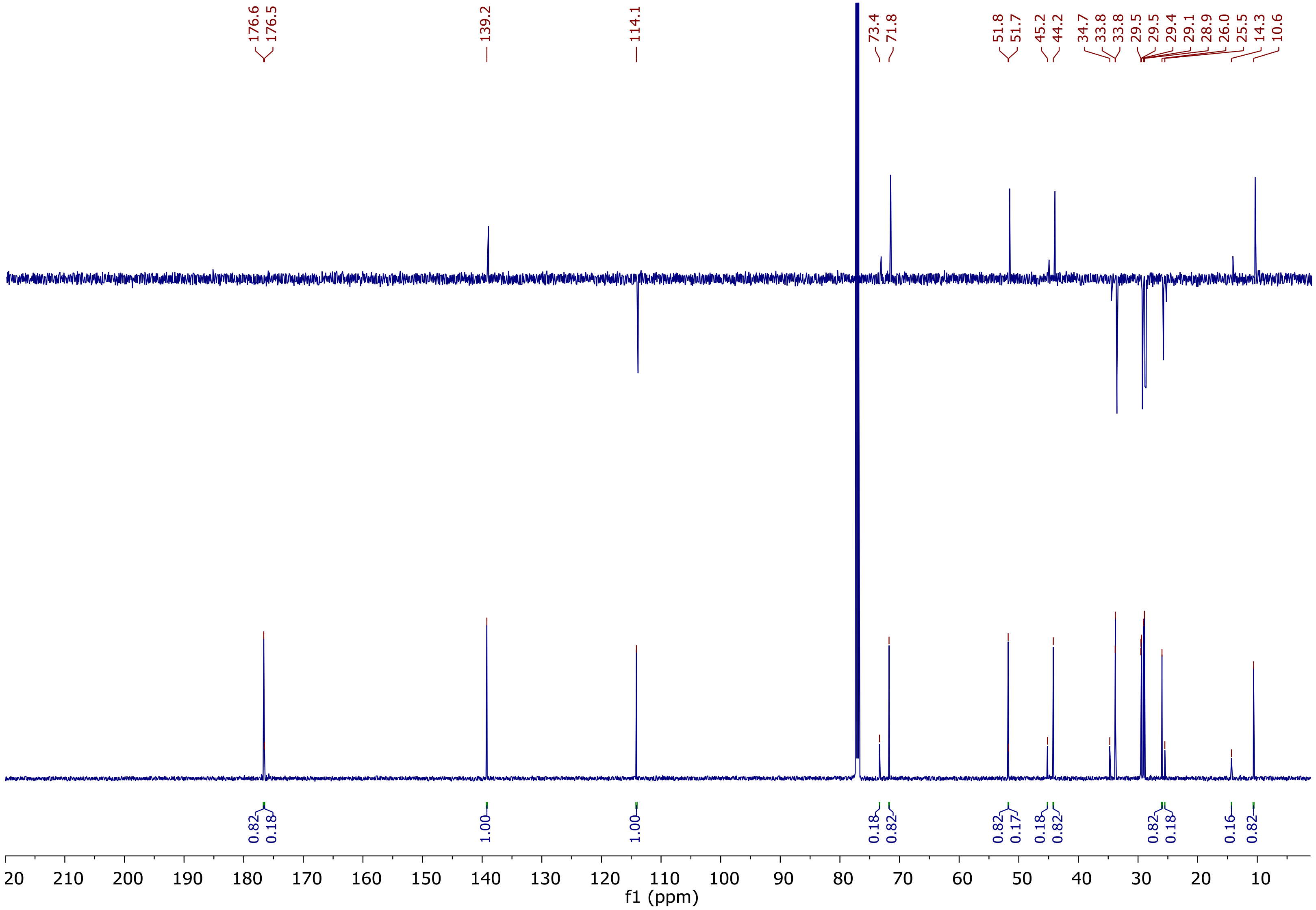

176.6
176.5
139.2
114.1
73.4
71.8
51.8
51.7
45.2
44.2
34.7
33.8
33.8
29.5
29.5
29.4
29.1
28.9
26.0
25.5
14.3
10.6
0.82
0.18
1.00
1.00
0.18
0.82
0.82
0.17
0.18
0.82
0.82
0.18
0.16
0.82
20 210 200 190 180 170 160 150 140 130 120 110 100 90 80 70 60 50 40 30 20 10
f1 (ppm)

Figure S5

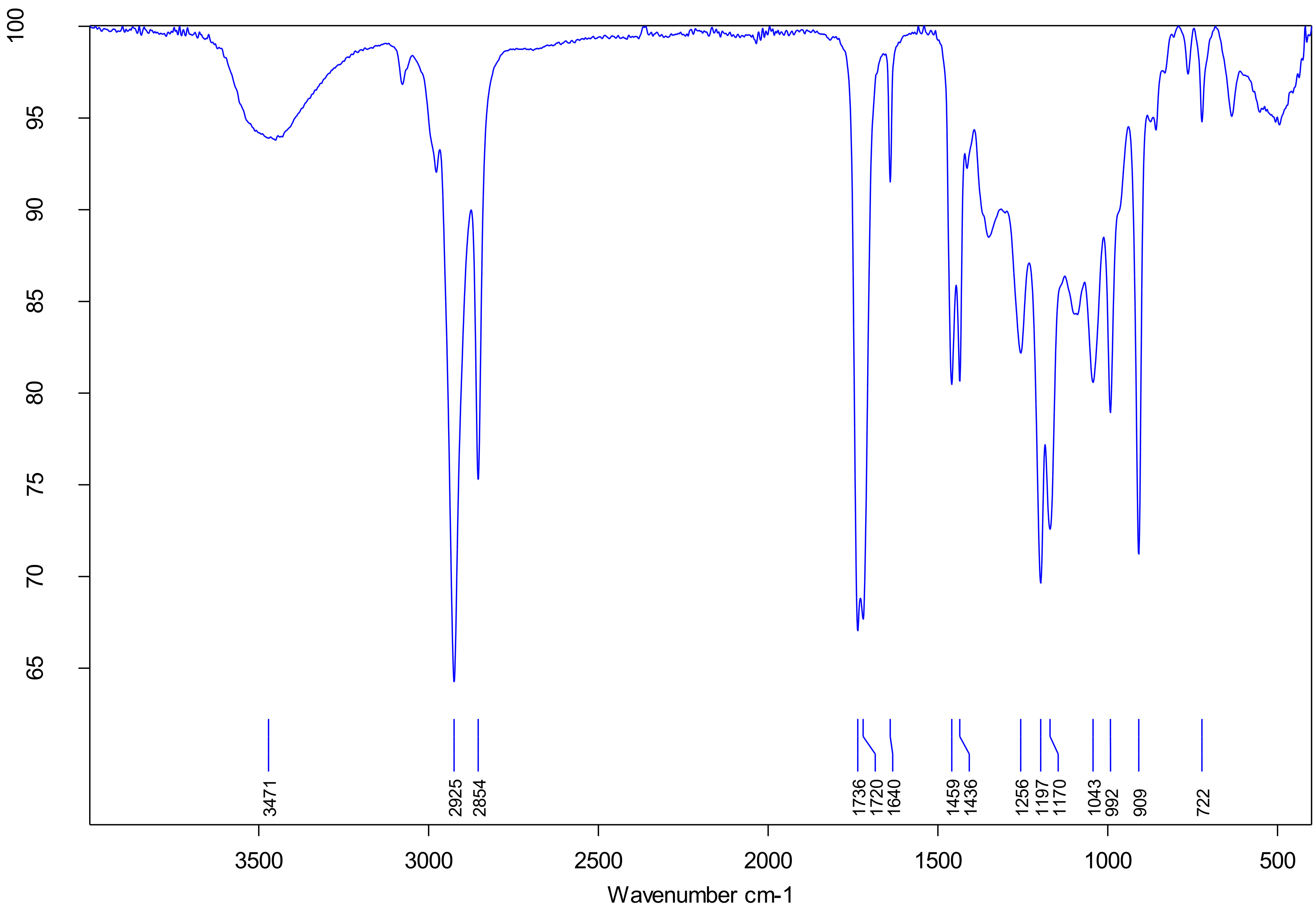

100
95
90
85
80
75
70
65
3471
2925
2854
1736
1720
1640
1459
1436
1256
1197
1170
1043
992
909
722
3500
3000
2500
2000
1500
1000
500
Wavenumber cm-1

Figure S6

^{1}H NMR, DEPT 135, ^{13}C NMR and IR of 5b

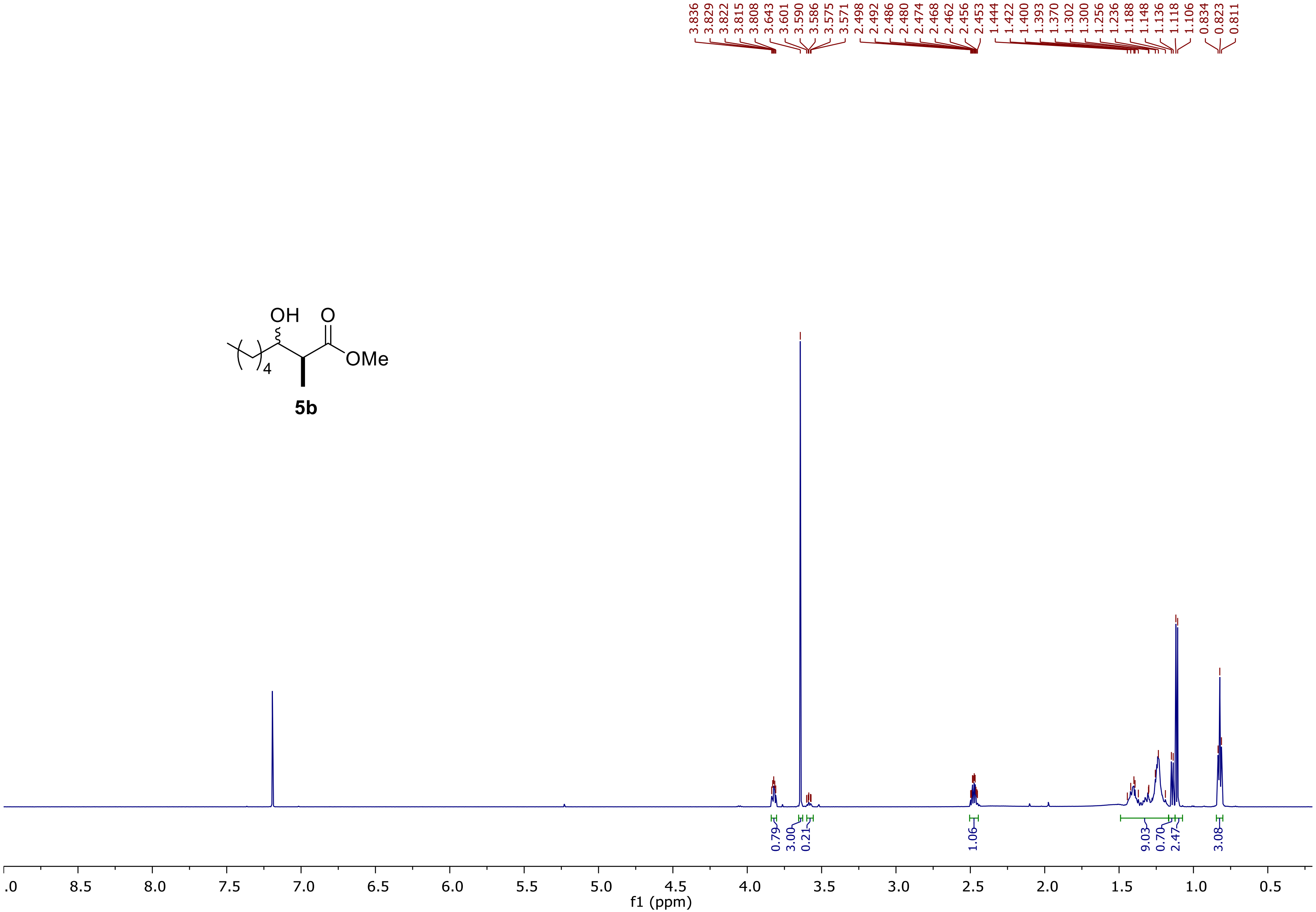

Figure S7

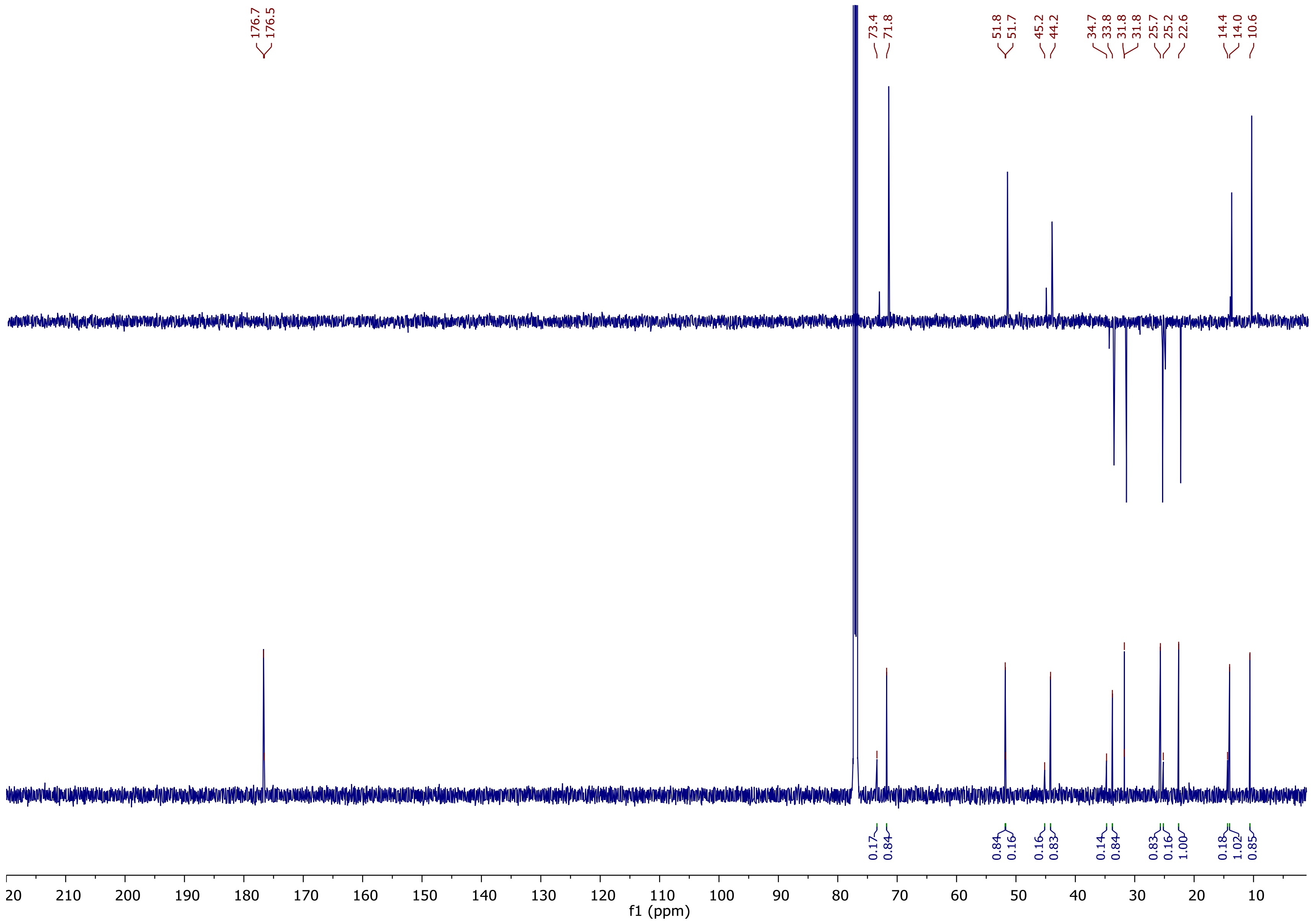
176.7
176.5
73.4
71.8
51.8
51.7
45.2
44.2
34.7
33.8
31.8
31.8
25.7
25.2
22.6
14.4
14.0
10.6
0.17
0.84
0.84
0.16
0.16
0.83
0.14
0.84
0.83
0.16
1.00
0.18
1.02
0.85
20
210
200
190
180
170
160
150
140
130
120
110
100
90
80
70
60
50
40
30
20
10
f1 (ppm)

Figure S8

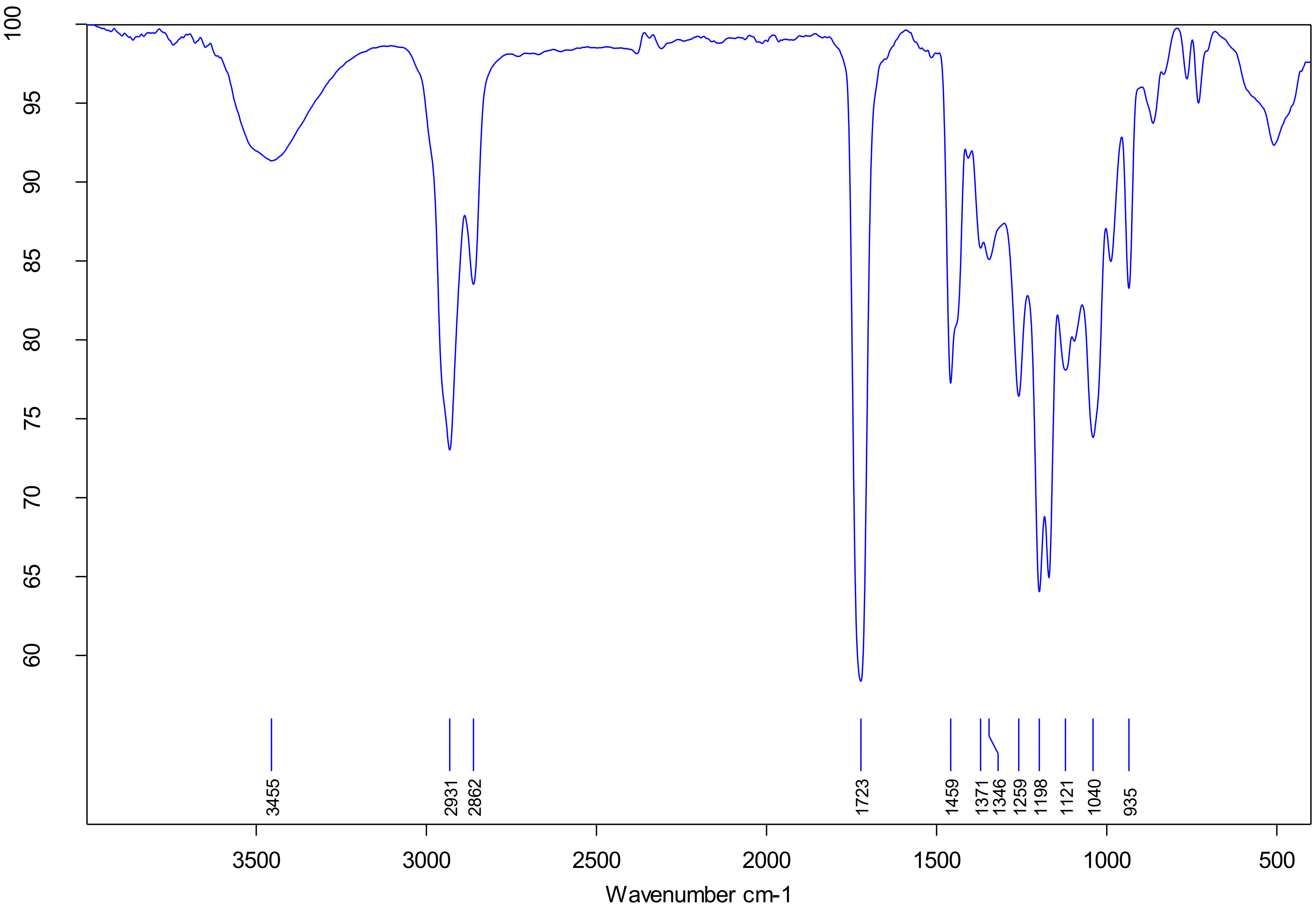
100
95
90
85
80
75
70
65
60
3455
2931
2862
1723
1459
1371
1346
1259
1198
1121
1040
935
3500
3000
2500
2000
1500
1000
500
Wavenumber cm-1

Figure S9

^{1}H NMR, DEPT 135, ^{13}C NMR and IR of 5c

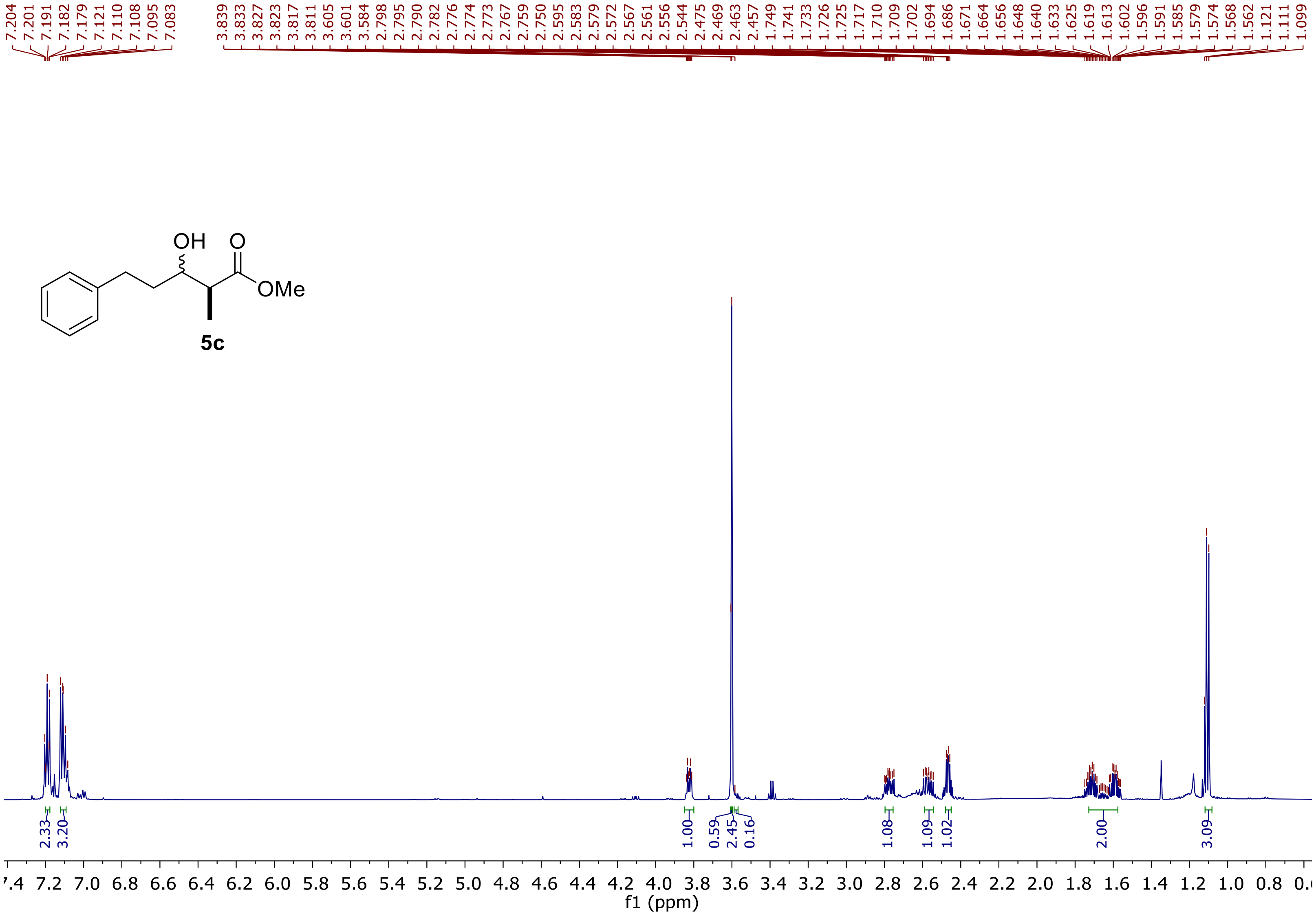

Figure S10

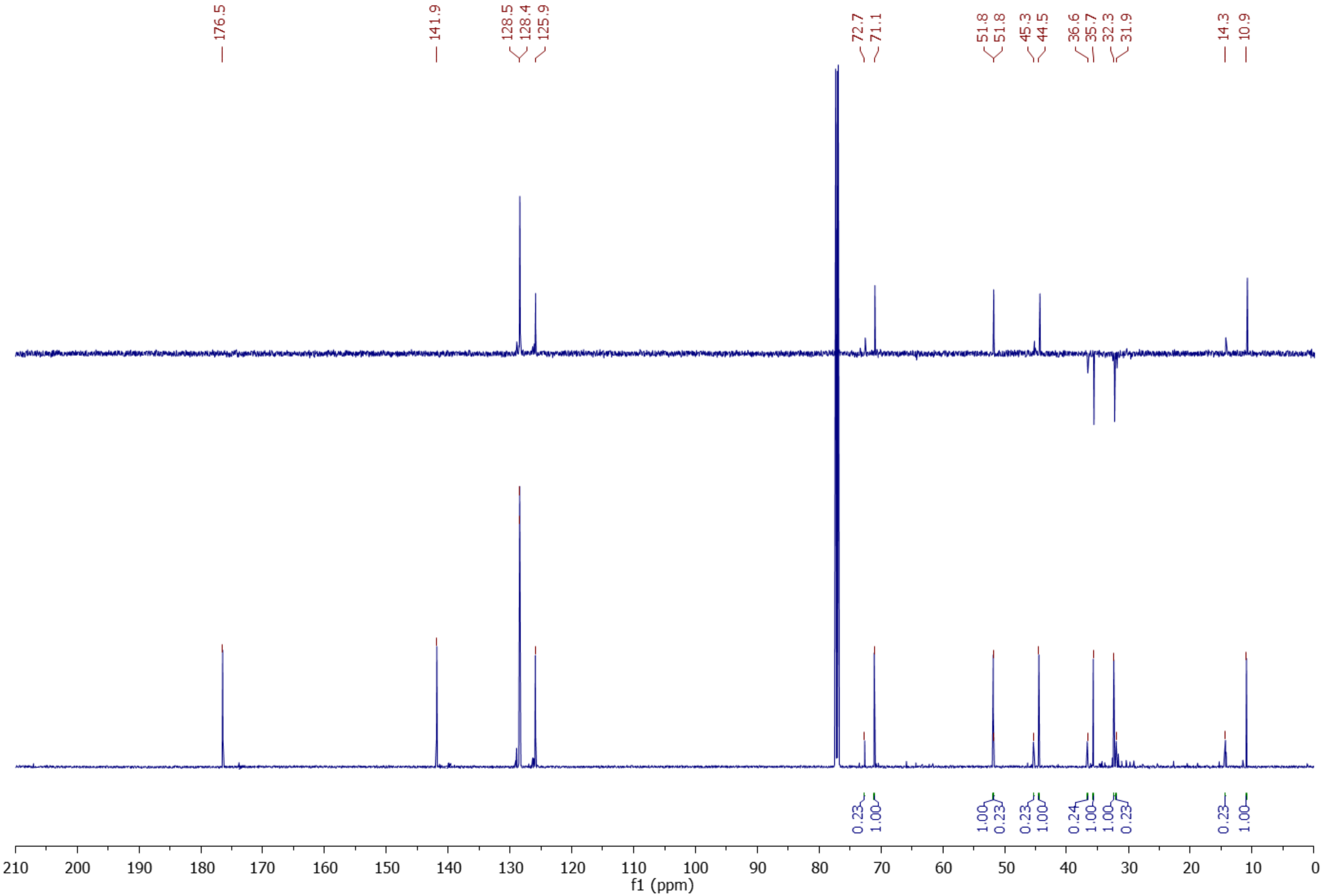
176.5
141.9
128.5
128.4
125.9
72.7
71.1
51.8
51.8
45.3
44.5
36.6
35.7
32.3
31.9
14.3
10.9
0.23
1.00
1.00
0.23
0.23
1.00
0.24
1.00
1.00
0.23
0.23
1.00
210
200
190
180
170
160
150
140
130
120
110
100
90
80
70
60
50
40
30
20
10
0
f1 (ppm)

Figure S11

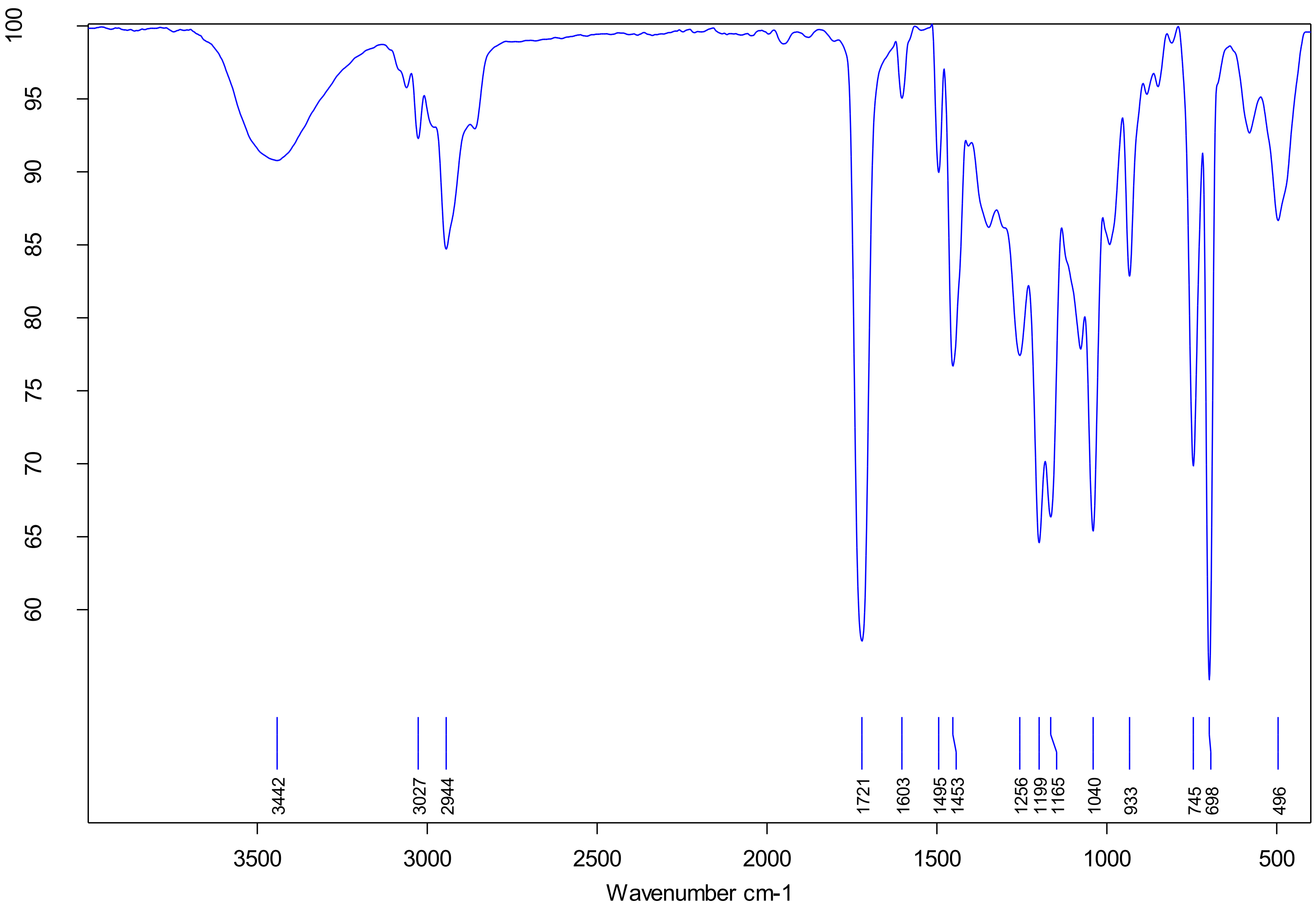

100
95
90
85
80
75
70
65
60
3442
3027
2944
1721
1603
1495
1453
1256
1199
1165
1040
933
745
698
496
3500
3000
2500
2000
1500
1000
500
Wavenumber cm-1

Figure S12

^{1}H NMR, DEPT 135, ^{13}C NMR, HSQC and IR of 5d *syn*

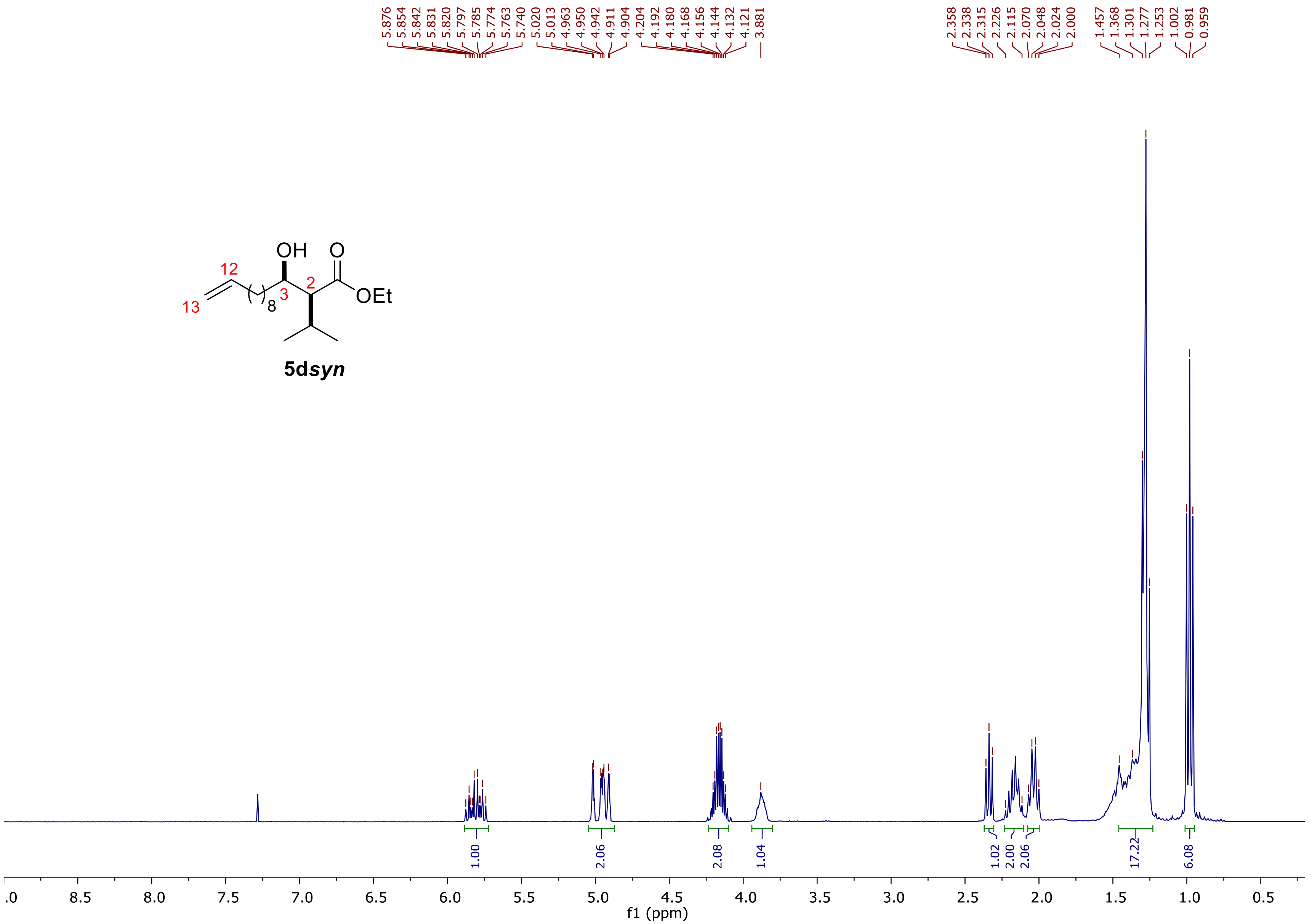

Figure S13

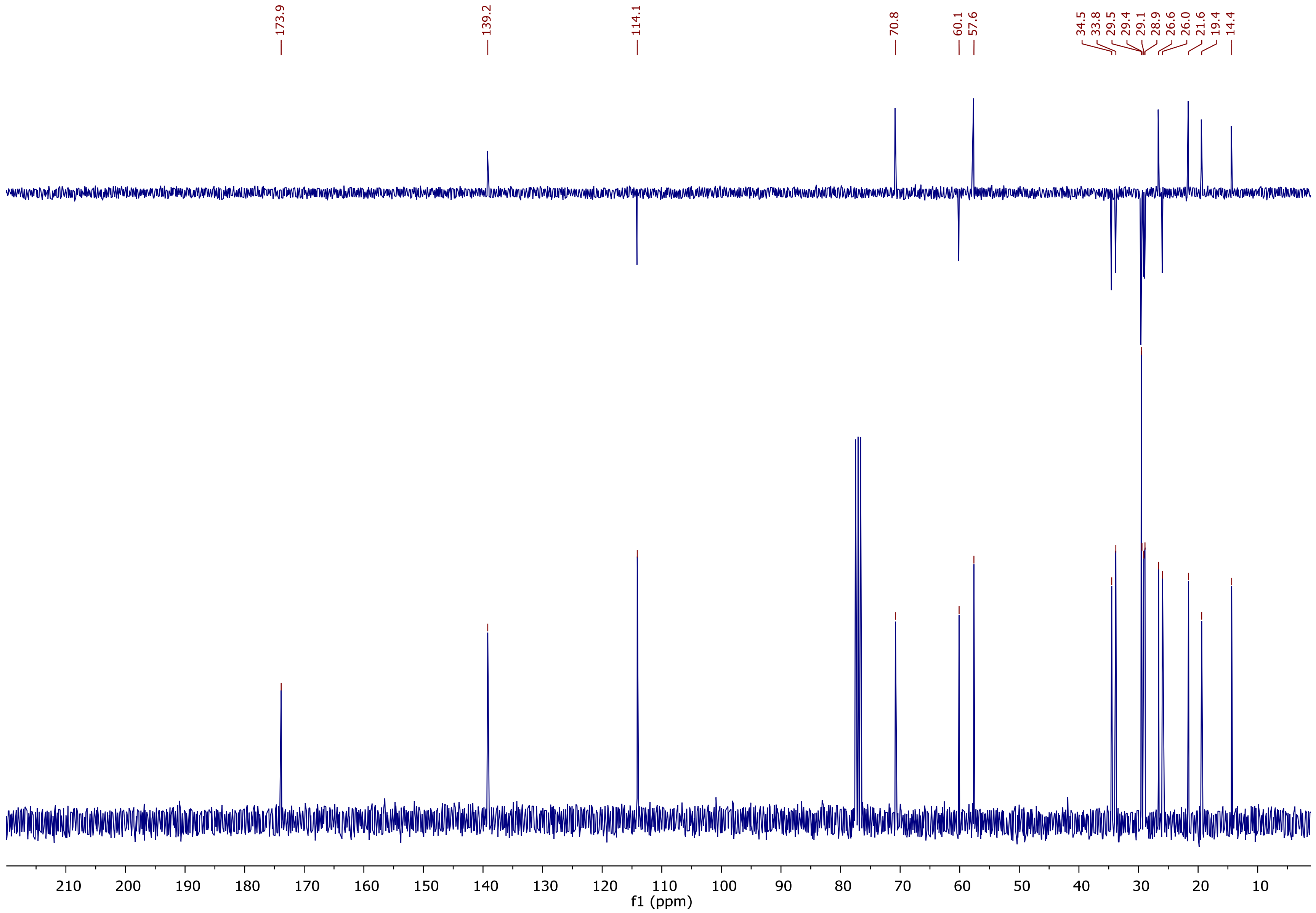

173.9
139.2
114.1
70.8
60.1
57.6
34.5
33.8
29.5
29.4
29.1
28.9
26.6
26.0
21.6
19.4
14.4
210 200 190 180 170 160 150 140 130 120 110 100 90 80 70 60 50 40 30 20 10
f1 (ppm)

Figure S14

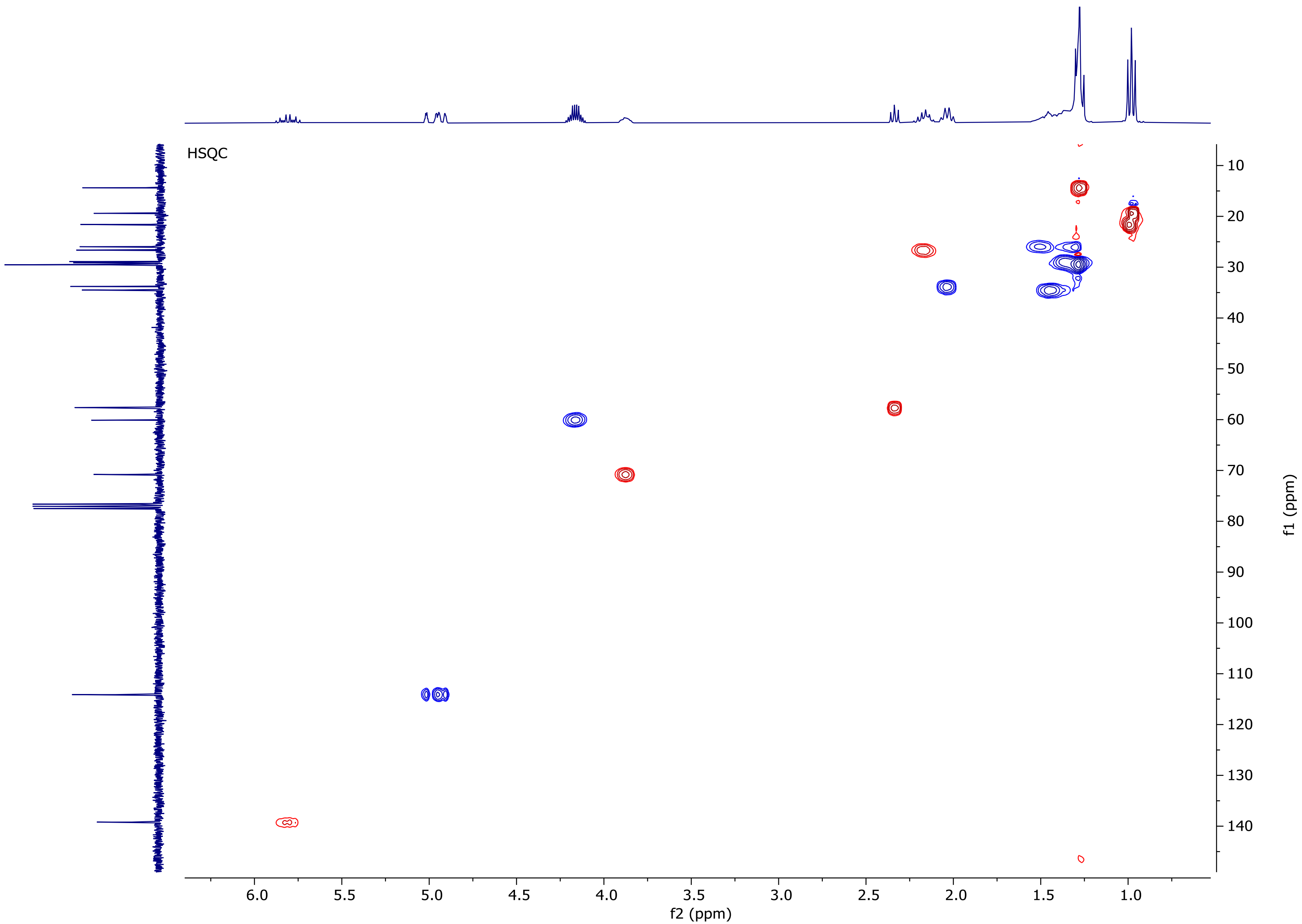

HSQC
6.0
5.5
5.0
4.5
4.0
3.5
3.0
2.5
2.0
1.5
1.0
f2 (ppm)
10
20
30
40
50
60
70
80
90
100
110
120
130
140
f1 (ppm)

Figure S15

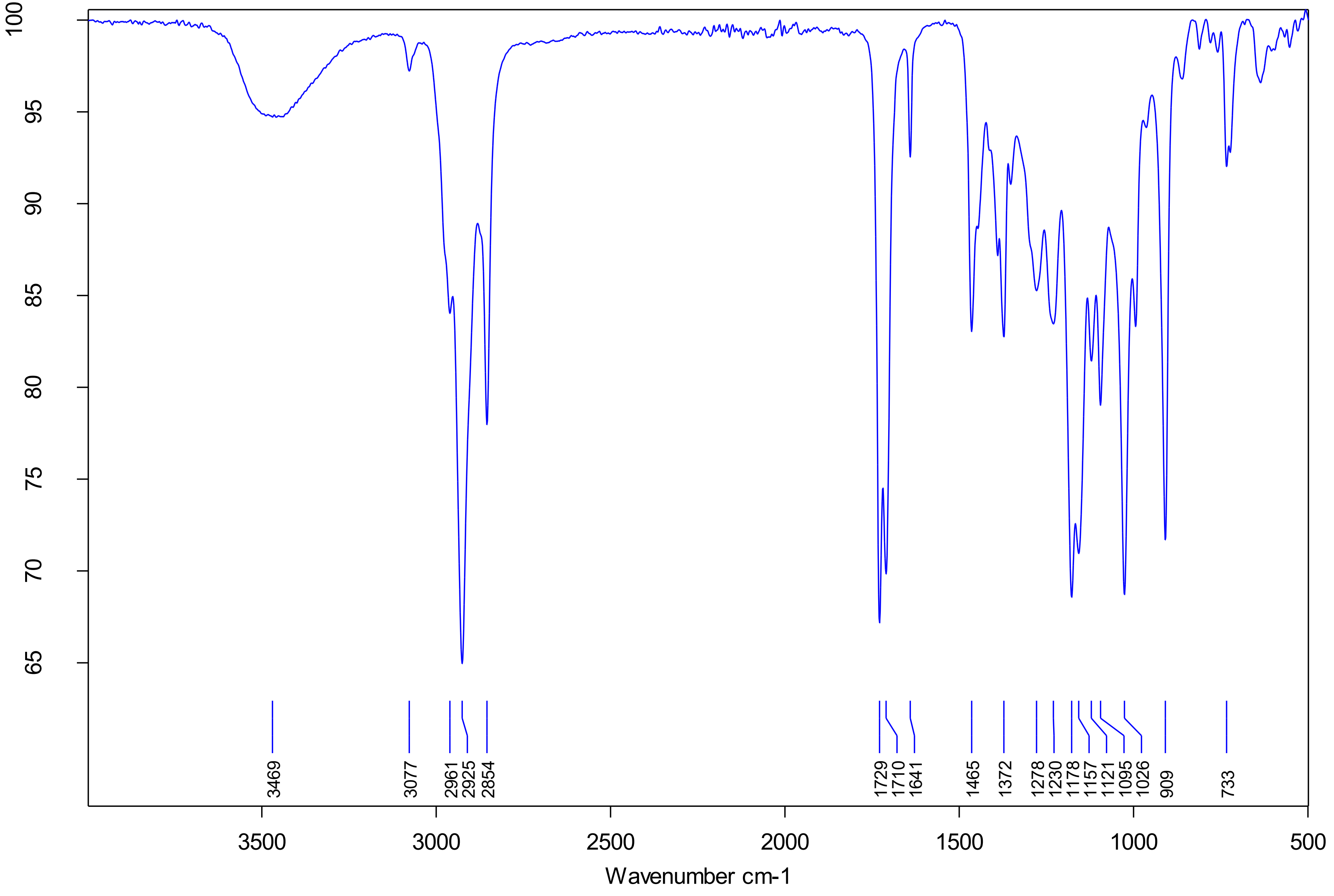

100
95
90
85
80
75
70
65
3469
3077
2961
2925
2854
1729
1710
1641
1465
1372
1278
1230
1178
1157
1121
1095
1026
909
733
3500
3000
2500
2000
1500
1000
500
Wavenumber cm-1

Figure S16

^{1}H NMR, DEPT 135, ^{13}C NMR, HSQC and IR of 5d *anti*

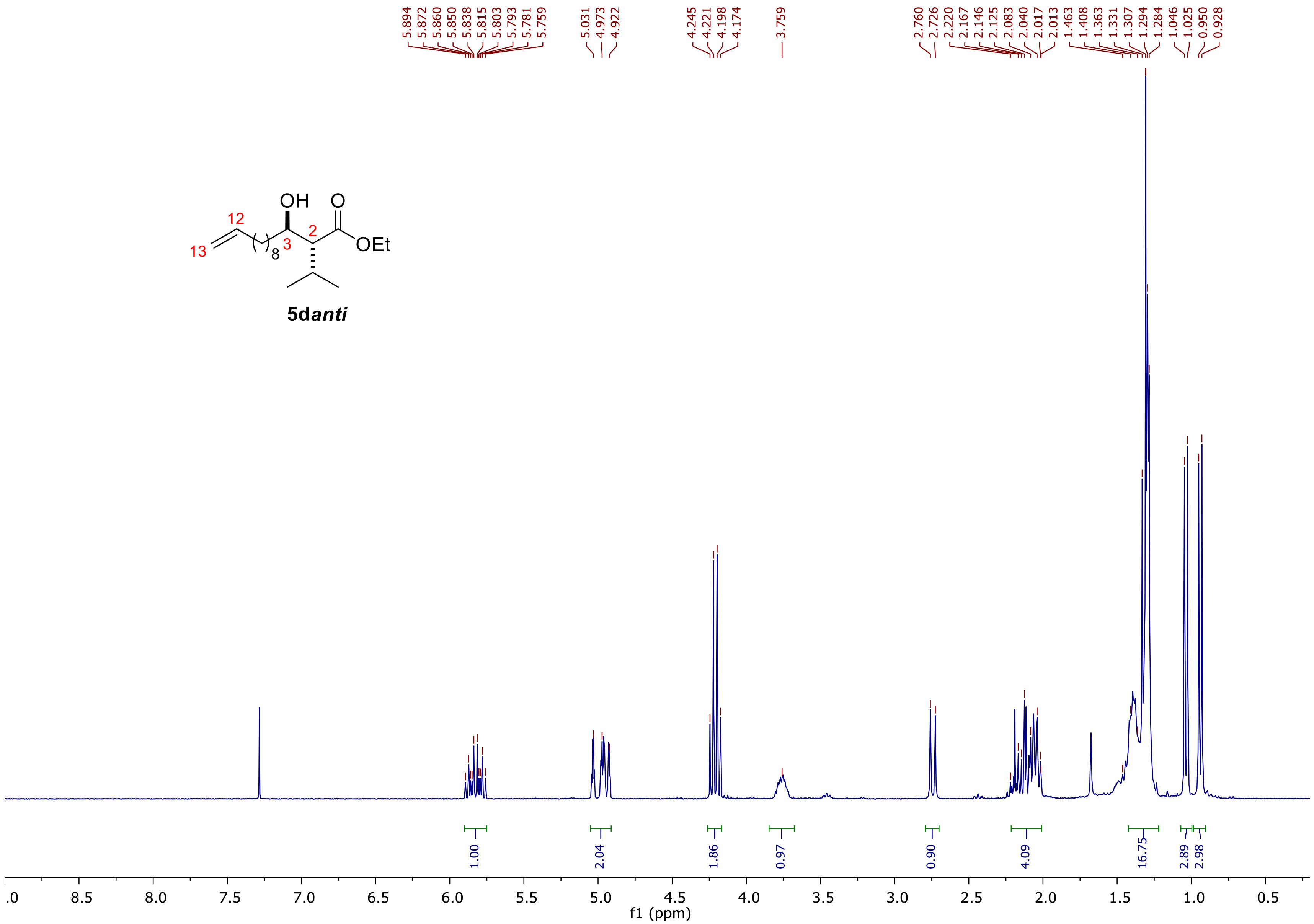

Figure S17

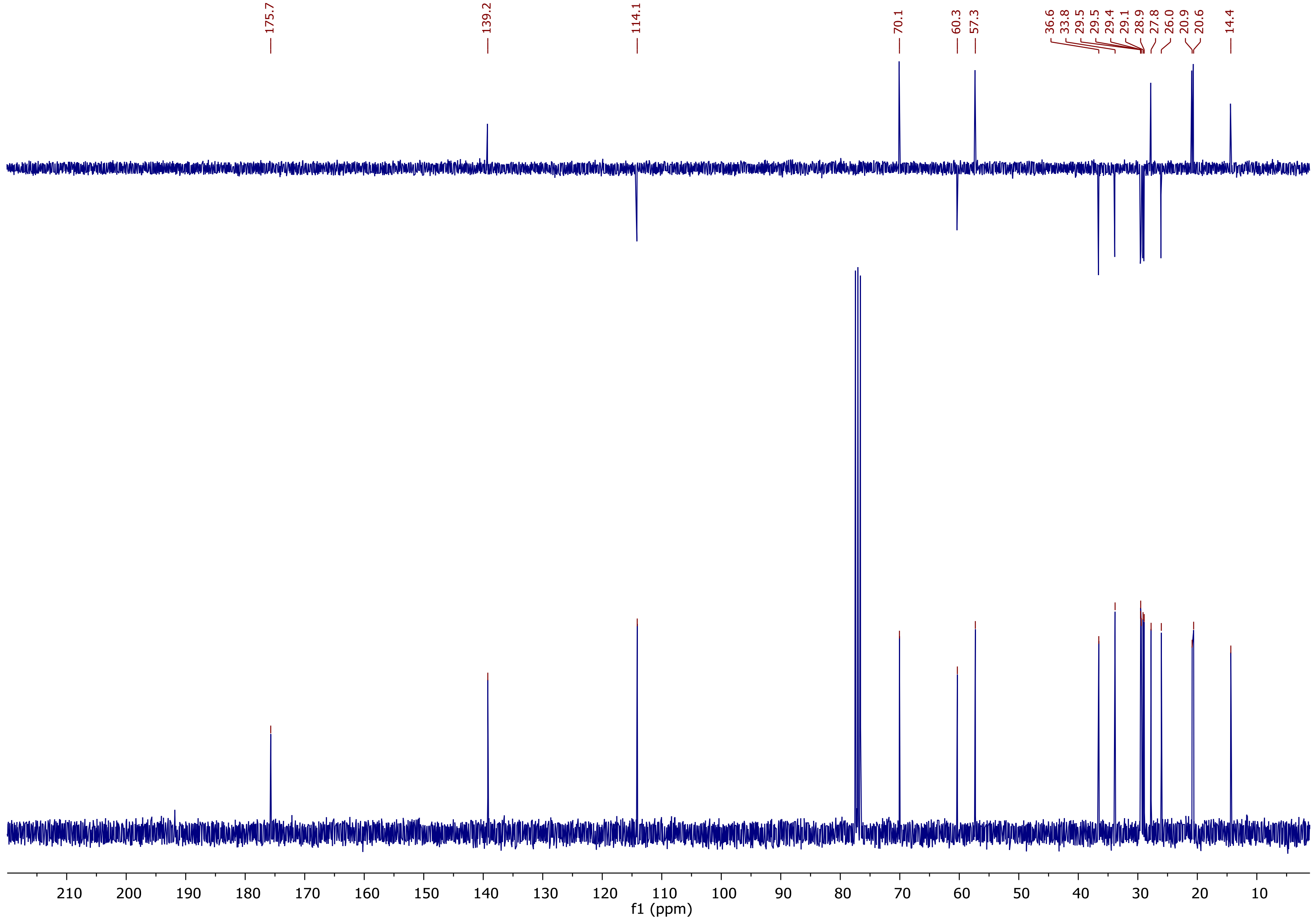
175.7
139.2
114.1
70.1
60.3
57.3
36.6
33.8
29.5
29.5
29.4
29.1
28.9
27.8
26.0
20.9
20.6
14.4
210 200 190 180 170 160 150 140 130 120 110 100 90 80 70 60 50 40 30 20 10
f1 (ppm)

Figure S18

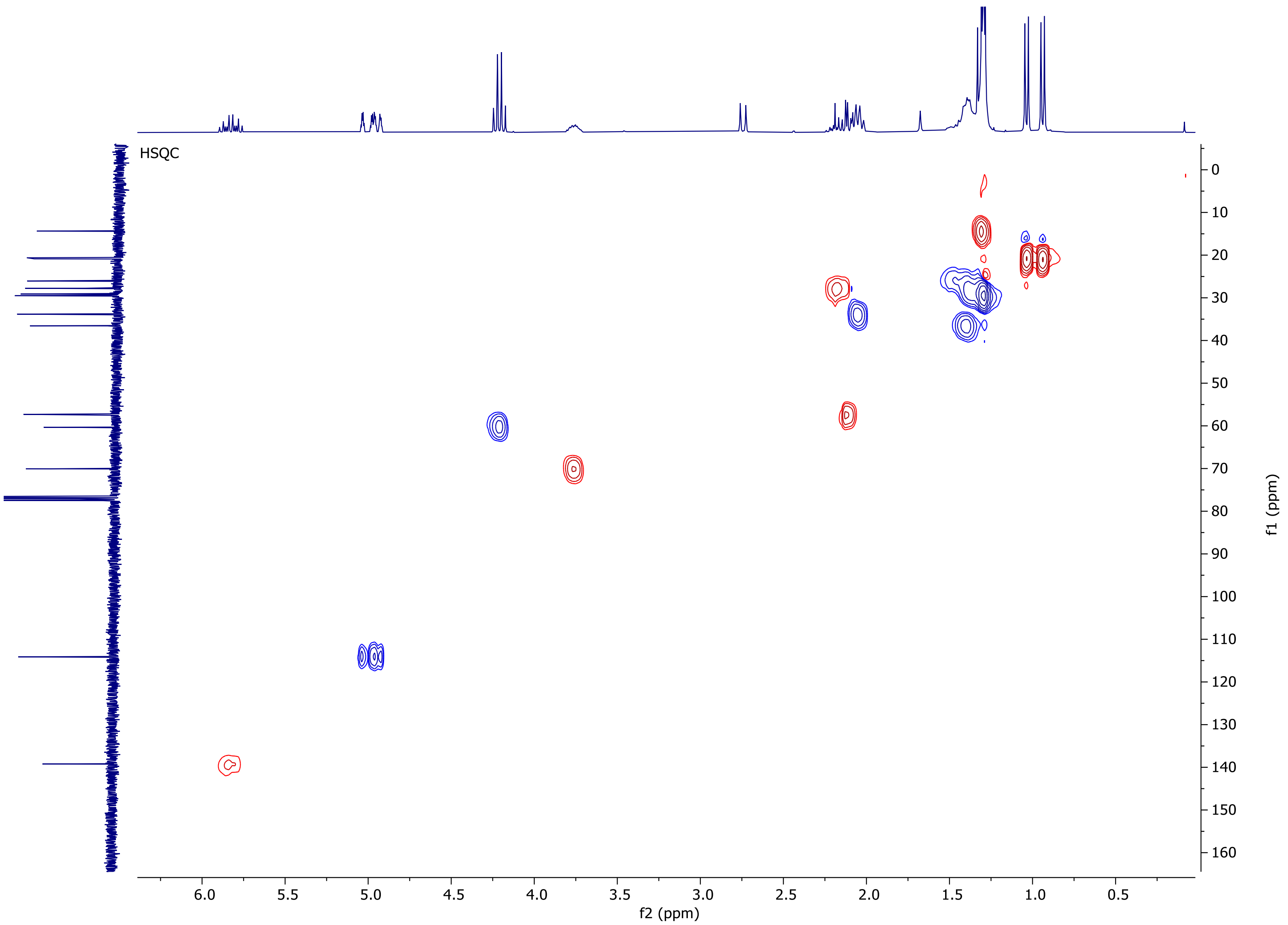
HSQC
f1 (ppm)
f2 (ppm)
0
10
20
30
40
50
60
70
80
90
100
110
120
130
140
150
160
6.0
5.5
5.0
4.5
4.0
3.5
3.0
2.5
2.0
1.5
1.0
0.5

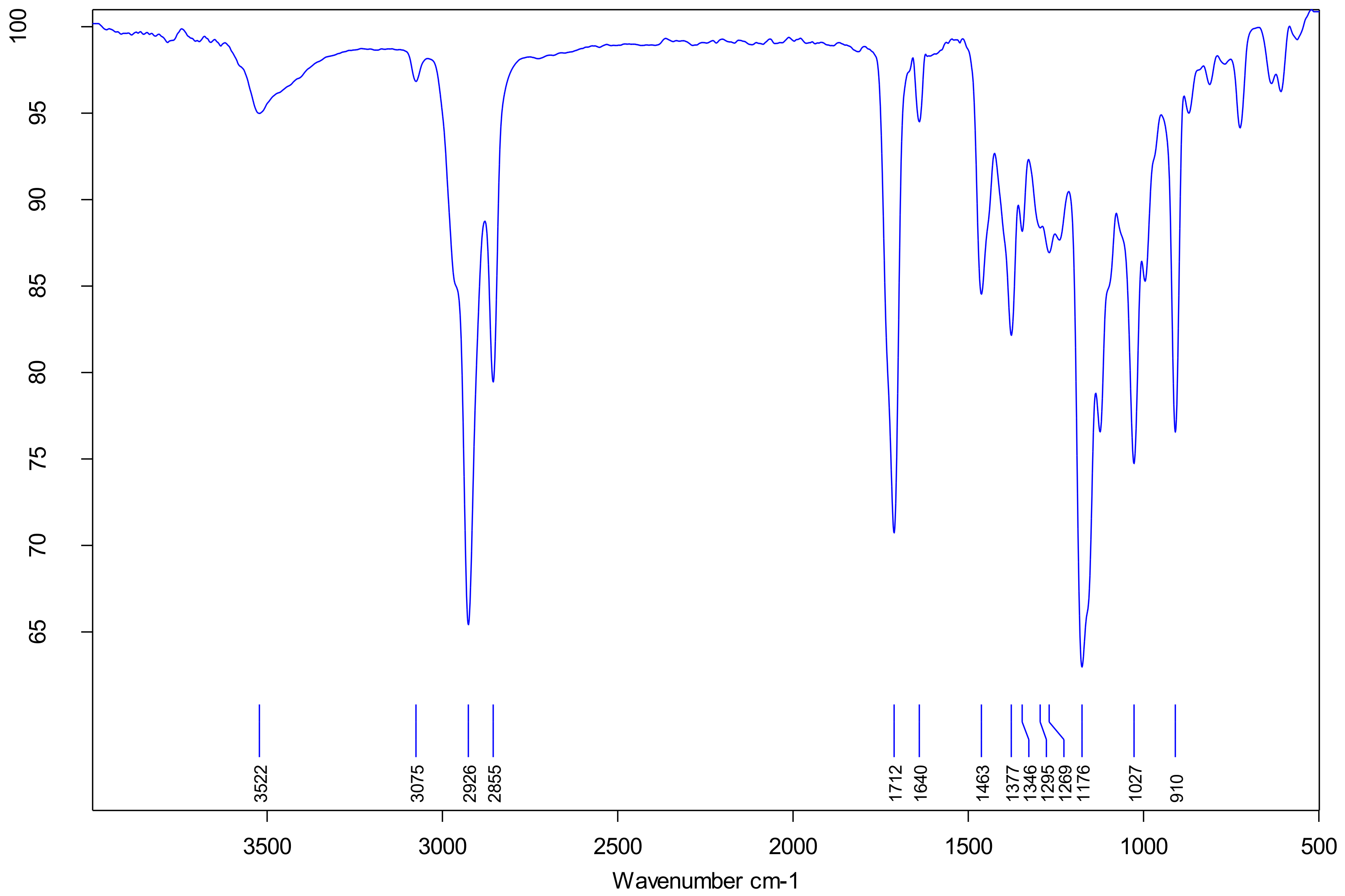
100
95
90
85
80
75
70
65
3522
3075
2926
2855
1712
1640
1463
1377
1346
1295
1269
1176
1027
910
3500
3000
2500
2000
1500
1000
500
Wavenumber cm-1

Figure S20

^{1}H NMR, DEPT 135, ^{13}C NMR, HSQC, NOESY 1D and IR of 5e *syn*

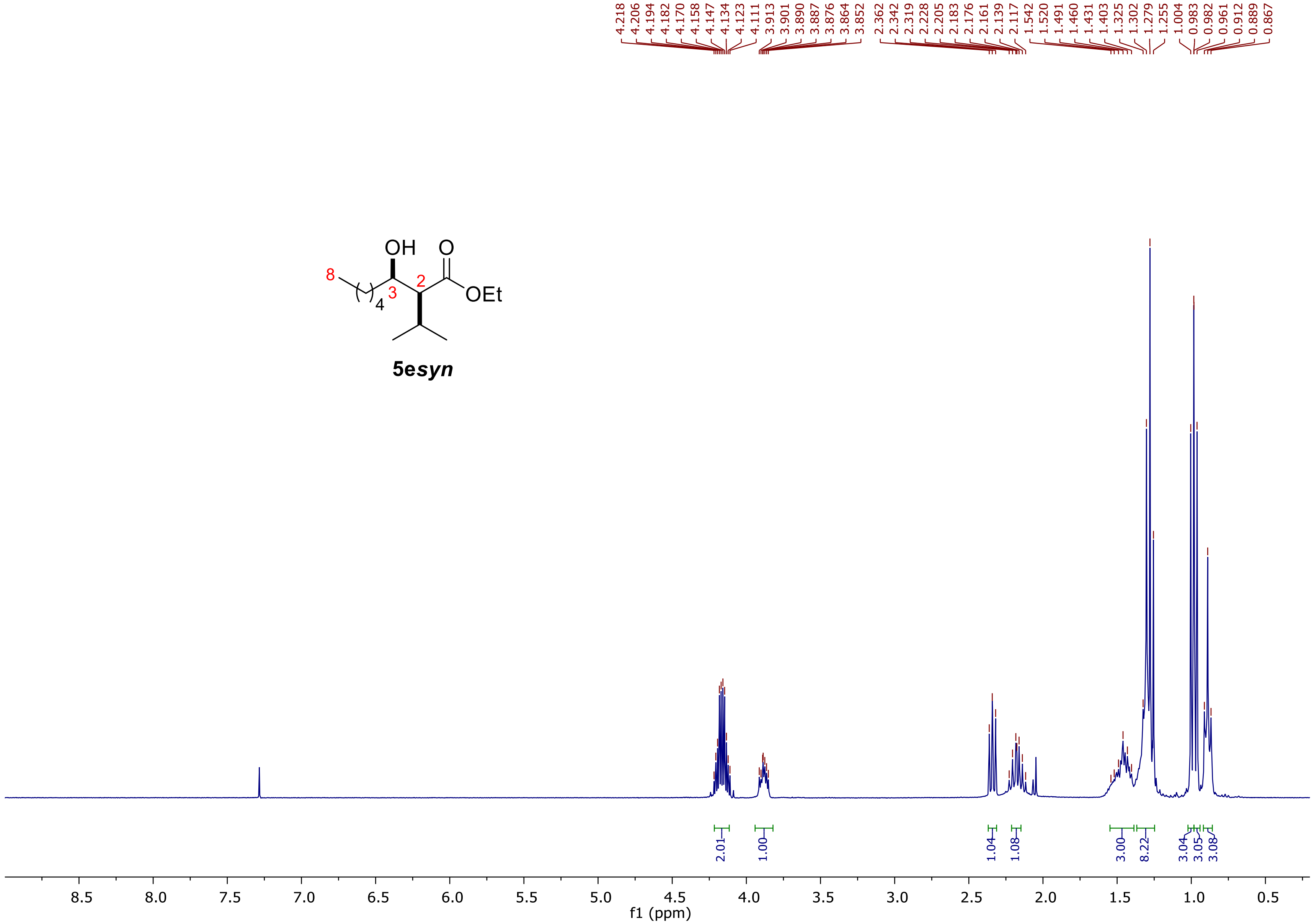

Figure S21

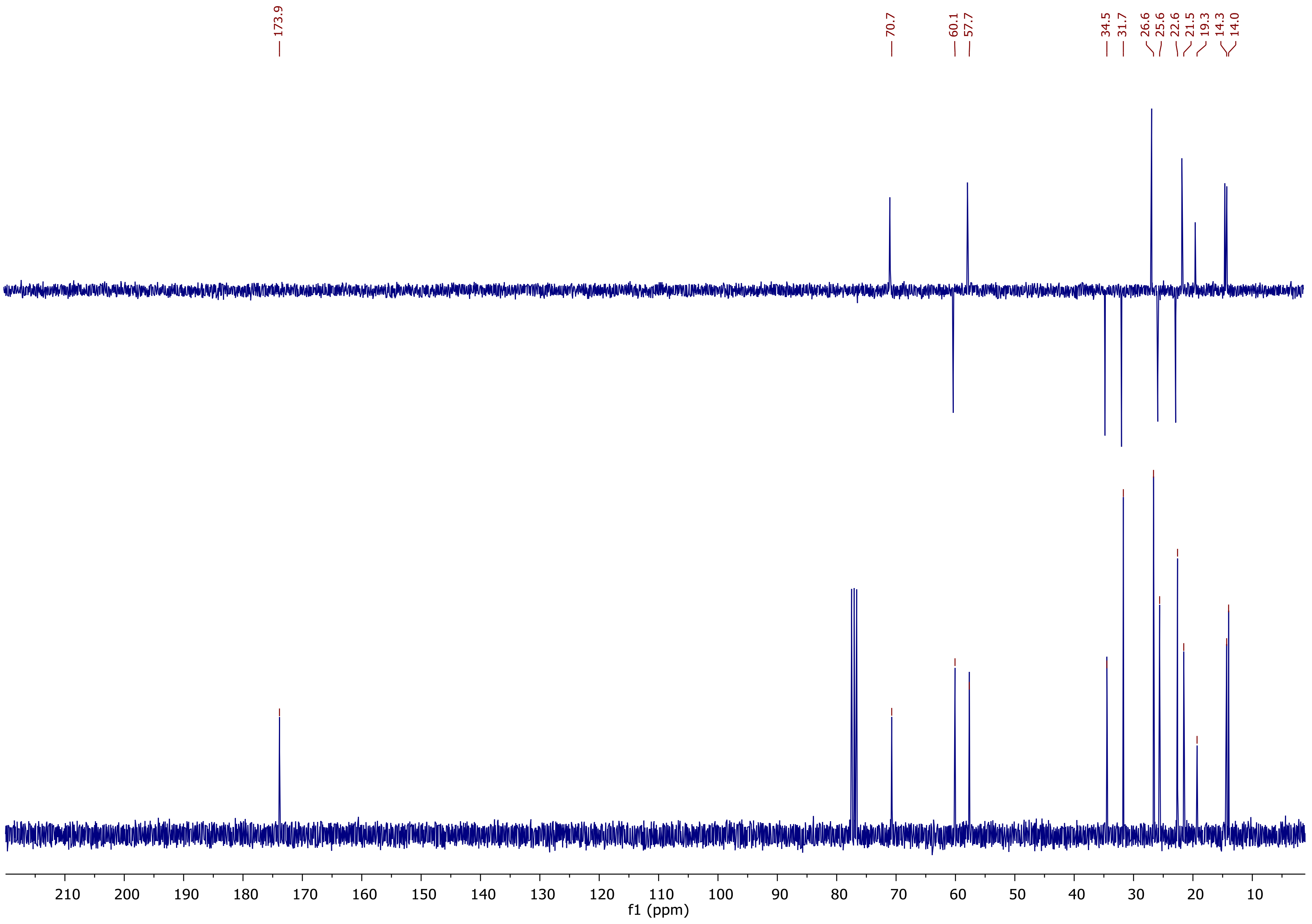

173.9
70.7
60.1
57.7
34.5
31.7
26.6
25.6
22.6
21.5
19.3
14.3
14.0
210 200 190 180 170 160 150 140 130 120 110 100 90 80 70 60 50 40 30 20 10
f1 (ppm)

Figure S22

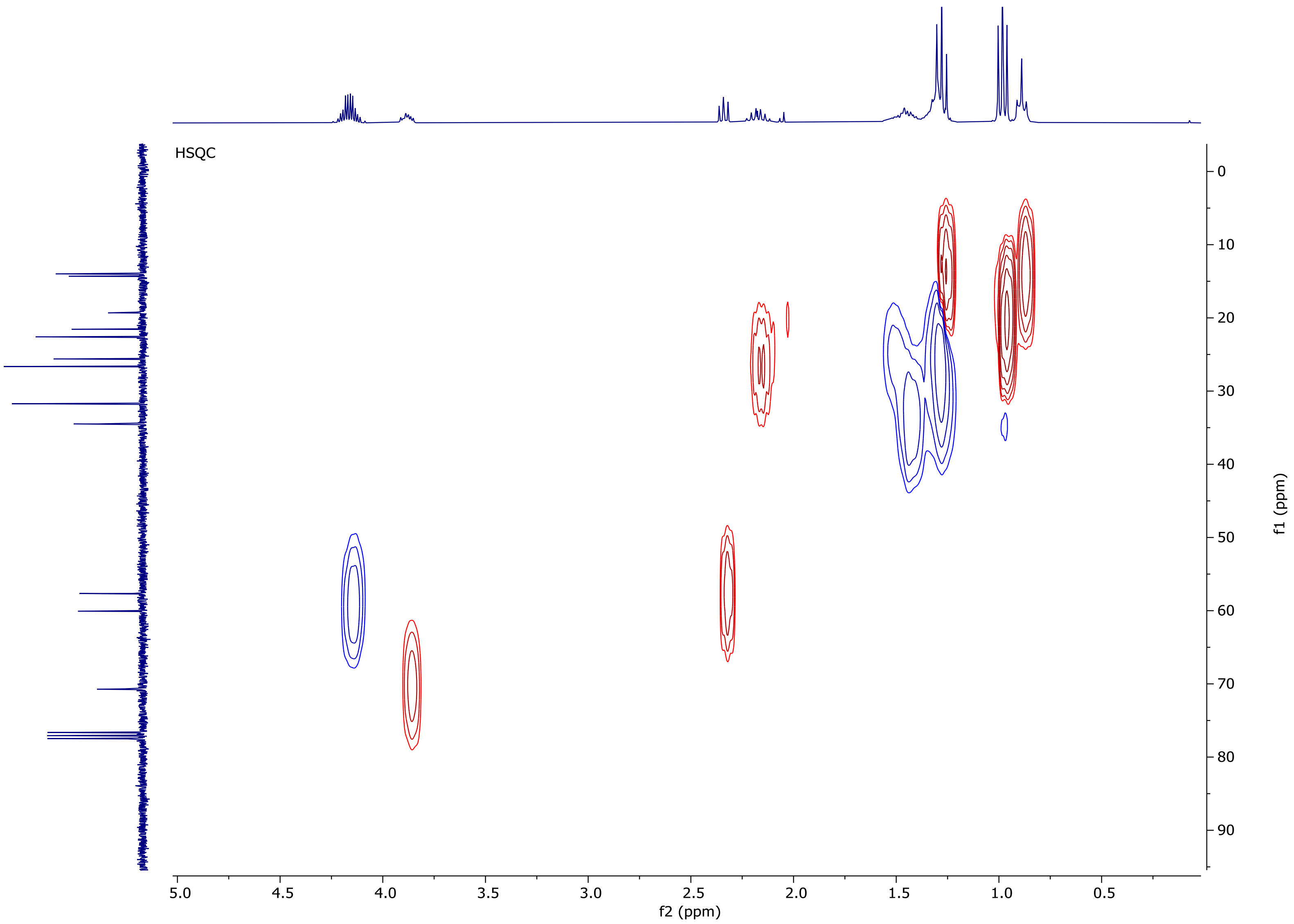
HSQC
5.0
4.5
4.0
3.5
3.0
2.5
2.0
1.5
1.0
0.5
f2 (ppm)
0
10
20
30
40
50
60
70
80
90
f1 (ppm)

Figure S23

1D Selective Gradient NOESY — freq: 3.877ppm

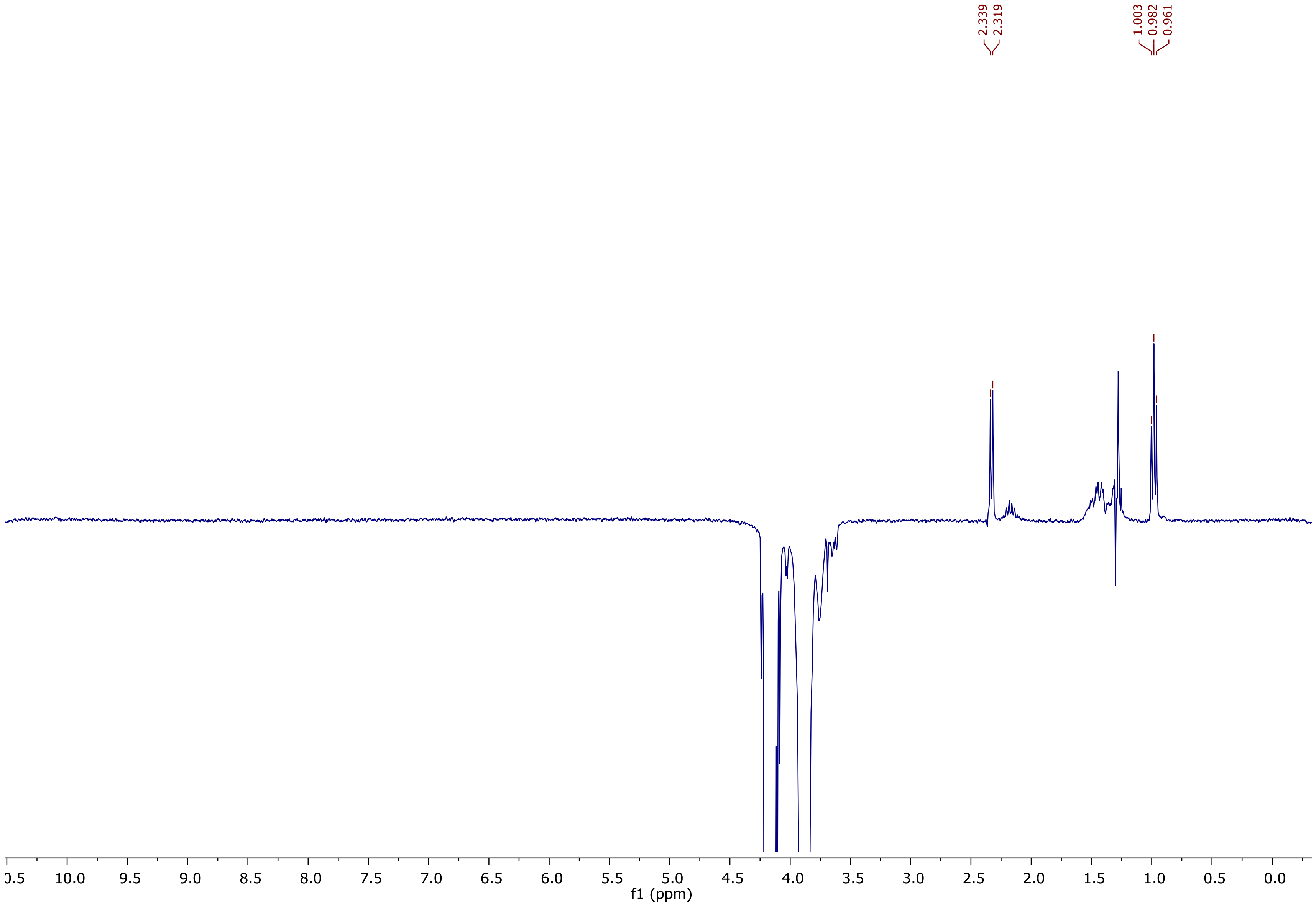

Figure S24

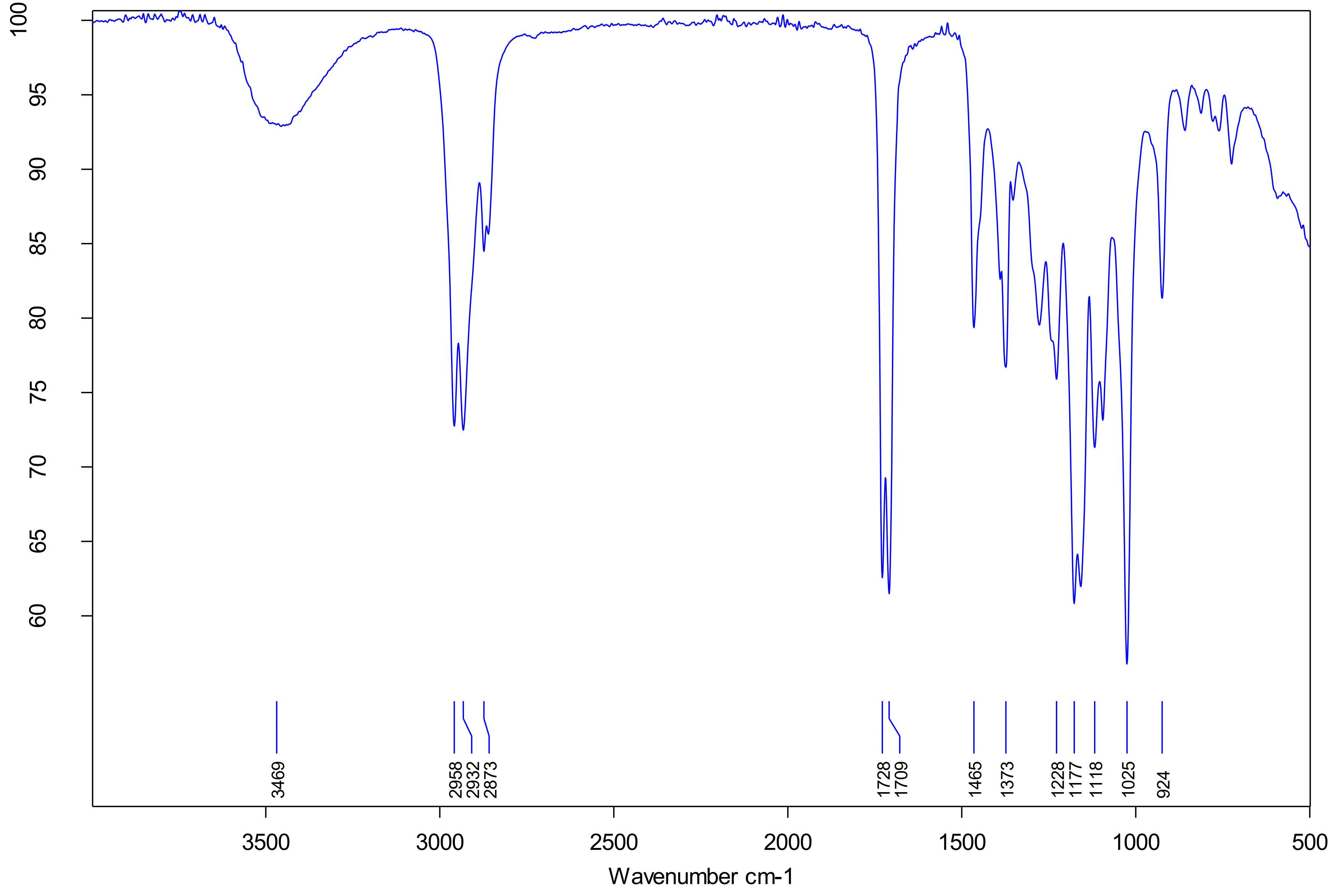
100
95
90
85
80
75
70
65
60
3469
2958
2932
2873
1728
1709
1465
1373
1228
1177
1118
1025
924
3500
3000
2500
2000
1500
1000
500
Wavenumber cm-1

Figure S25

^{1}H NMR, DEPT 135, ^{13}C NMR, HSQC, NOESY 1D and IR of 5e *anti*

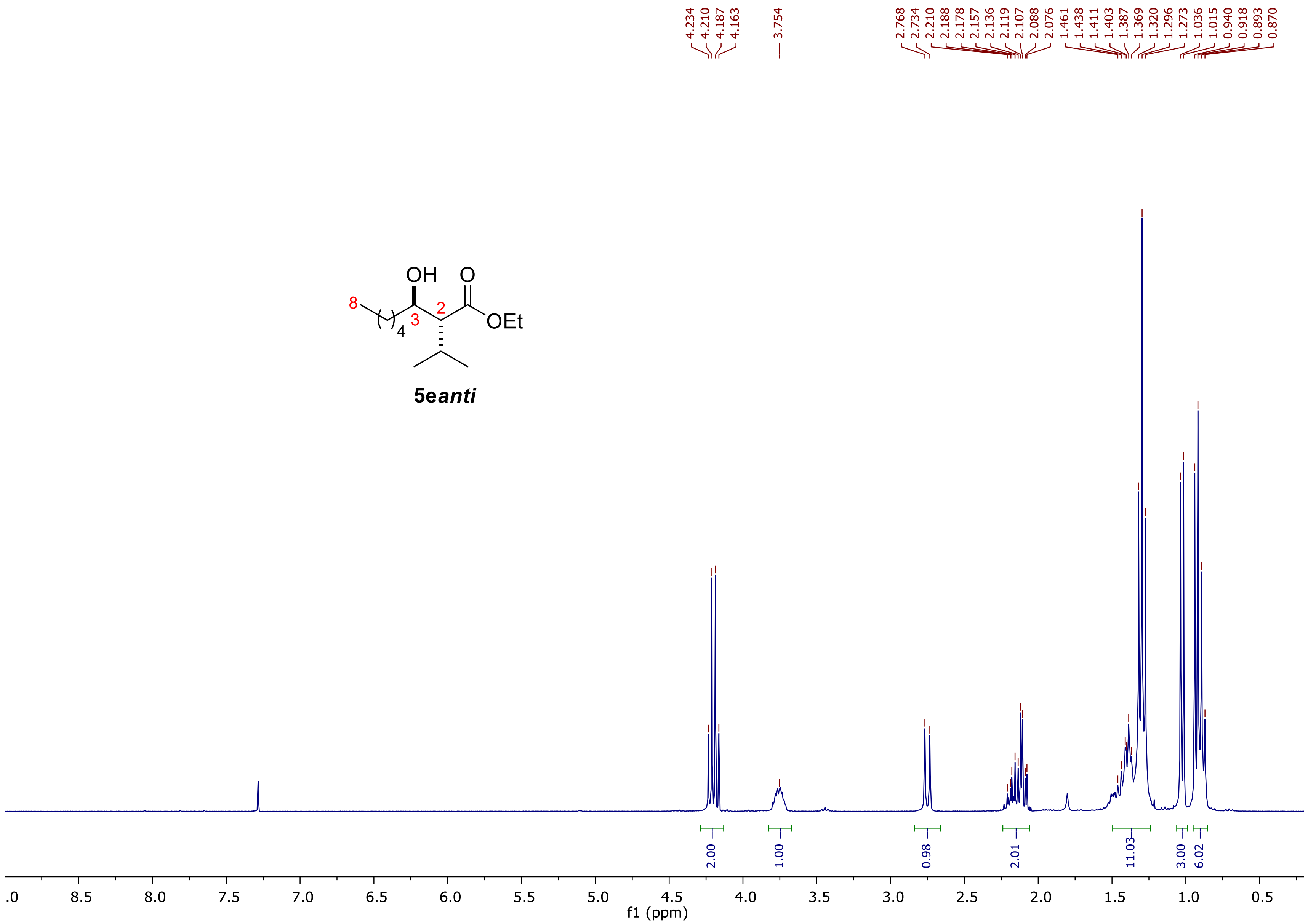

Figure S26

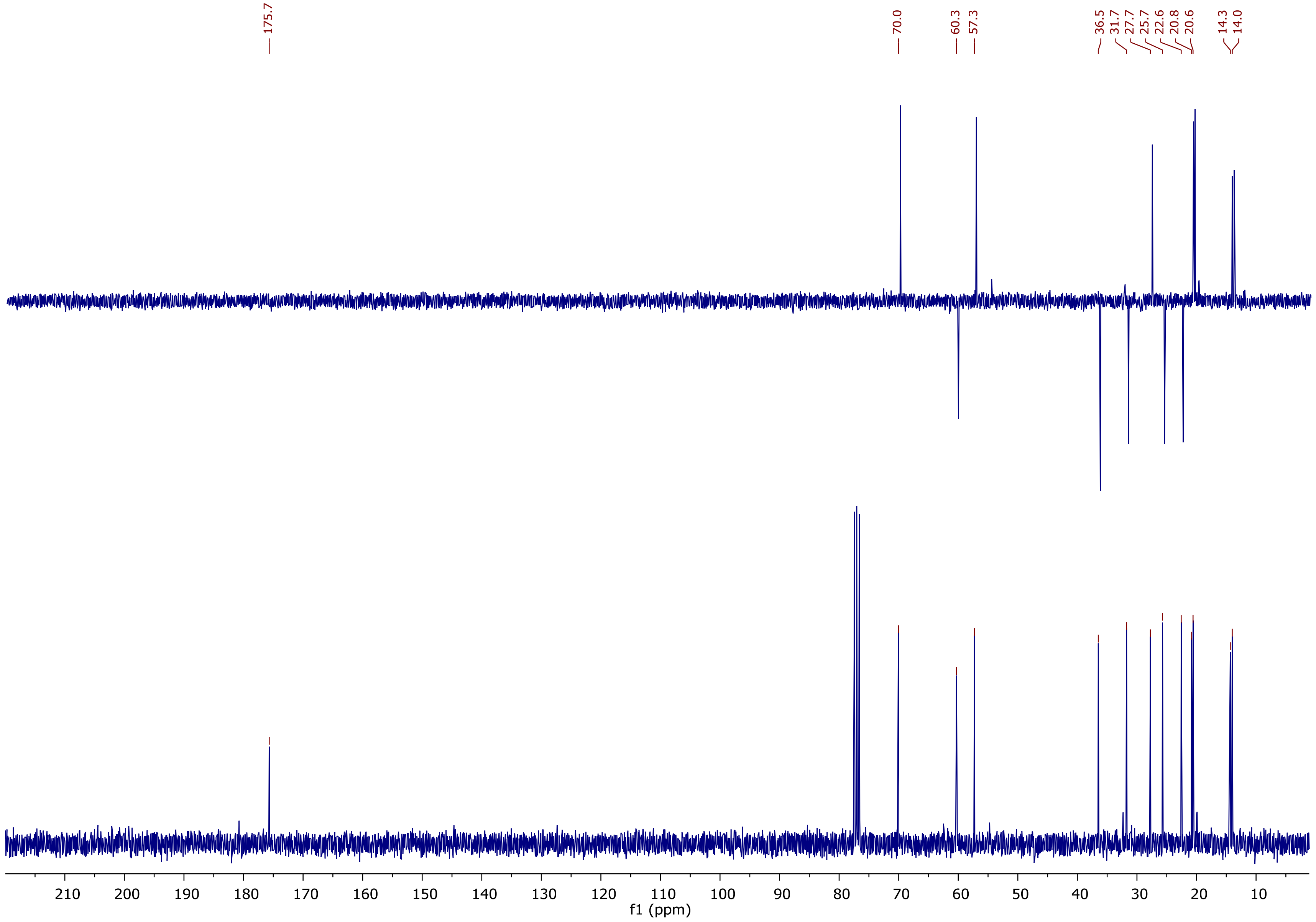

175.7
70.0
60.3
57.3
36.5
31.7
27.7
25.7
22.6
20.8
20.6
14.3
14.0
210
200
190
180
170
160
150
140
130
120
110
100
90
80
70
60
50
40
30
20
10
f1 (ppm)

Figure S27

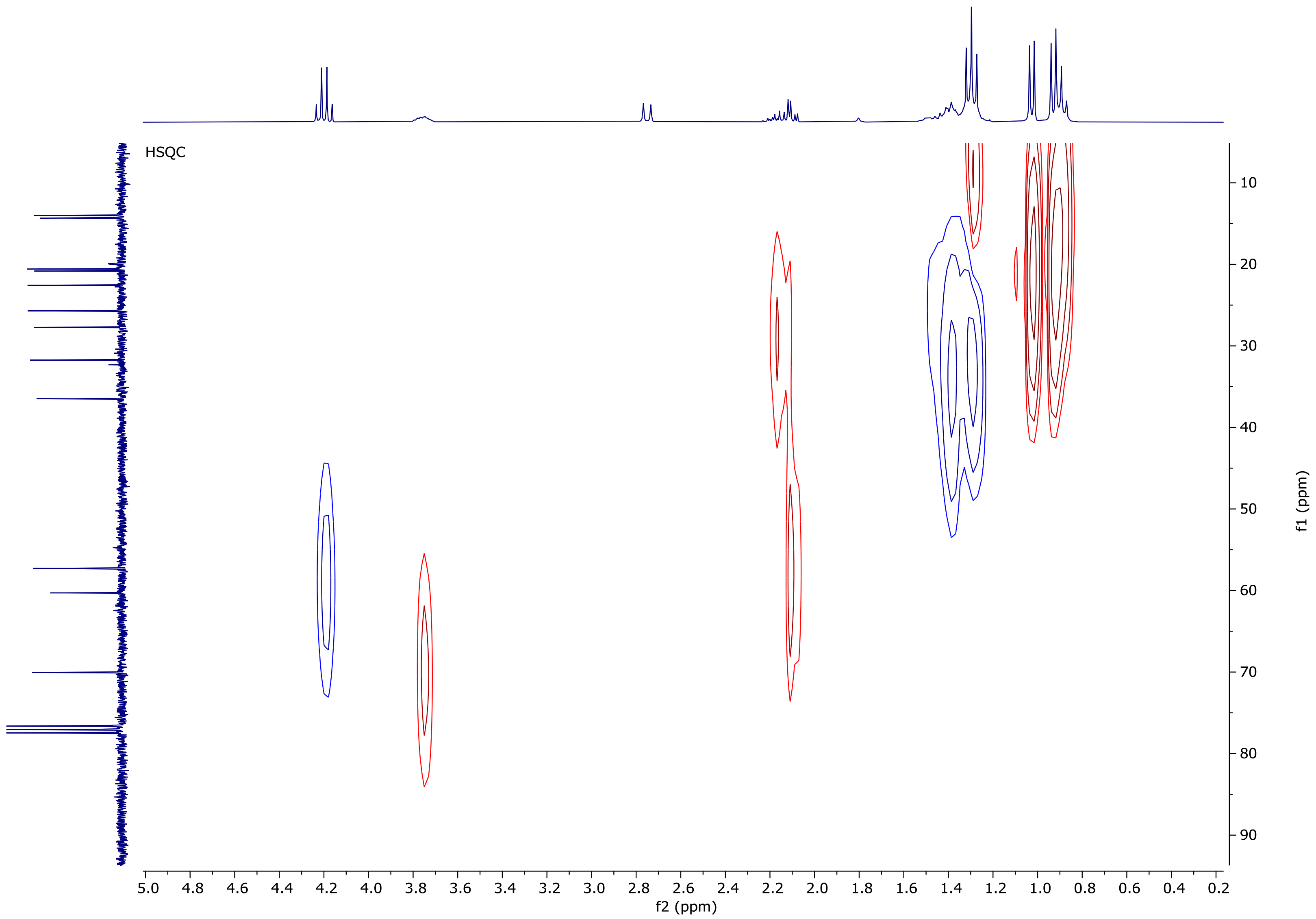
HSQC
5.0
4.8
4.6
4.4
4.2
4.0
3.8
3.6
3.4
3.2
3.0
2.8
2.6
2.4
2.2
2.0
1.8
1.6
1.4
1.2
1.0
0.8
0.6
0.4
0.2
f2 (ppm)
10
20
30
40
50
60
70
80
90
f1 (ppm)

Figure S28

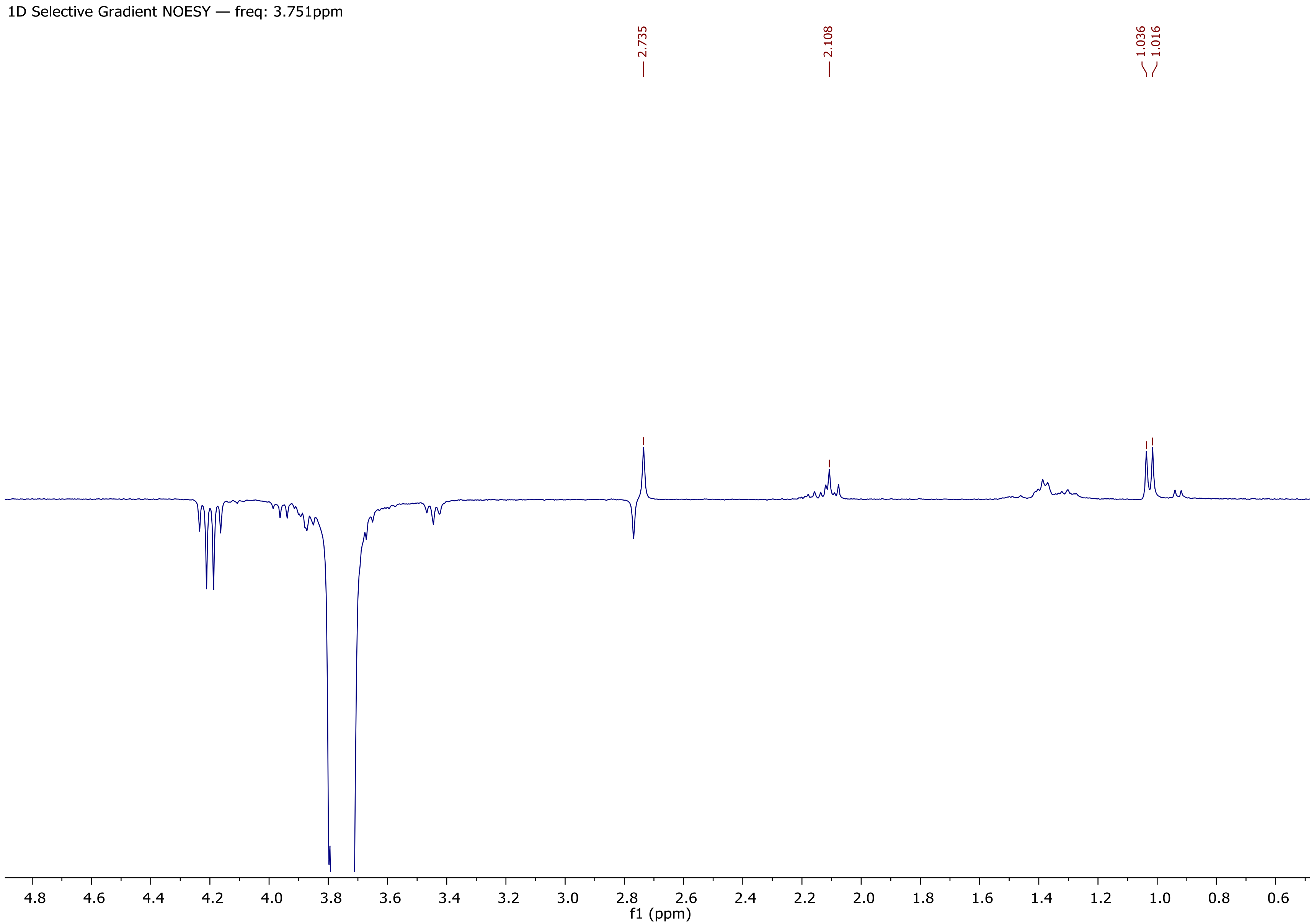

1D Selective Gradient NOESY — freq: 3.751ppm
2.735
2.108
1.036
1.016
4.8
4.6
4.4
4.2
4.0
3.8
3.6
3.4
3.2
3.0
2.8
2.6
2.4
2.2
2.0
1.8
1.6
1.4
1.2
1.0
0.8
0.6
f1 (ppm)

Figure S29

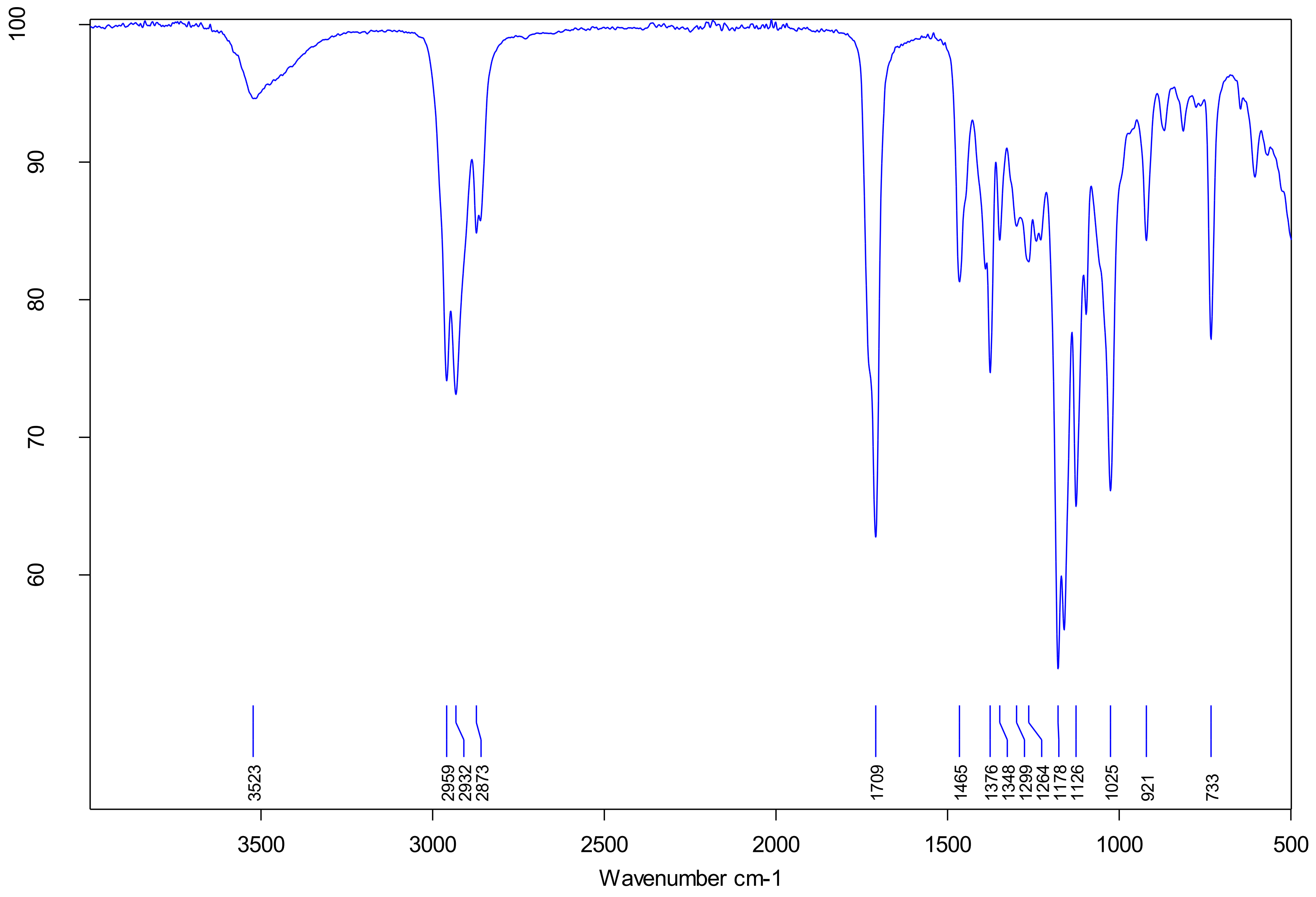

100
90
80
70
60
3523
2959
2932
2873
1709
1465
1376
1348
1299
1264
1178
1126
1025
921
733
3500
3000
2500
2000
1500
1000
500
Wavenumber cm-1

Figure S30

^{1}H NMR, DEPT 135, ^{13}C NMR, HSQC, NOESY 1D and IR of 5f

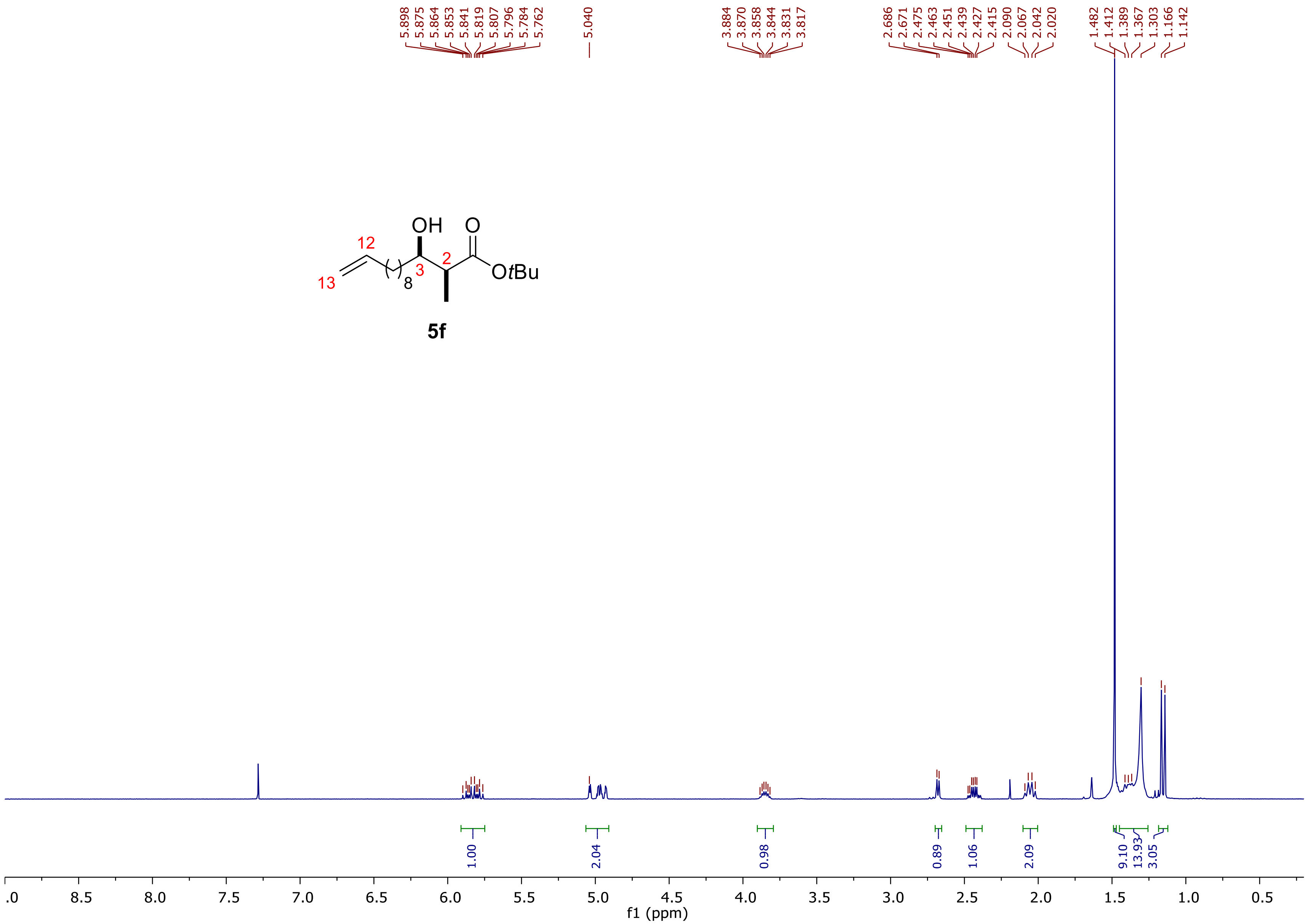

Figure S31

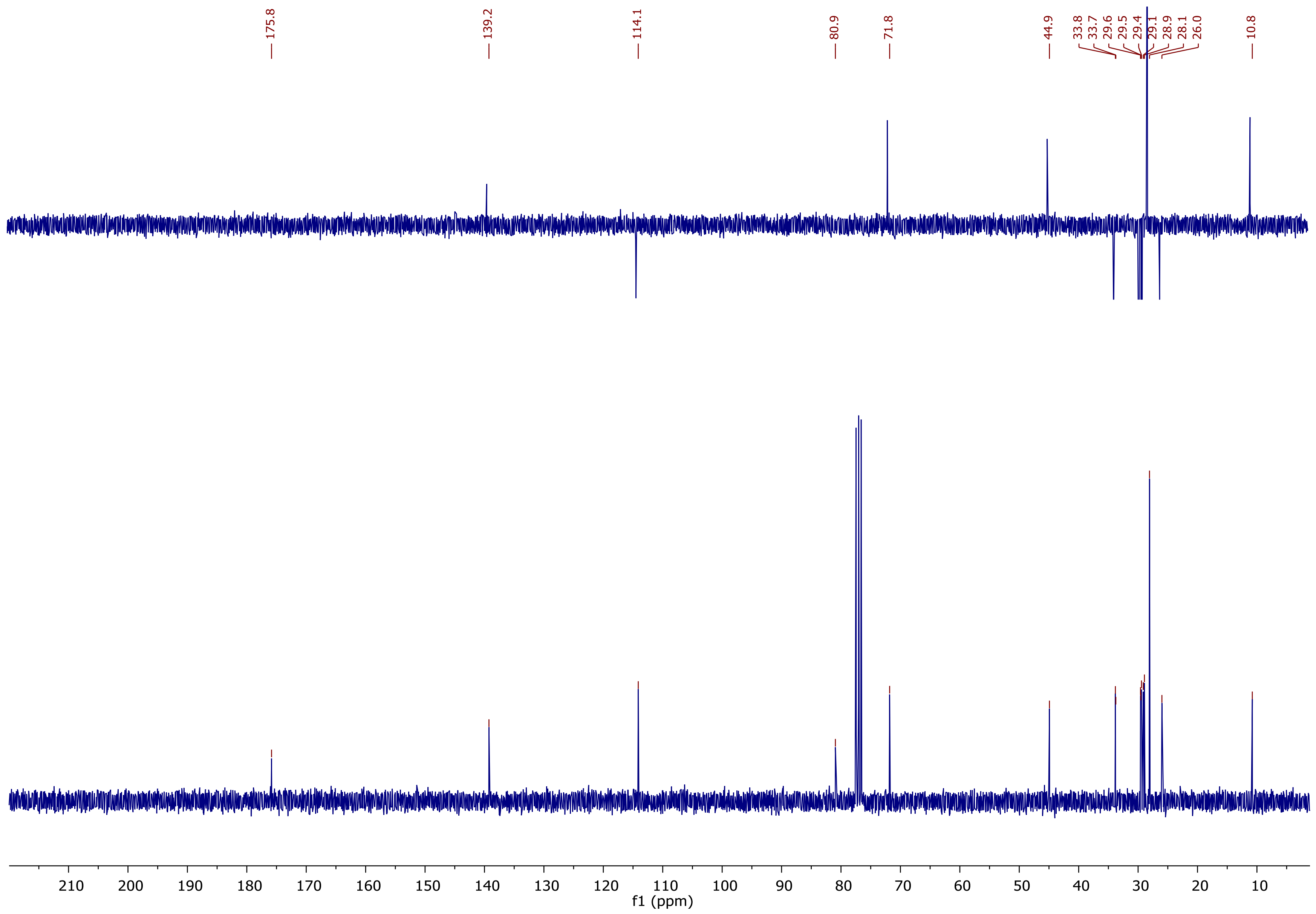
175.8
139.2
114.1
80.9
71.8
44.9
33.8
33.7
29.6
29.5
29.4
29.1
28.9
28.1
26.0
10.8
210 200 190 180 170 160 150 140 130 120 110 100 90 80 70 60 50 40 30 20 10
f1 (ppm)

Figure S32

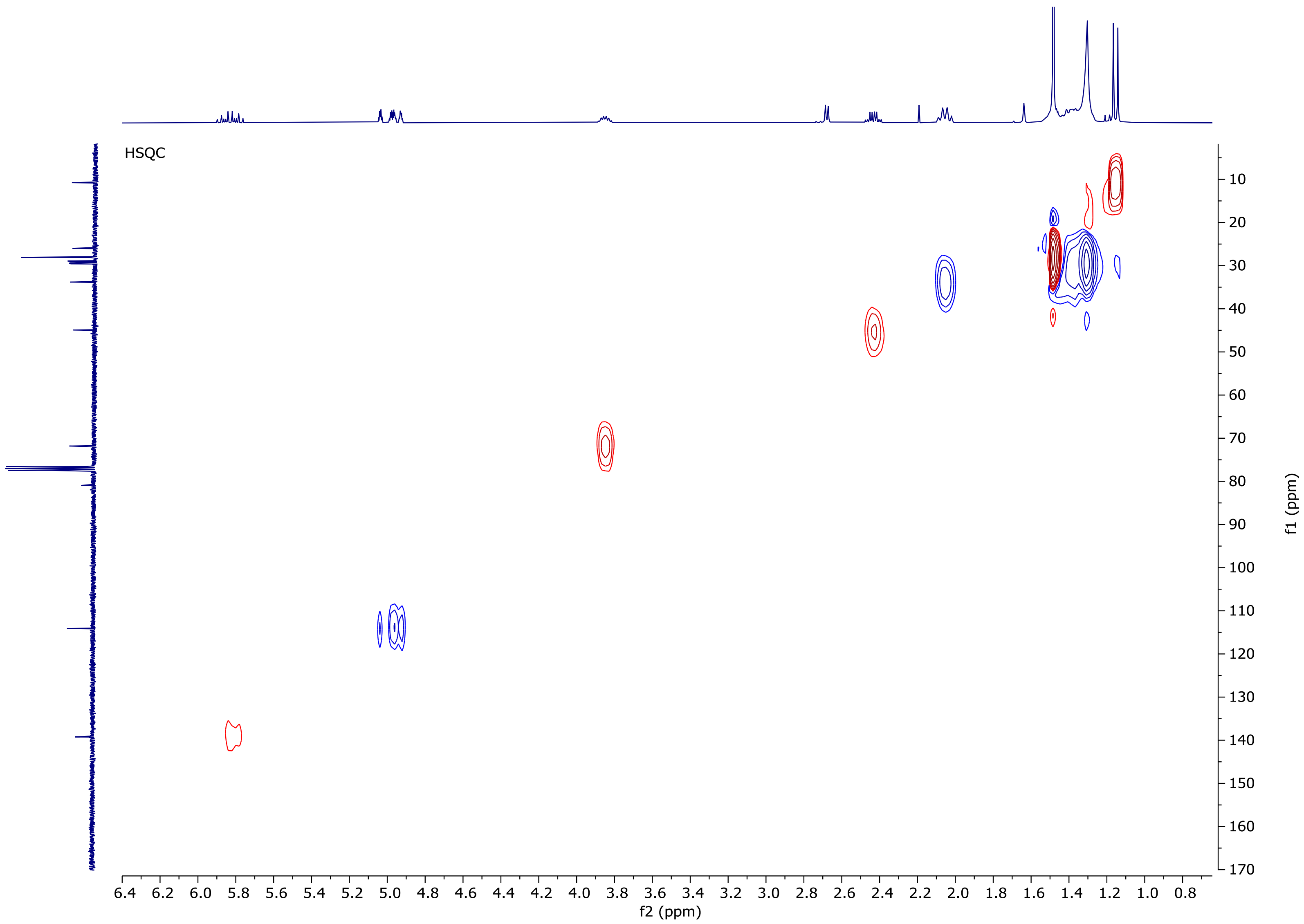
HSQC
6.4 6.2 6.0 5.8 5.6 5.4 5.2 5.0 4.8 4.6 4.4 4.2 4.0 3.8 3.6 3.4 3.2 3.0 2.8 2.6 2.4 2.2 2.0 1.8 1.6 1.4 1.2 1.0 0.8
f2 (ppm)
10 20 30 40 50 60 70 80 90 100 110 120 130 140 150 160 170
f1 (ppm)

Figure S33

1D Selective Gradient NOESY — freq: 3.853ppm

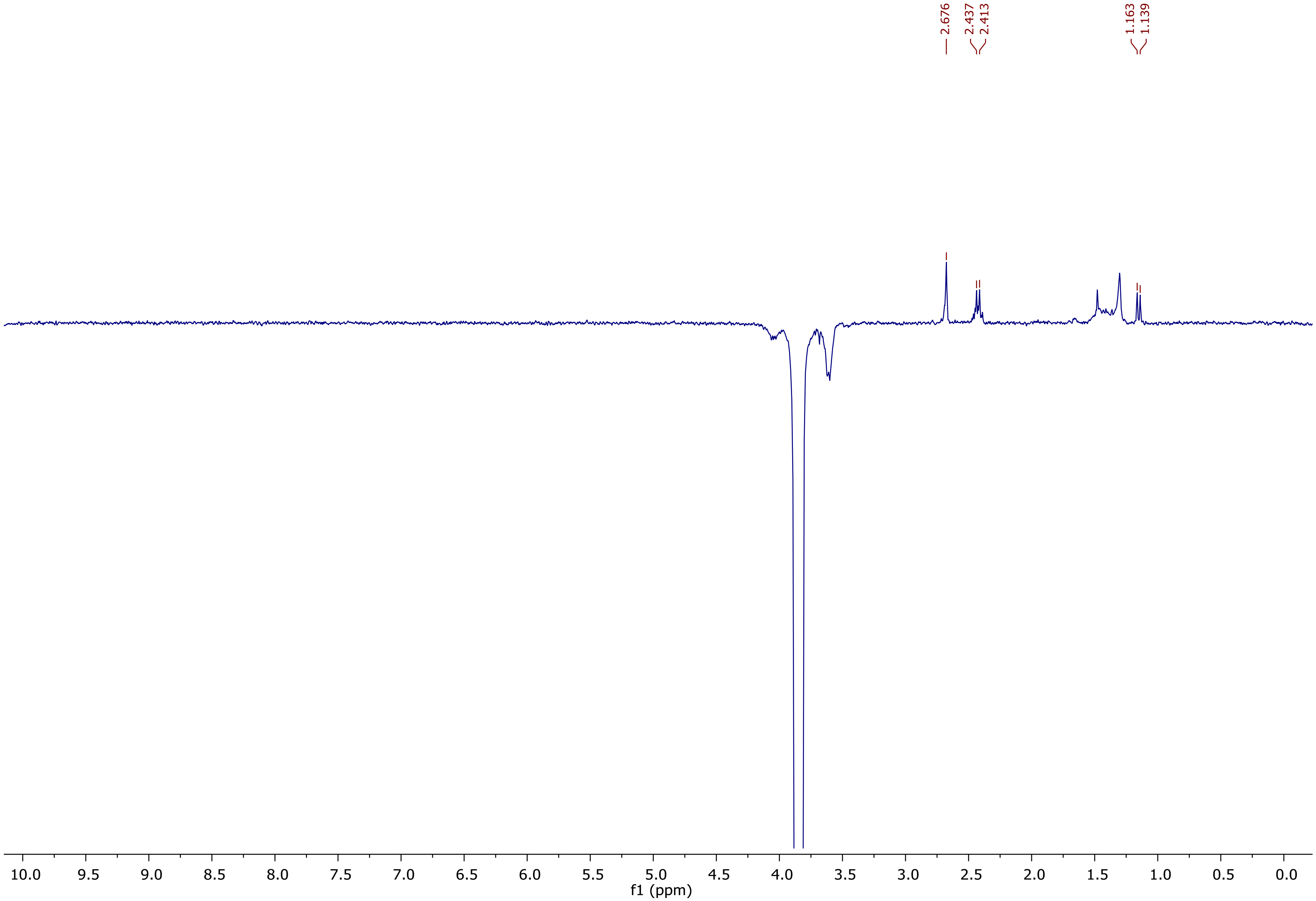

Figure S34

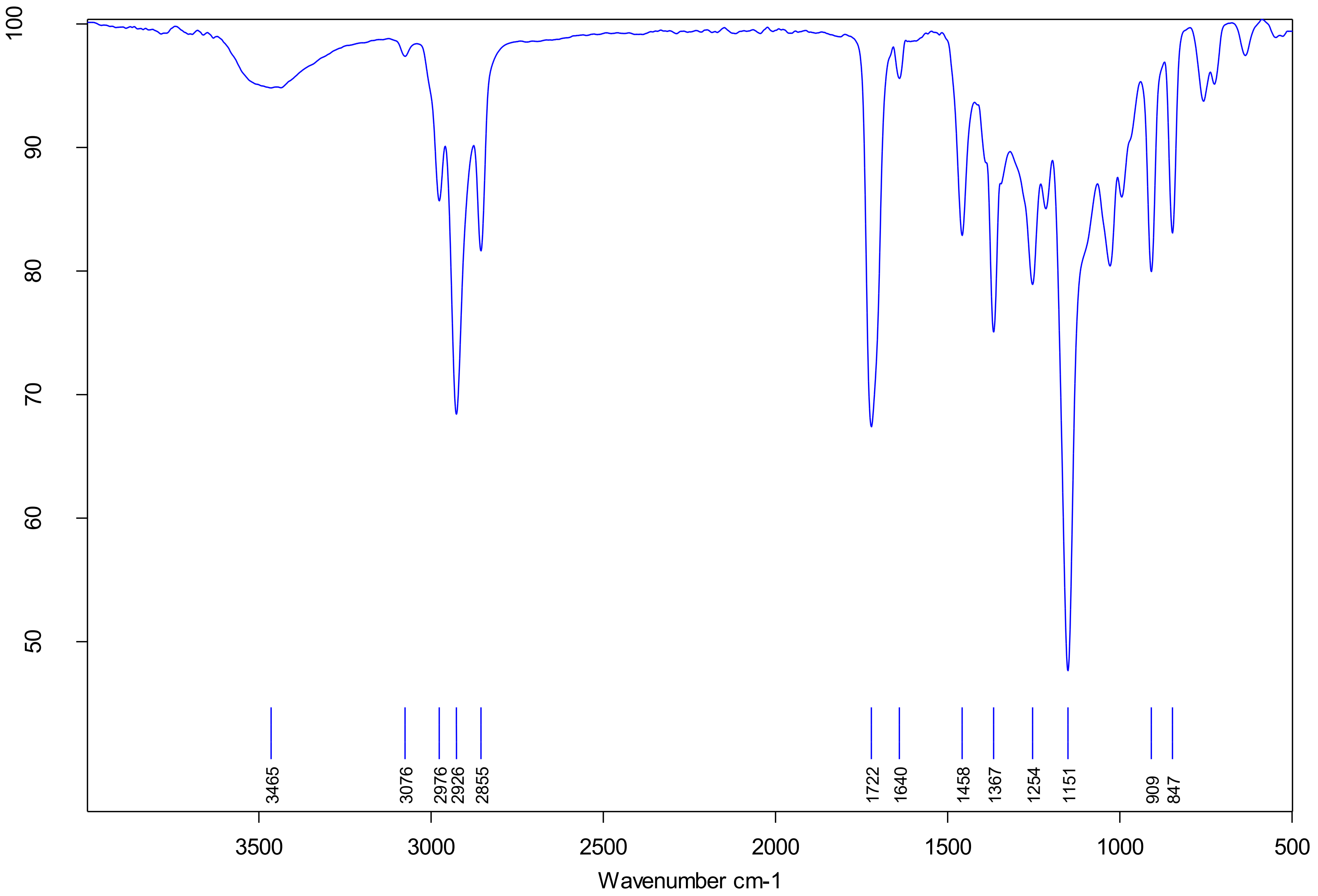

100
90
80
70
60
50
3465
3076
2976
2926
2855
1722
1640
1458
1367
1254
1151
909
847
3500
3000
2500
2000
1500
1000
500
Wavenumber cm-1

Figure S35

^{1}H NMR, DEPT 135, ^{13}C NMR, HSQC, NOESY 1D and IR of 5g

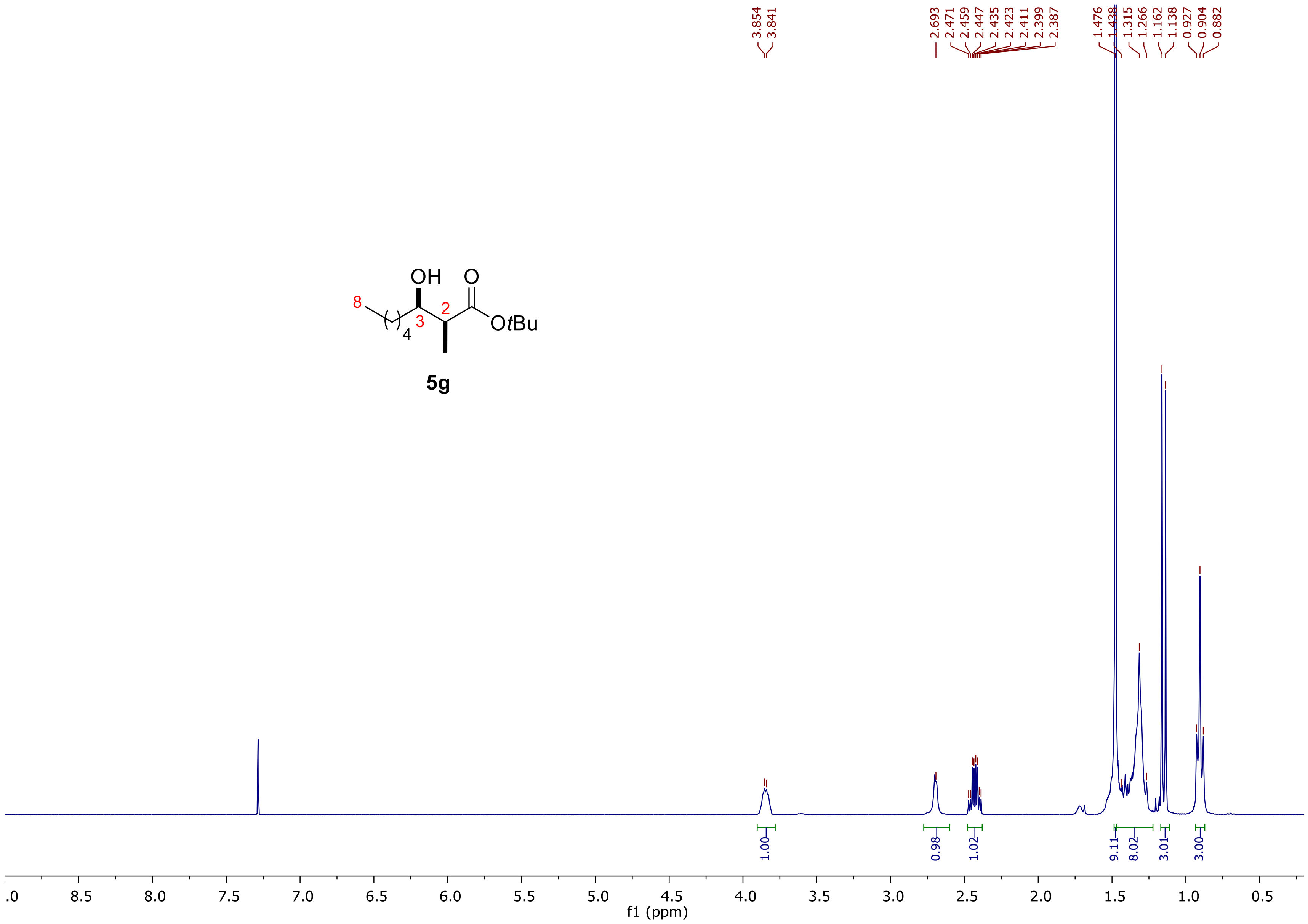

Figure S36

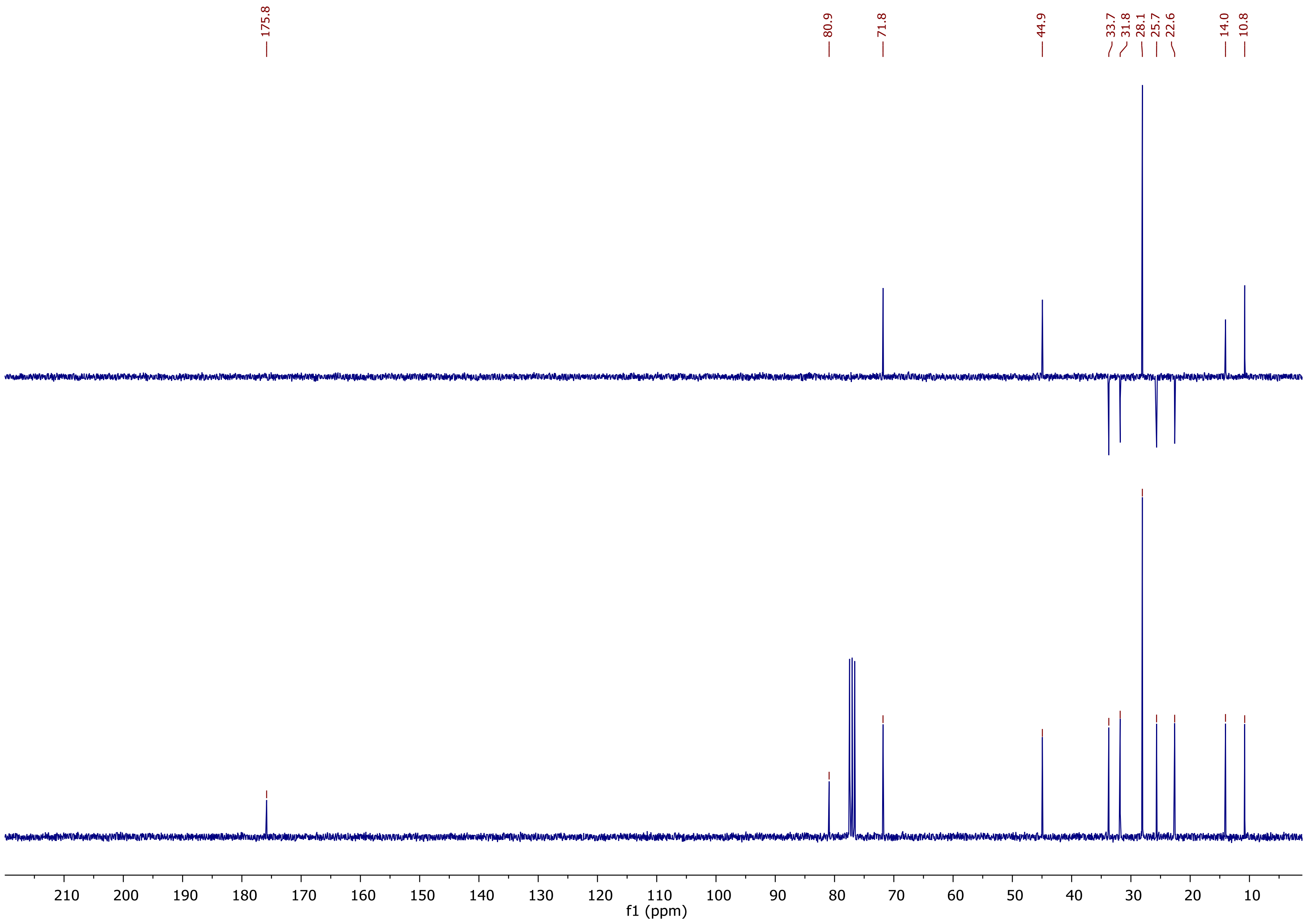

175.8
80.9
71.8
44.9
33.7
31.8
28.1
25.7
22.6
14.0
10.8
210
200
190
180
170
160
150
140
130
120
110
100
90
80
70
60
50
40
30
20
10
f1 (ppm)

Figure S37

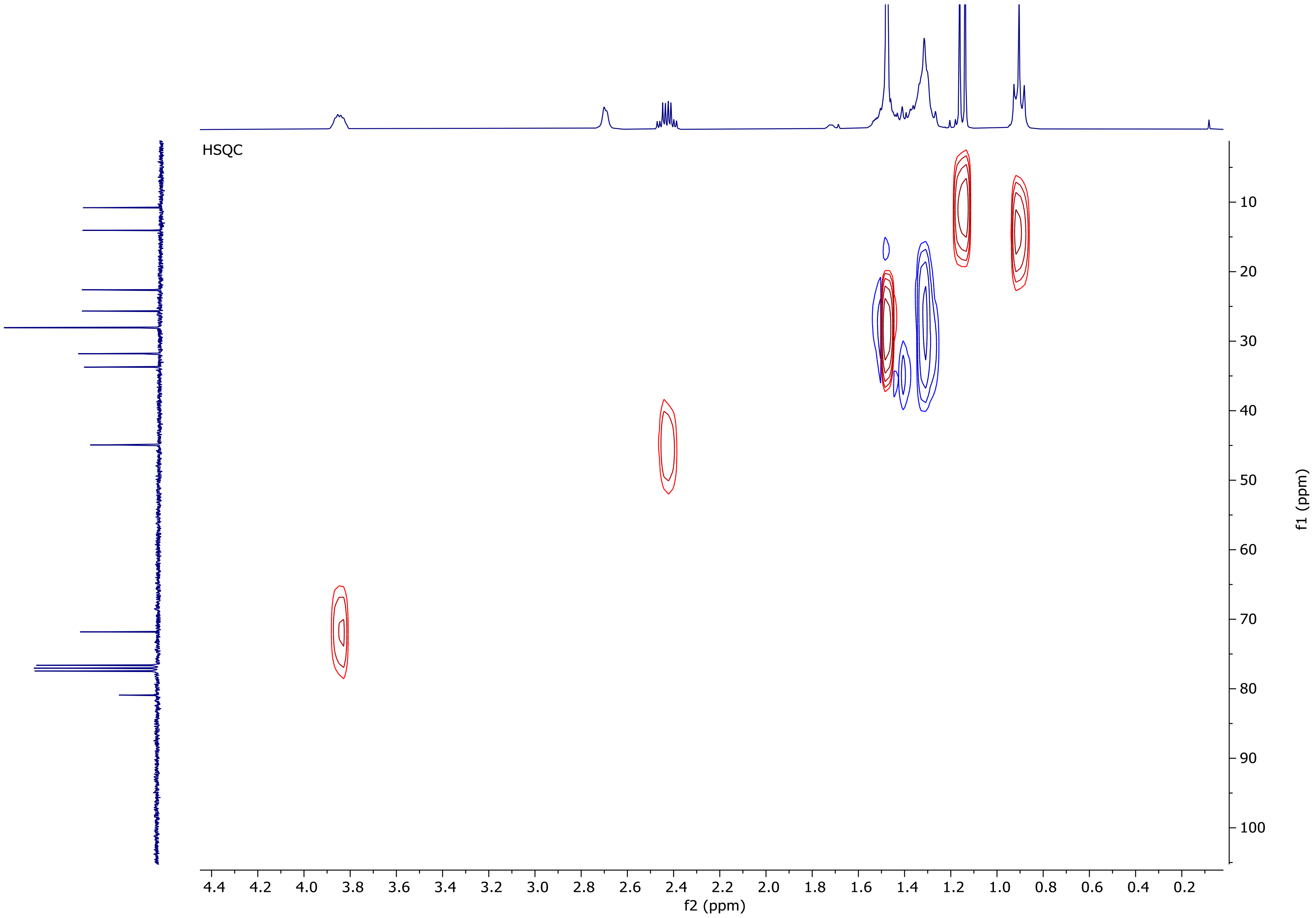
HSQC
4.4
4.2
4.0
3.8
3.6
3.4
3.2
3.0
2.8
2.6
2.4
2.2
2.0
1.8
1.6
1.4
1.2
1.0
0.8
0.6
0.4
0.2
f2 (ppm)
10
20
30
40
50
60
70
80
90
100
f1 (ppm)

Figure S38

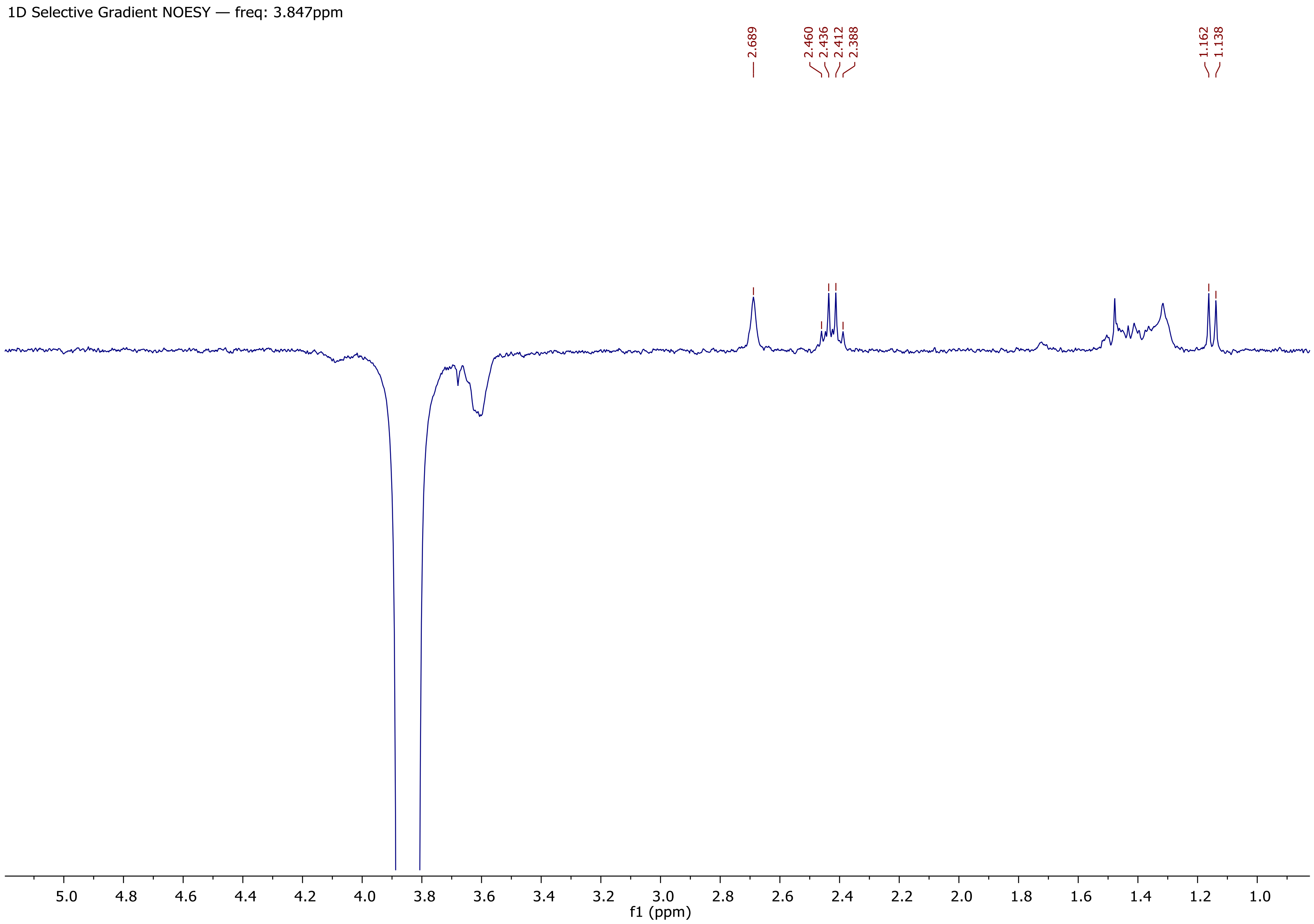

1D Selective Gradient NOESY — freq: 3.847ppm
2.689
2.460
2.436
2.412
2.388
1.162
1.138
5.0
4.8
4.6
4.4
4.2
4.0
3.8
3.6
3.4
3.2
3.0
2.8
2.6
2.4
2.2
2.0
1.8
1.6
1.4
1.2
1.0
f1 (ppm)

Figure S39

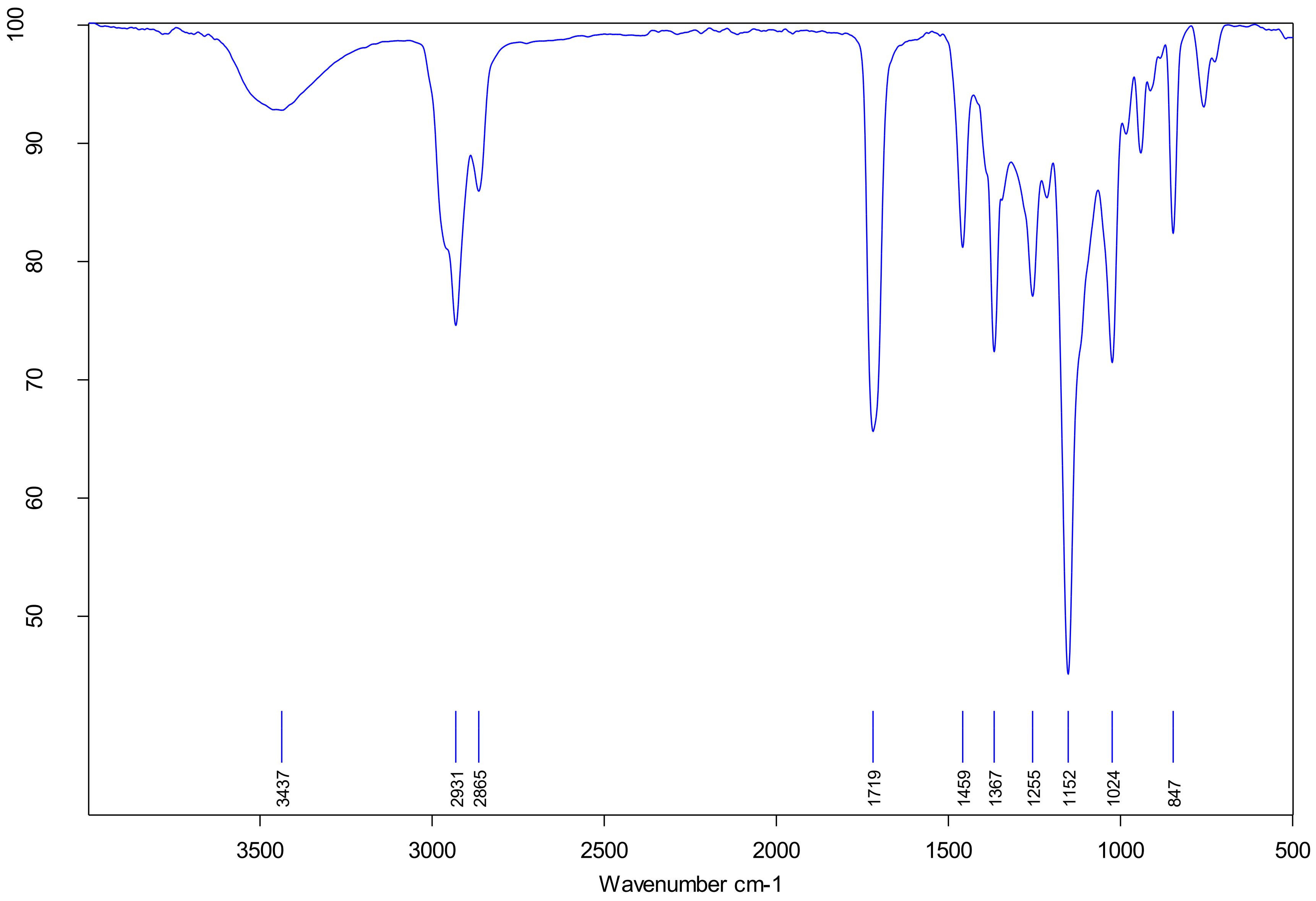

100
90
80
70
60
50
3437
2931
2865
1719
1459
1367
1255
1152
1024
847
3500
3000
2500
2000
1500
1000
500
Wavenumber cm-1

Figure S40

^{1}H NMR, DEPT 135, ^{13}C NMR and IR of 5h

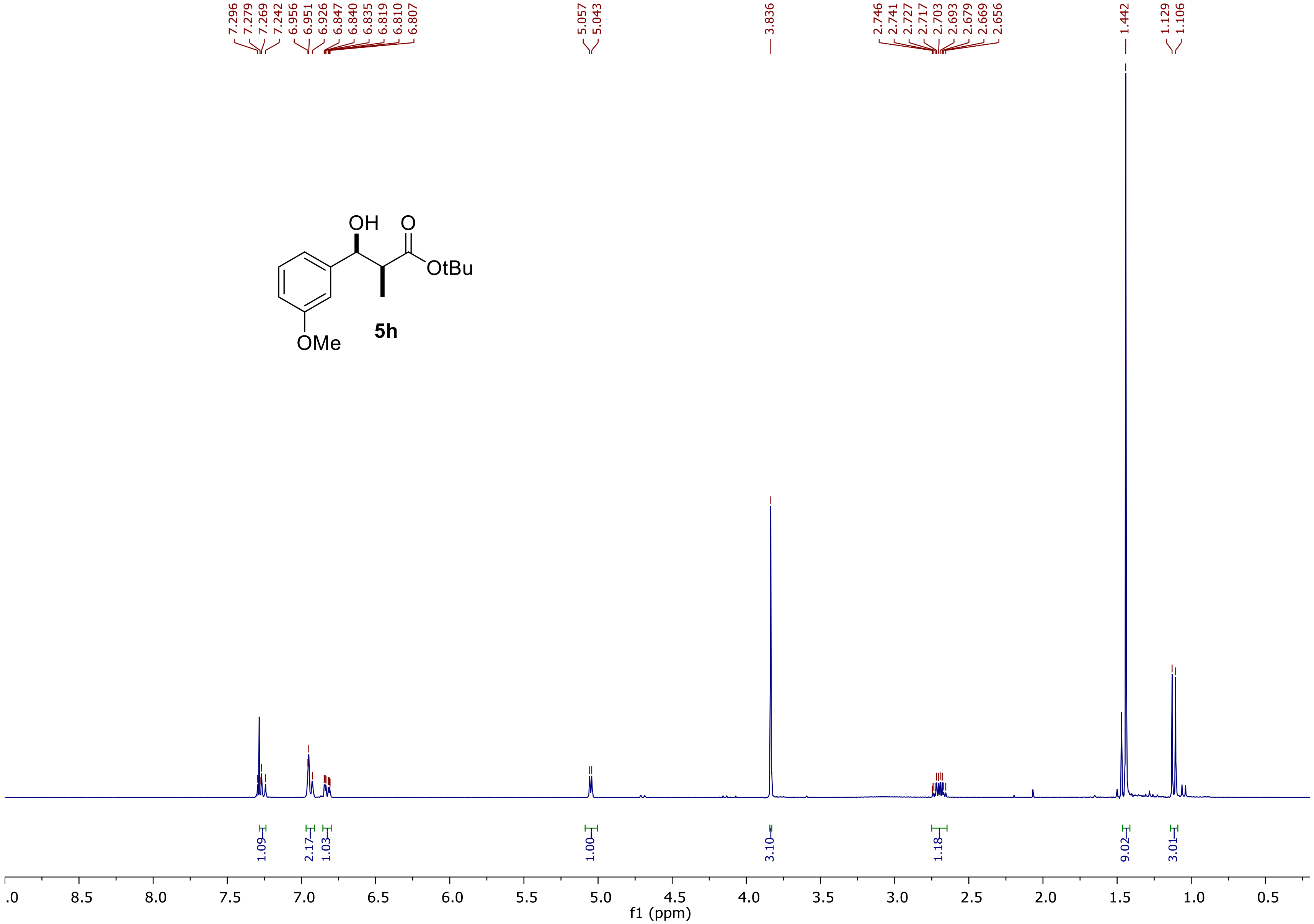

Figure S41

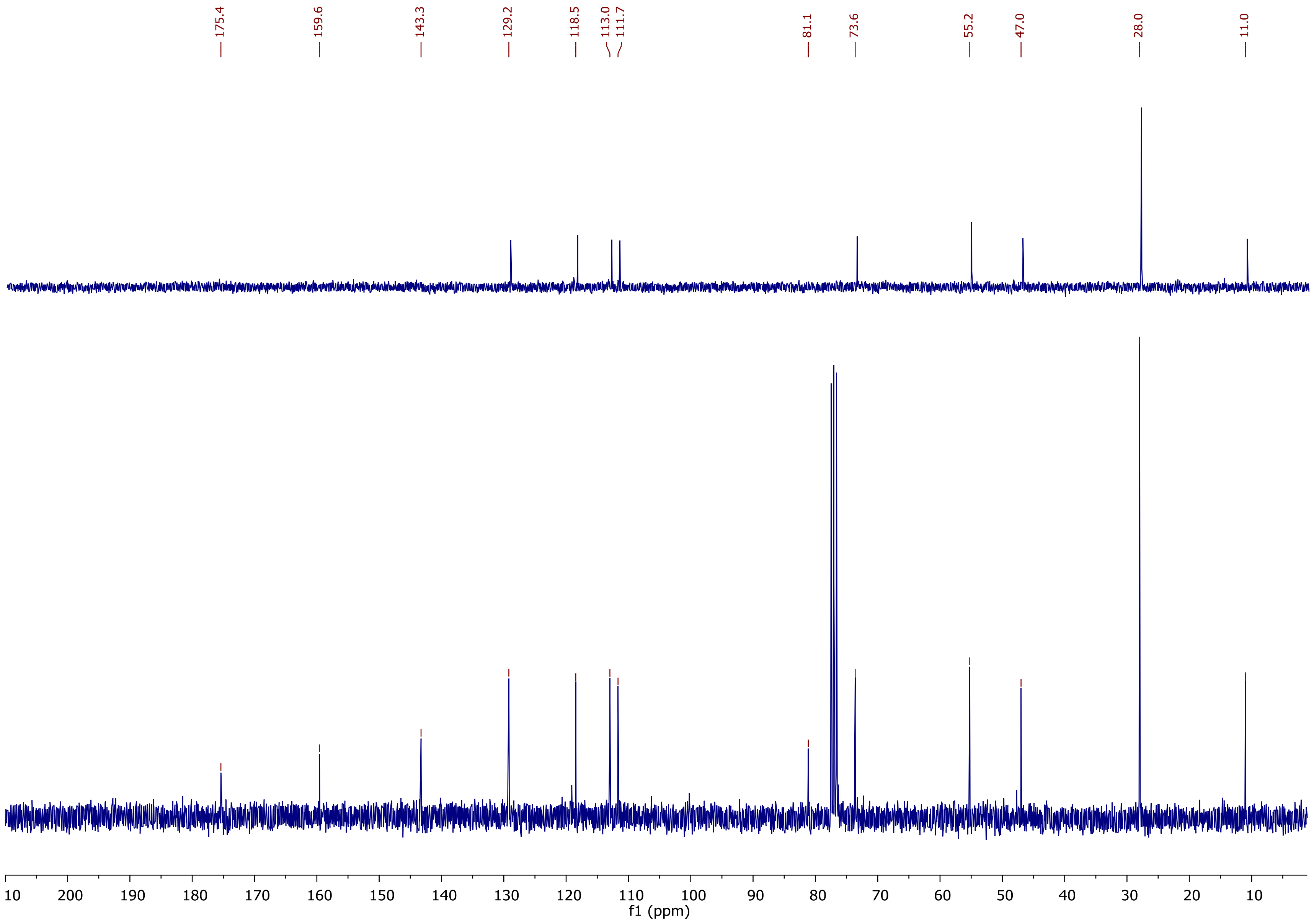
175.4
159.6
143.3
129.2
118.5
113.0
111.7
81.1
73.6
55.2
47.0
28.0
11.0
f1 (ppm)

Figure S42

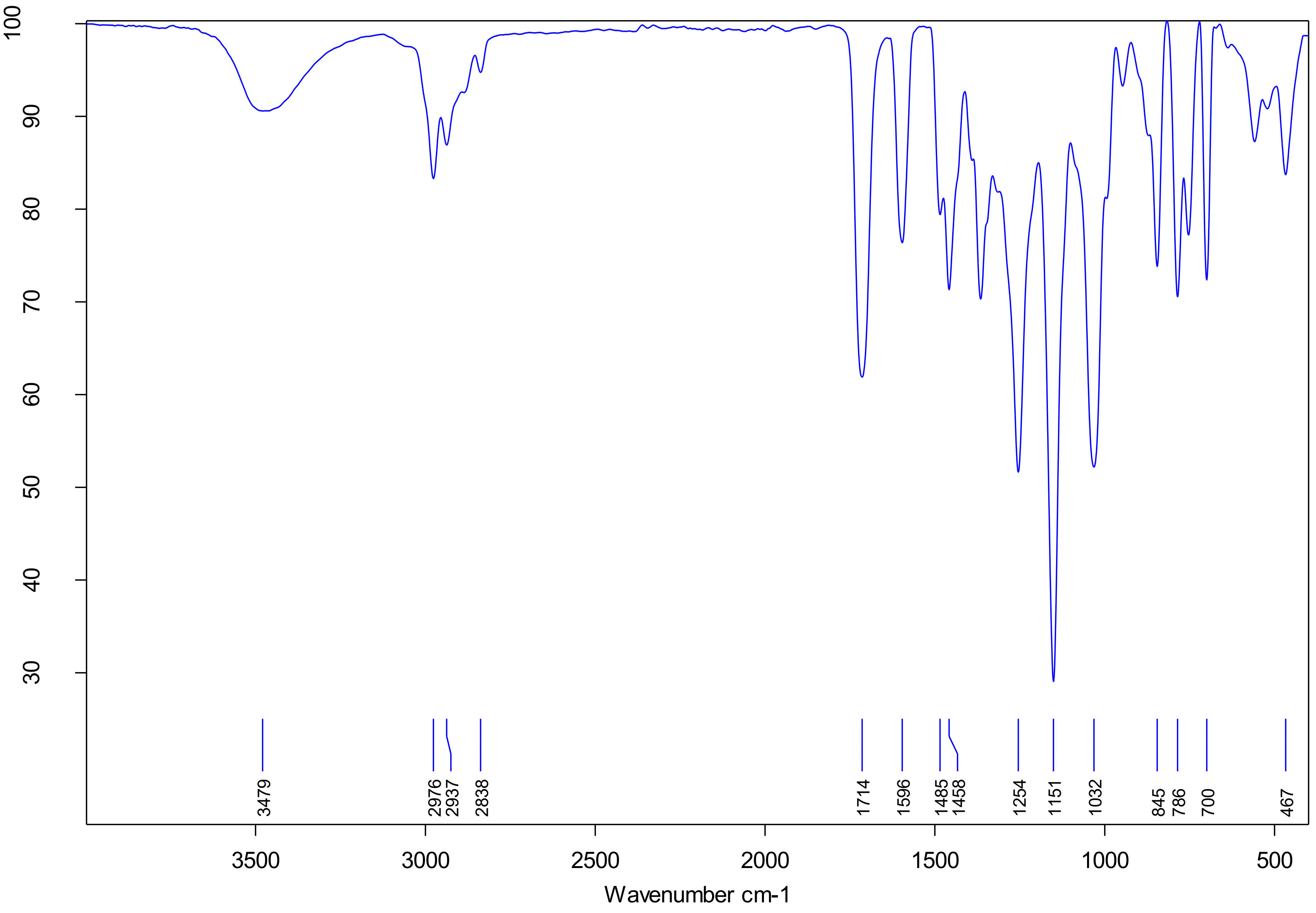

100
90
80
70
60
50
40
30
3479
2976
2937
2838
1714
1596
1485
1458
1254
1151
1032
845
786
700
467
3500
3000
2500
2000
1500
1000
500
Wavenumber cm-1

Figure S43

^{1}H NMR, DEPT 135, ^{13}C NMR and IR of 9

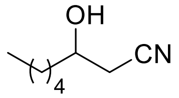

9

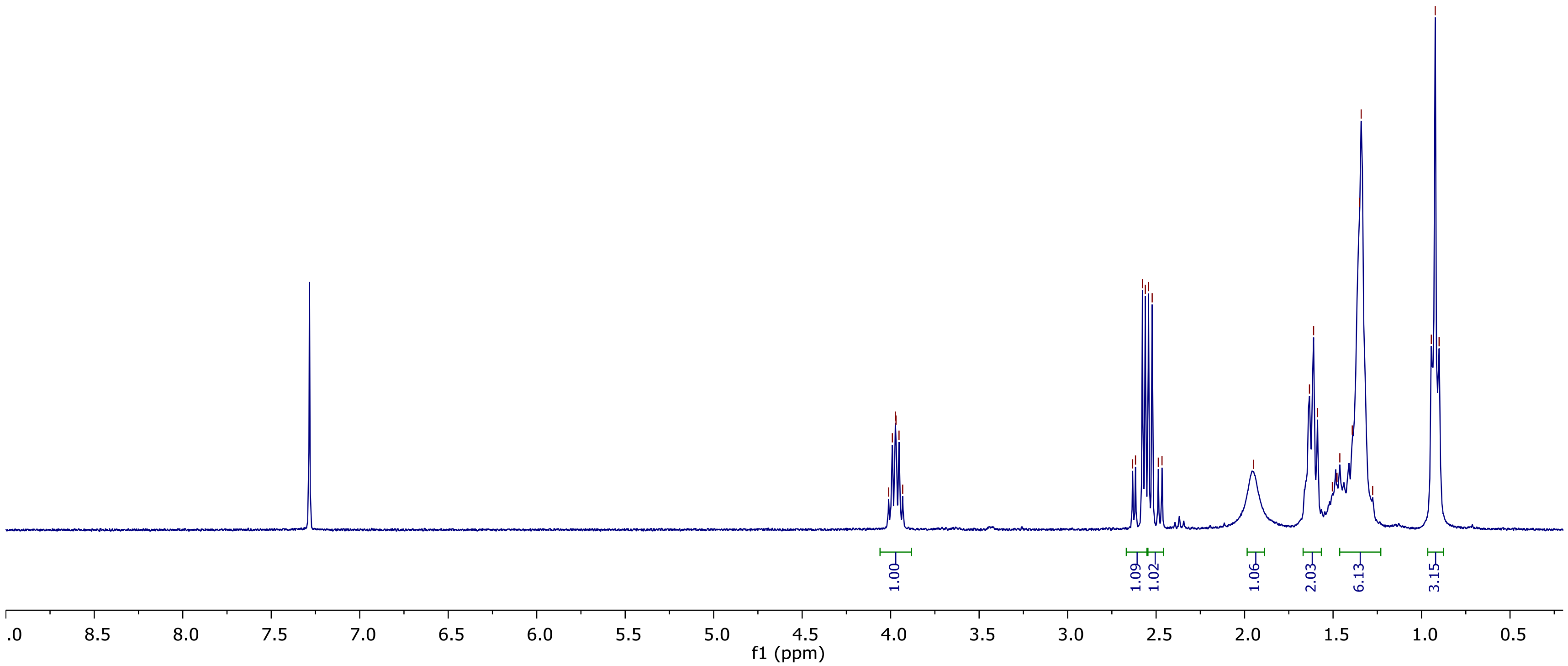

Figure S44

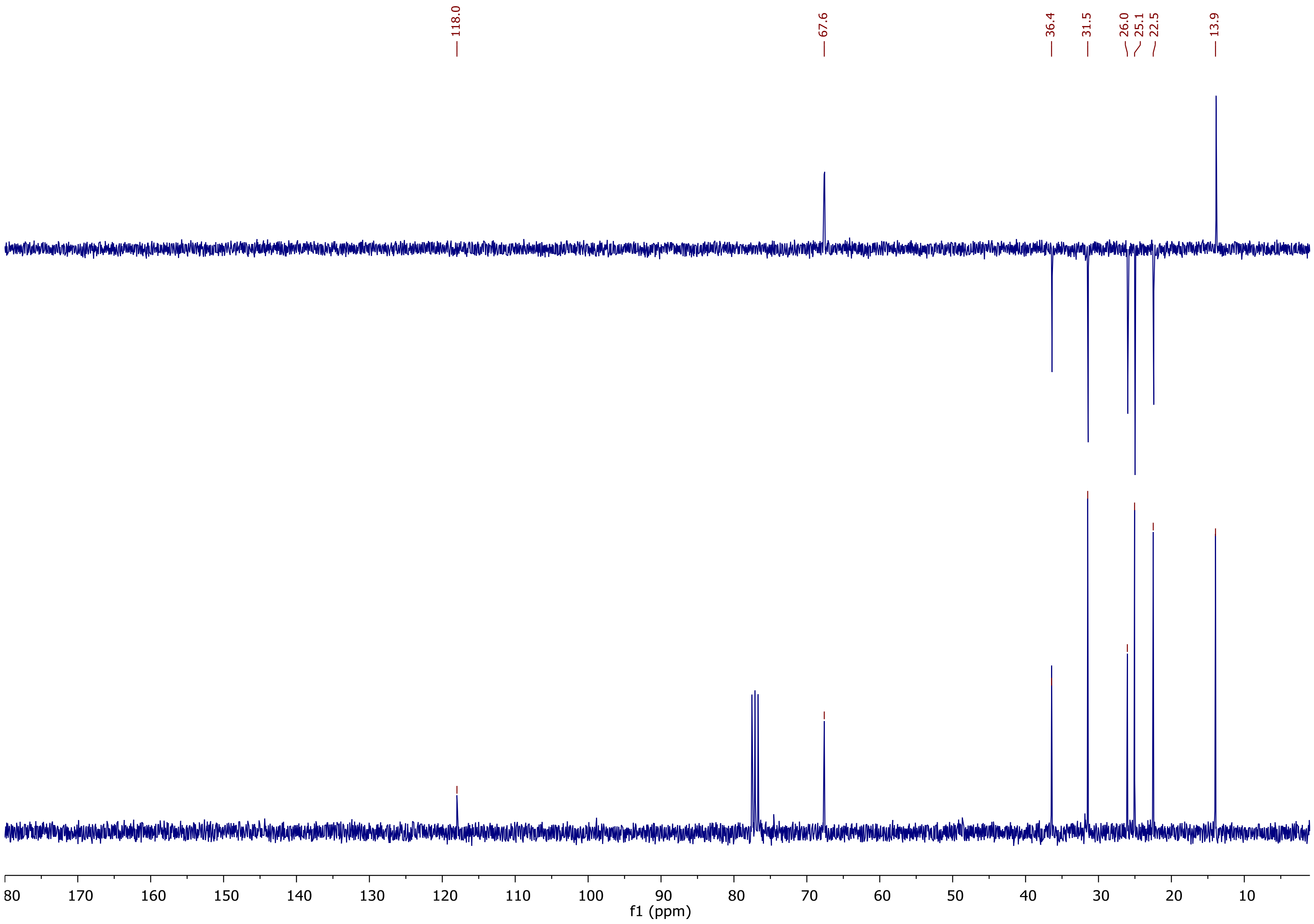

118.0
67.6
36.4
31.5
26.0
25.1
22.5
13.9
80
170
160
150
140
130
120
110
100
90
80
70
60
50
40
30
20
10
f1 (ppm)

Figure S45

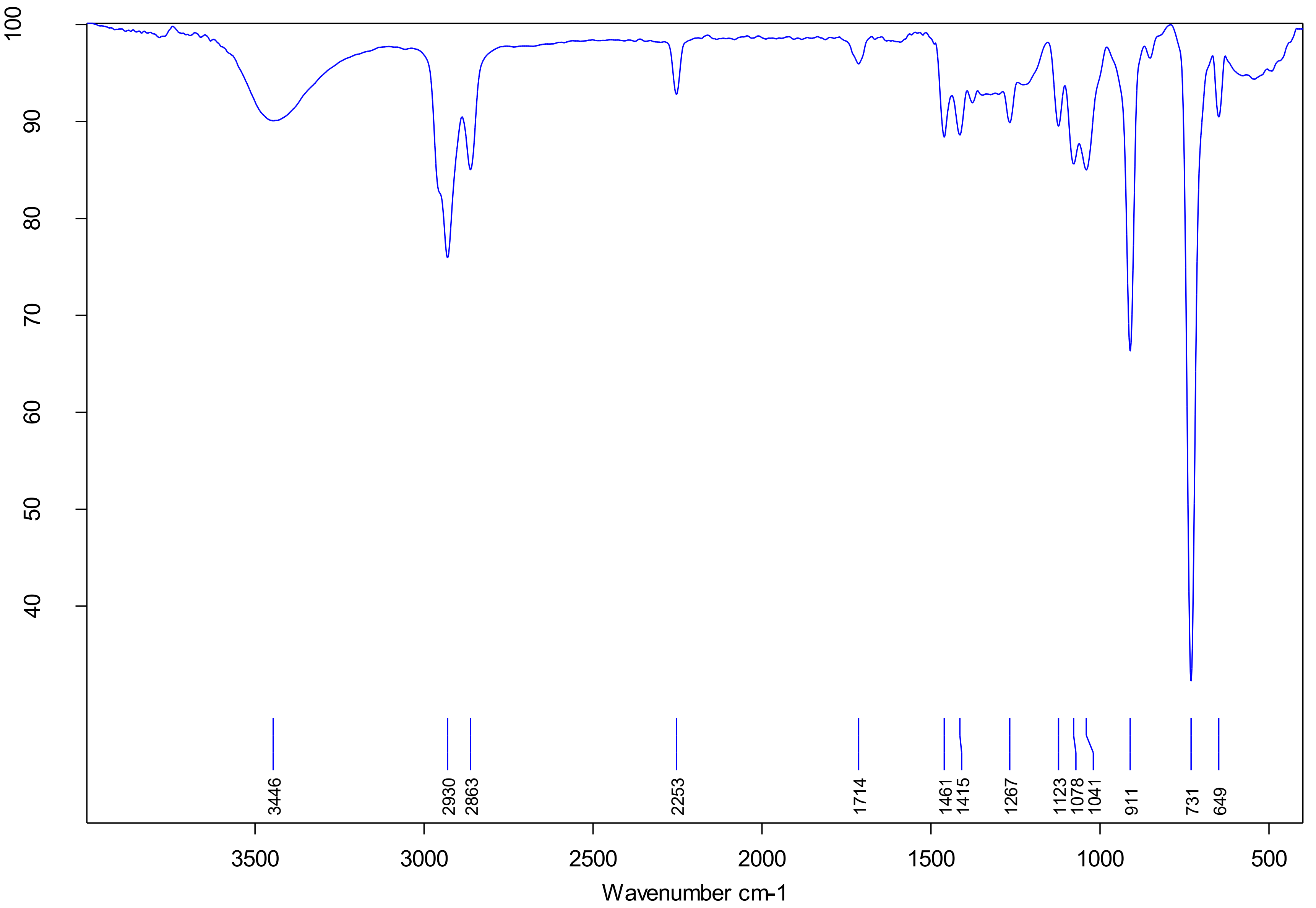
100
90
80
70
60
50
40
3446
2930
2863
2253
1714
1461
1415
1267
1123
1078
1041
911
731
649
3500
3000
2500
2000
1500
1000
500
Wavenumber cm-1

Figure S46

^{1}H NMR, DEPT 135, ^{13}C NMR and IR of 10

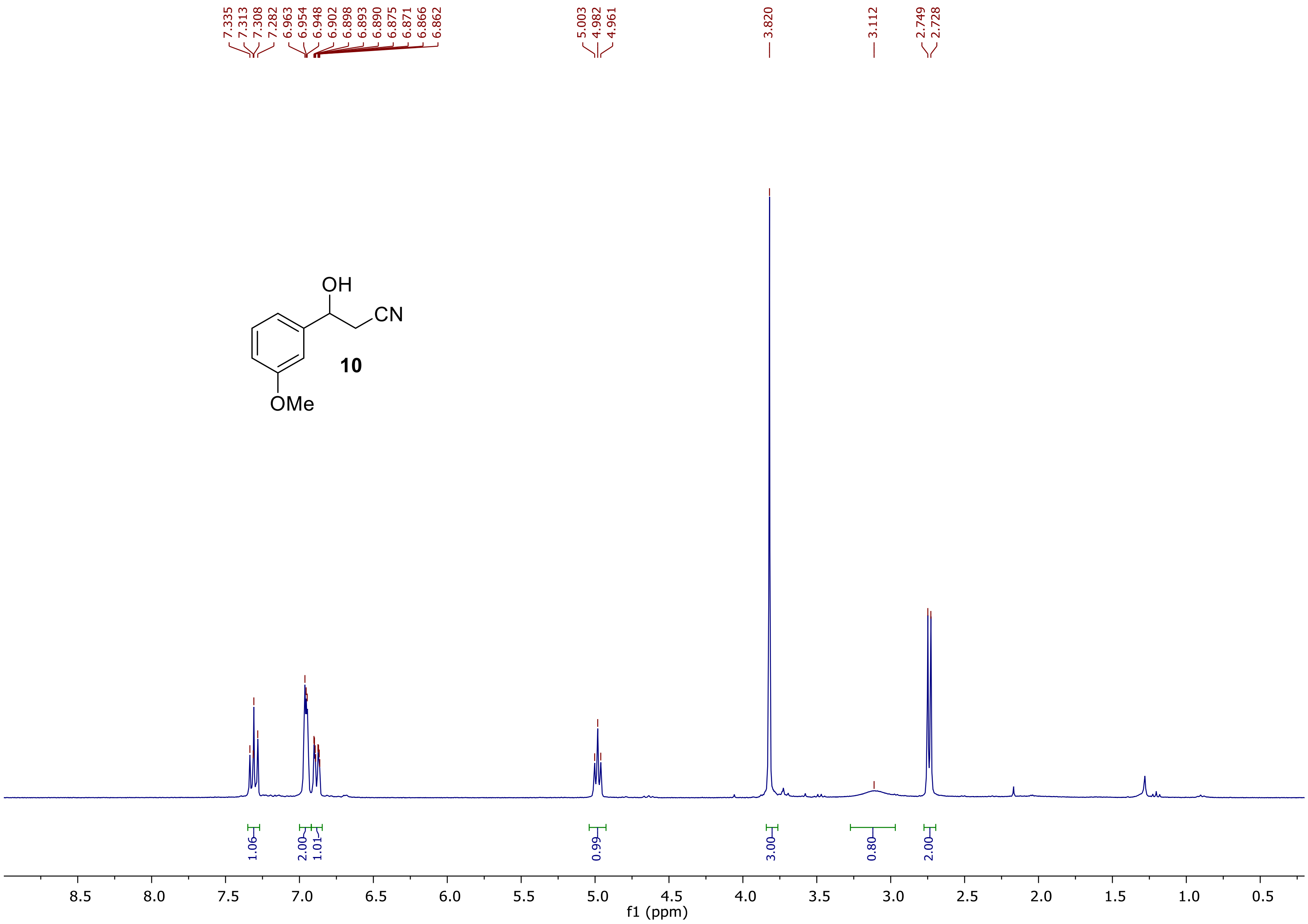

Figure S47

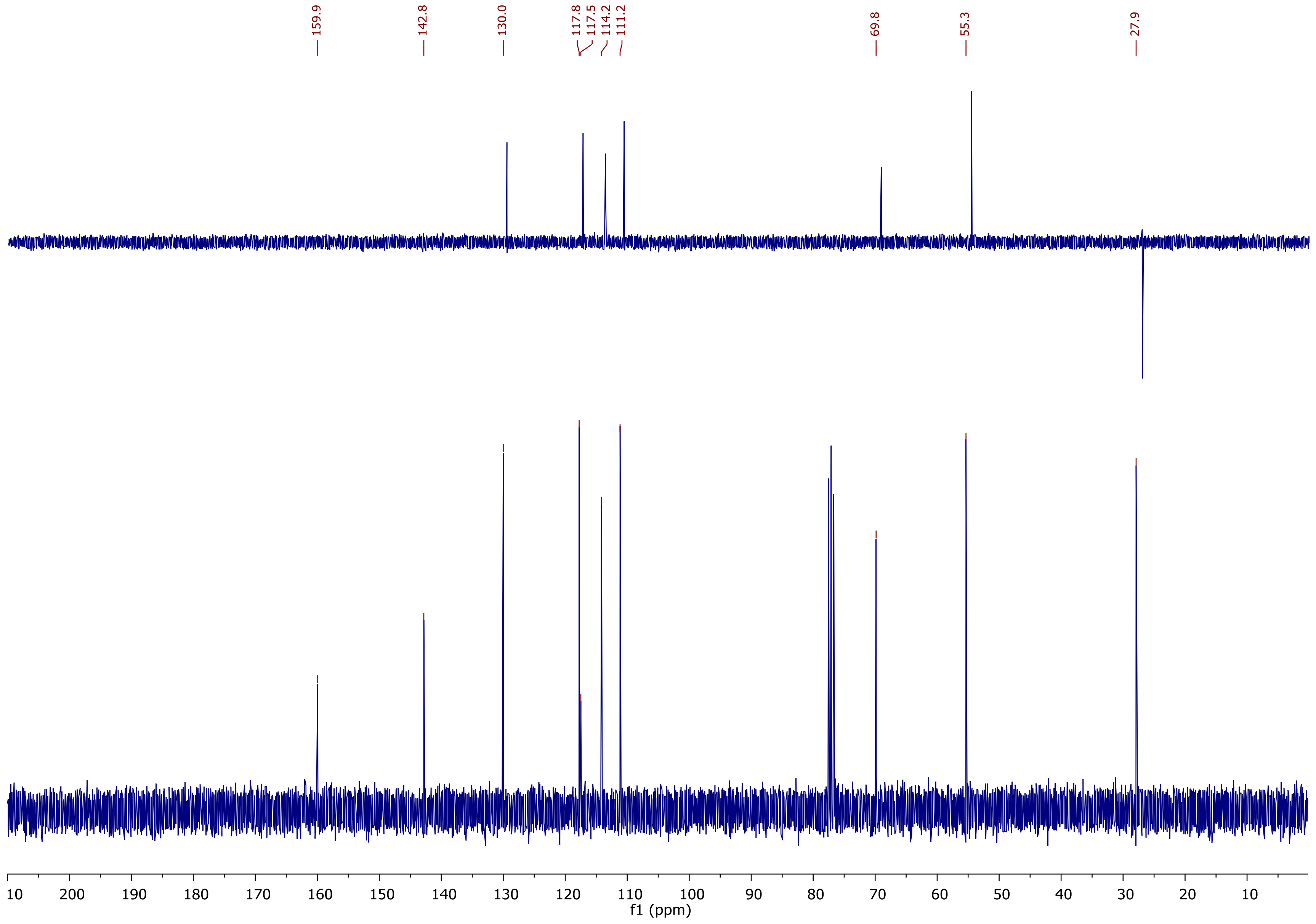

Figure S48

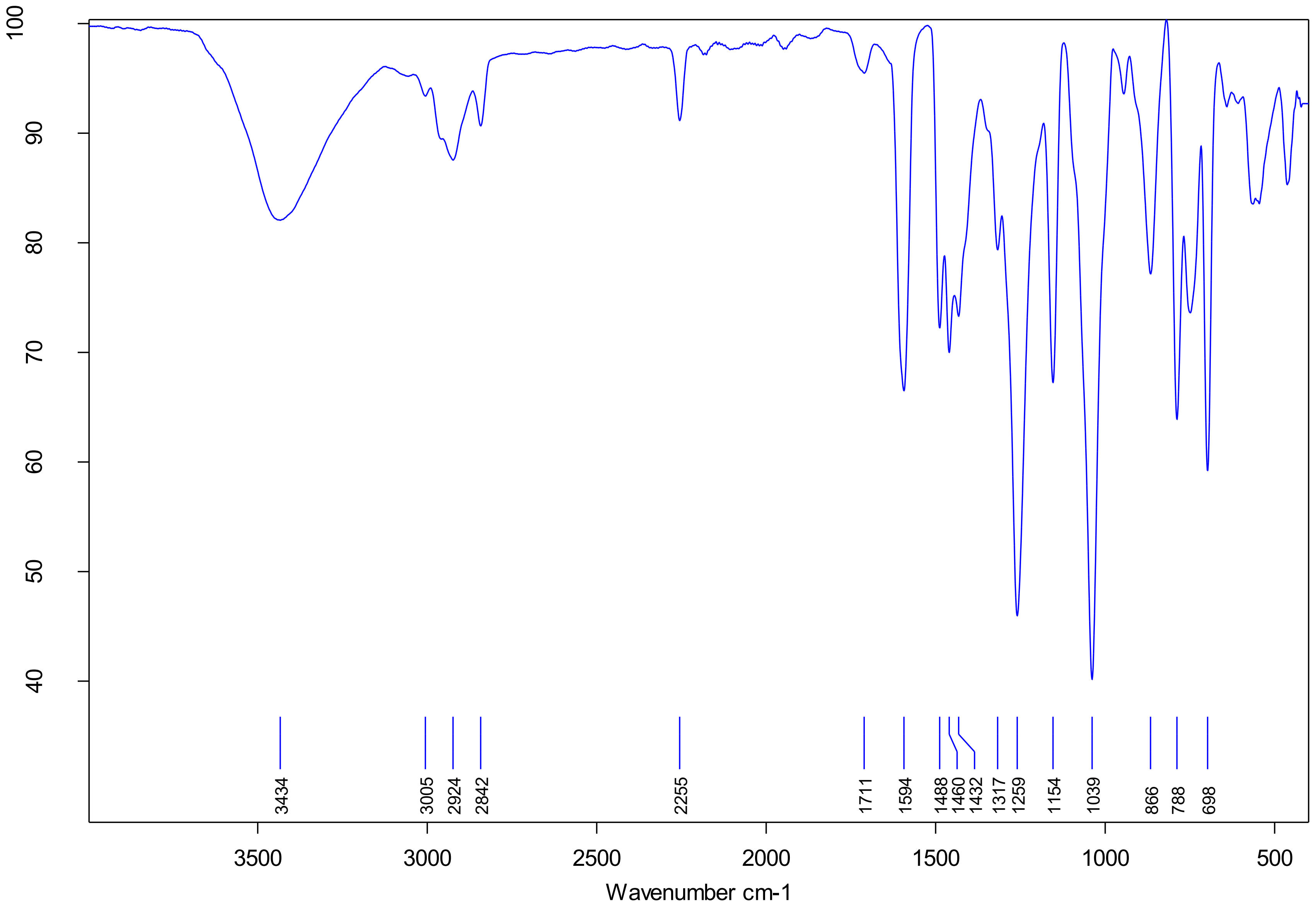

100
90
80
70
60
50
40
3434
3005
2924
2842
2255
1711
1594
1488
1460
1432
1317
1259
1154
1039
866
788
698
3500
3000
2500
2000
1500
1000
500
Wavenumber cm-1

Figure S49

^{1}H NMR and DEPT 135, ^{13}C NMR of 1,2-bis(3-methoxyphenyl)ethane-1,2-diol

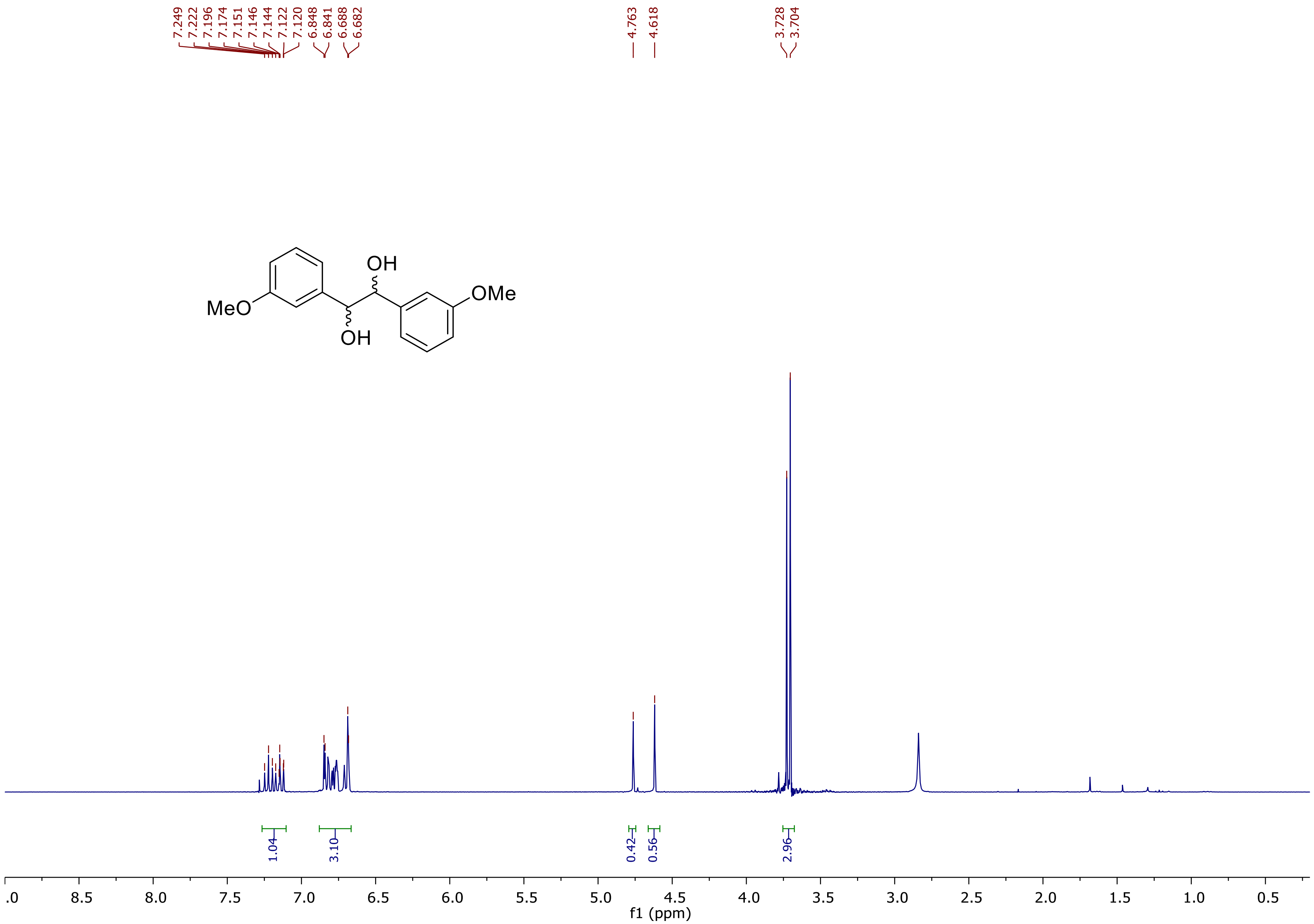

Figure S50

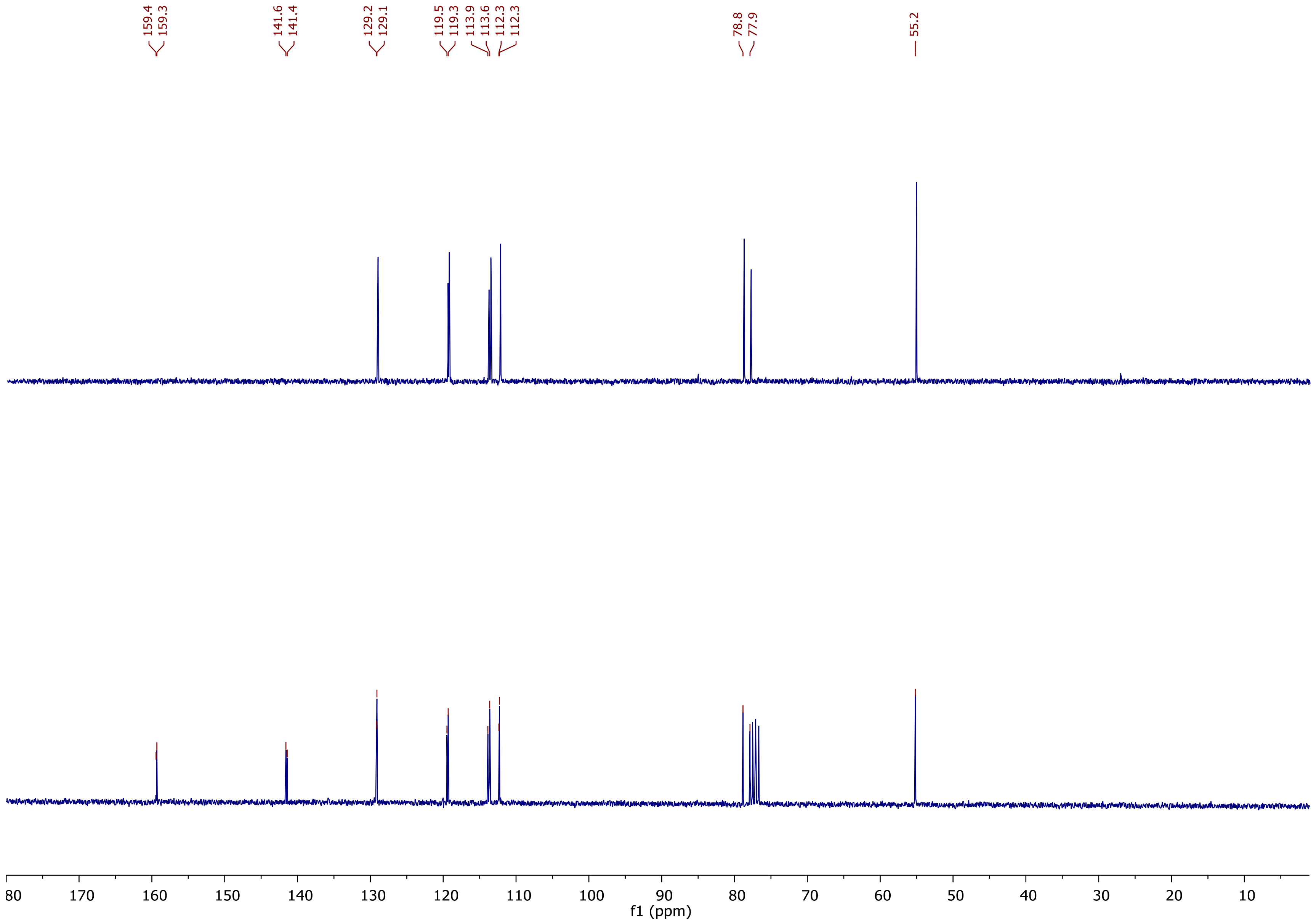
159.4
159.3
141.6
141.4
129.2
129.1
119.5
119.3
113.9
113.6
112.3
112.3
78.8
77.9
55.2
80
170
160
150
140
130
120
110
100
90
80
70
60
50
40
30
20
10
f1 (ppm)

Figure S51

^{1}H NMR, DEPT 135, ^{13}C NMR and IR of (2*R,3*R**)-2-methyltridec-12-ene-1,3-diol**

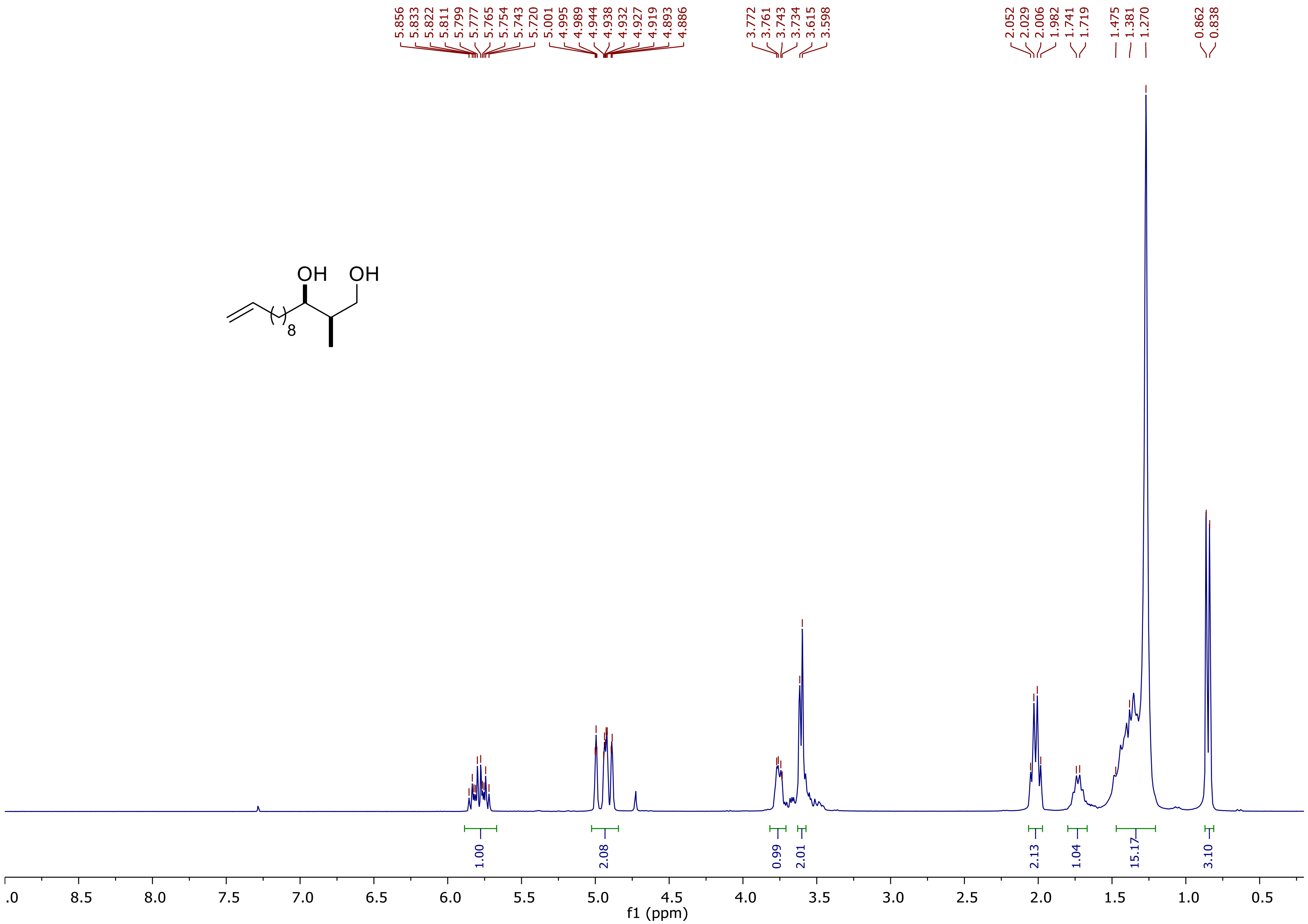

Figure S52

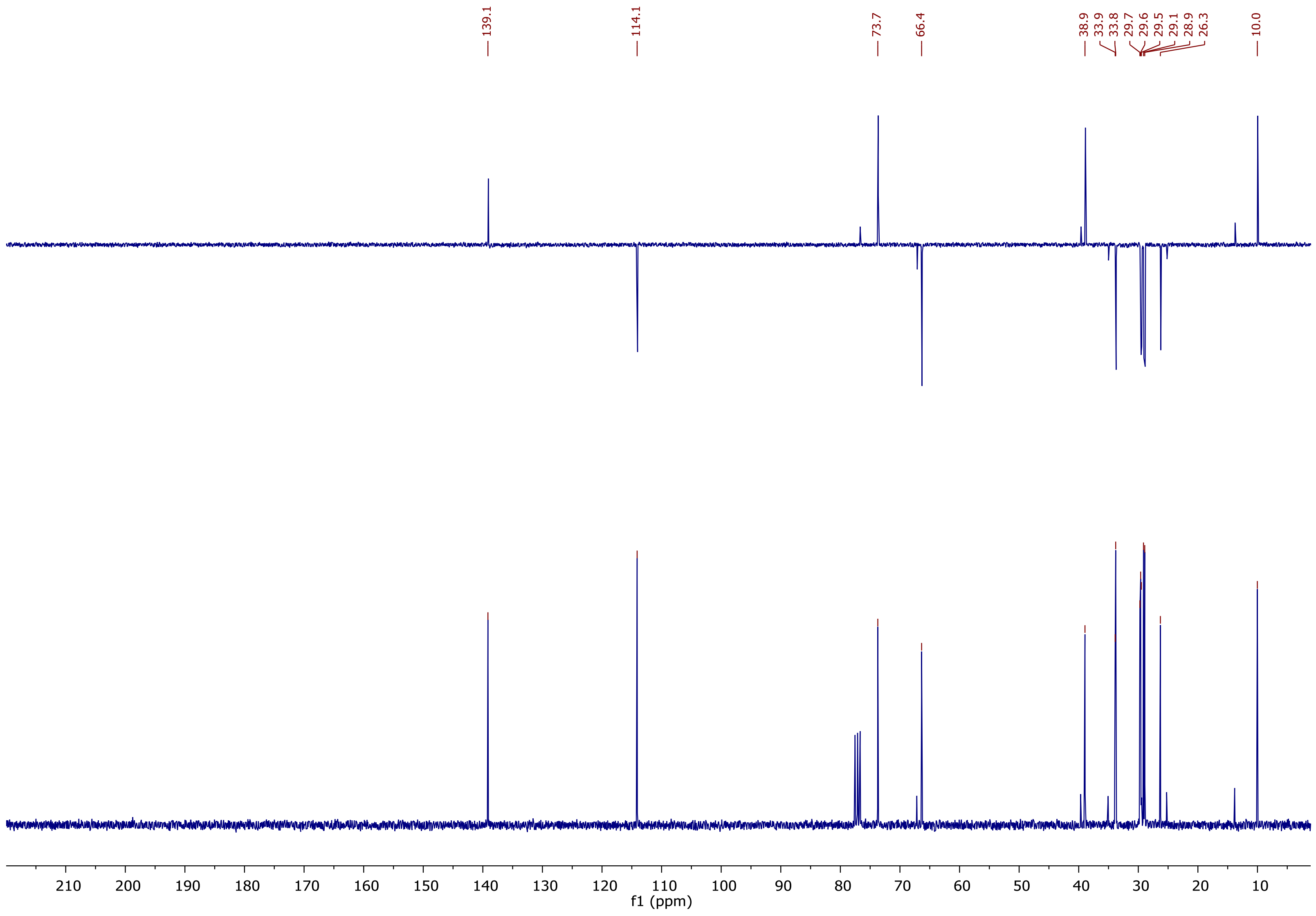
139.1
114.1
73.7
66.4
38.9
33.9
33.8
29.7
29.6
29.5
29.1
28.9
26.3
10.0
210 200 190 180 170 160 150 140 130 120 110 100 90 80 70 60 50 40 30 20 10
f1 (ppm)

Figure S53

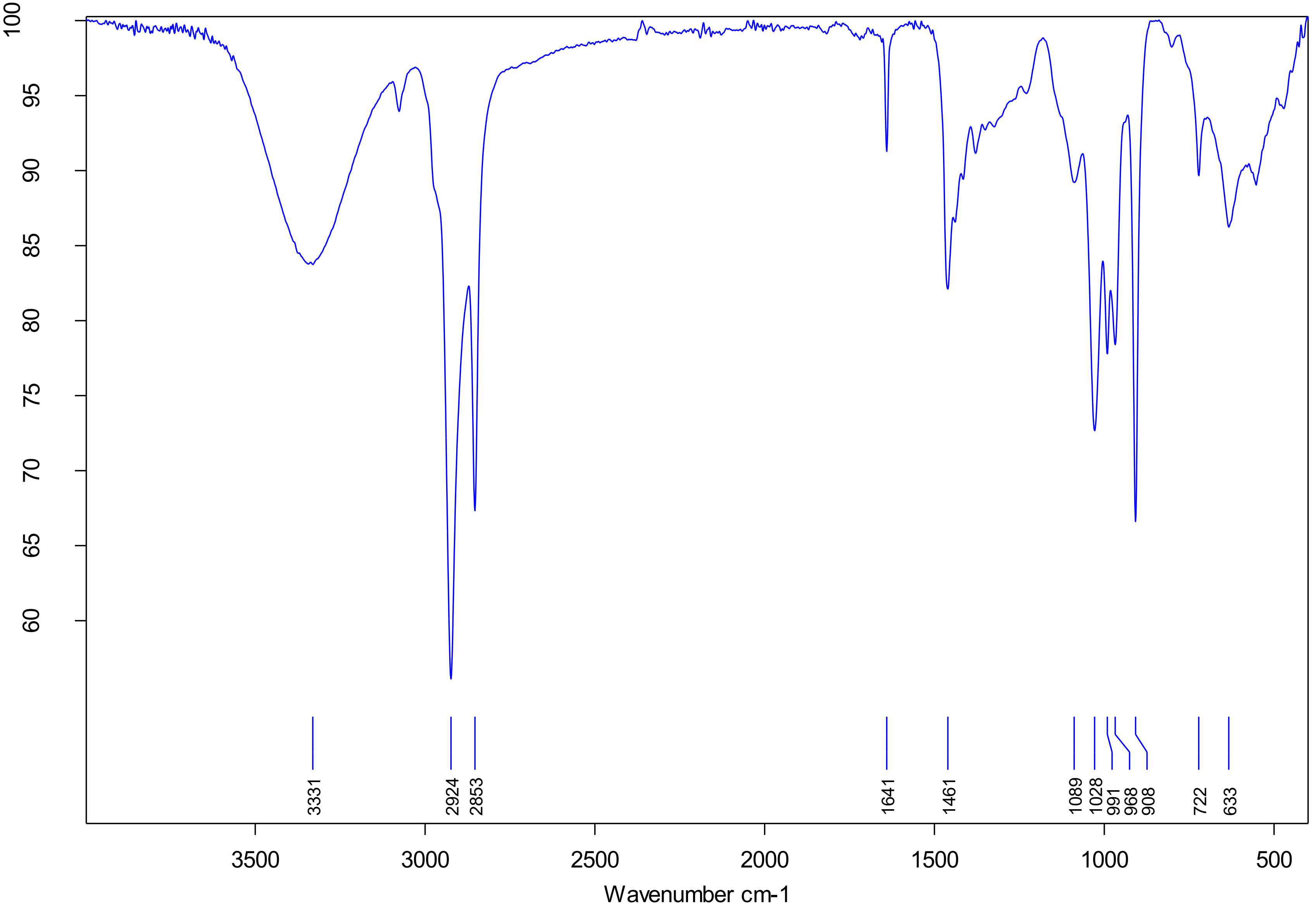
100
95
90
85
80
75
70
65
60
3331
2924
2853
1641
1461
1089
1028
991
968
908
722
633
3500
3000
2500
2000
1500
1000
500
Wavenumber cm-1

Figure S54

^{1}H NMR, DEPT 135, ^{13}C NMR, HSQC, NOESY 2D and NOESY 1D of 6

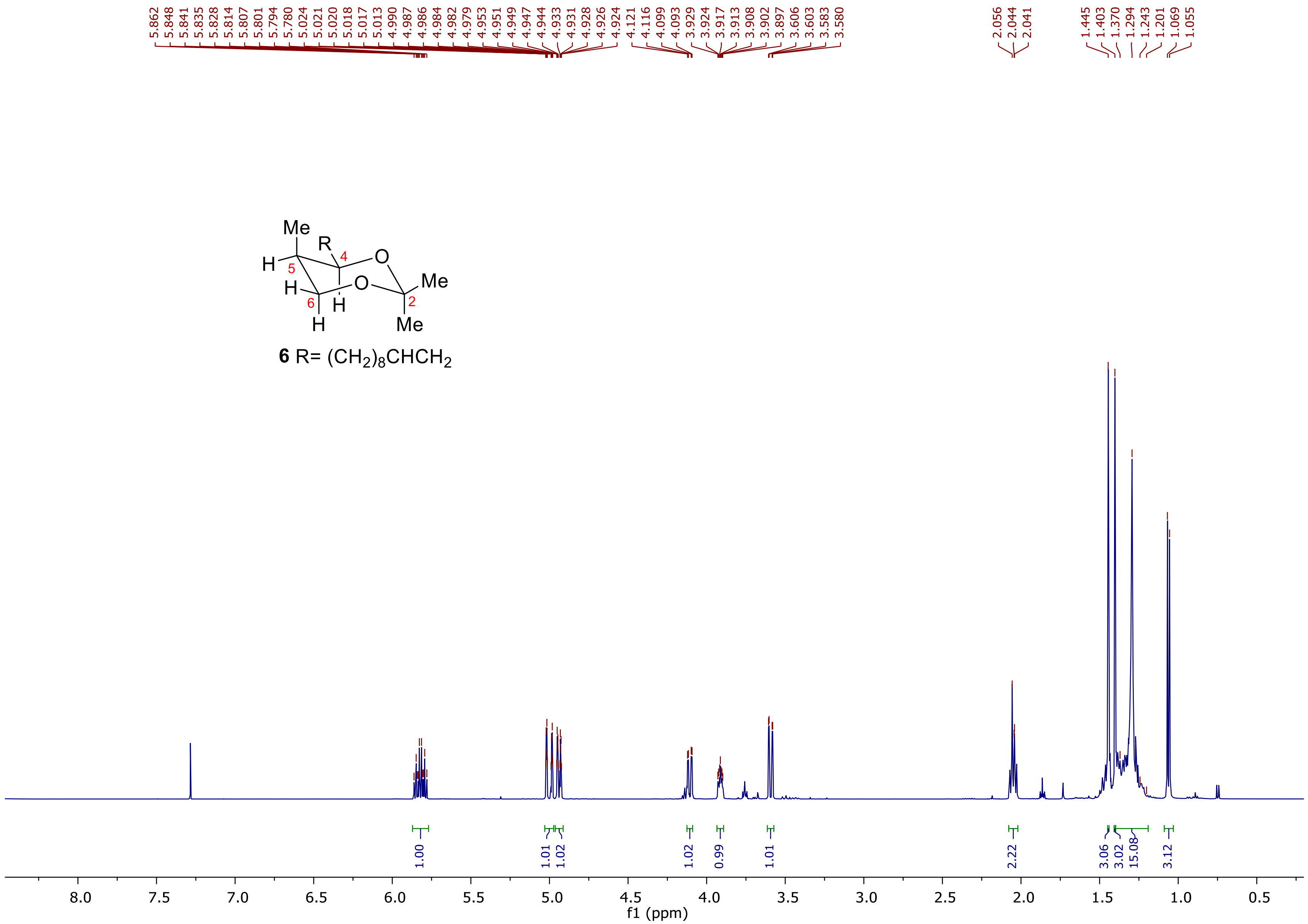

Figure S55

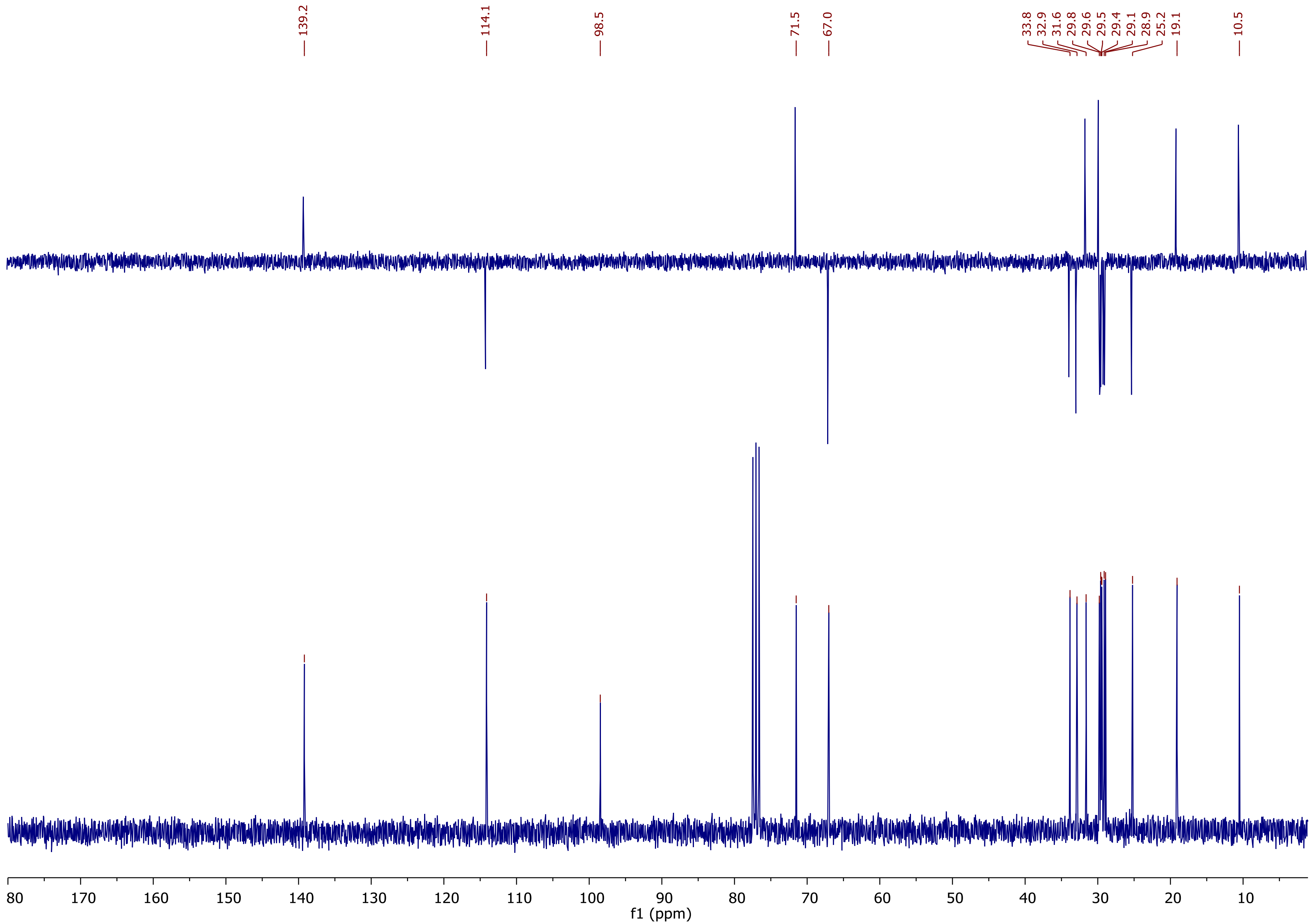
139.2
114.1
98.5
71.5
67.0
33.8
32.9
31.6
29.8
29.6
29.5
29.4
29.1
28.9
25.2
19.1
10.5
80 170 160 150 140 130 120 110 100 90 80 70 60 50 40 30 20 10
f1 (ppm)

Figure S56

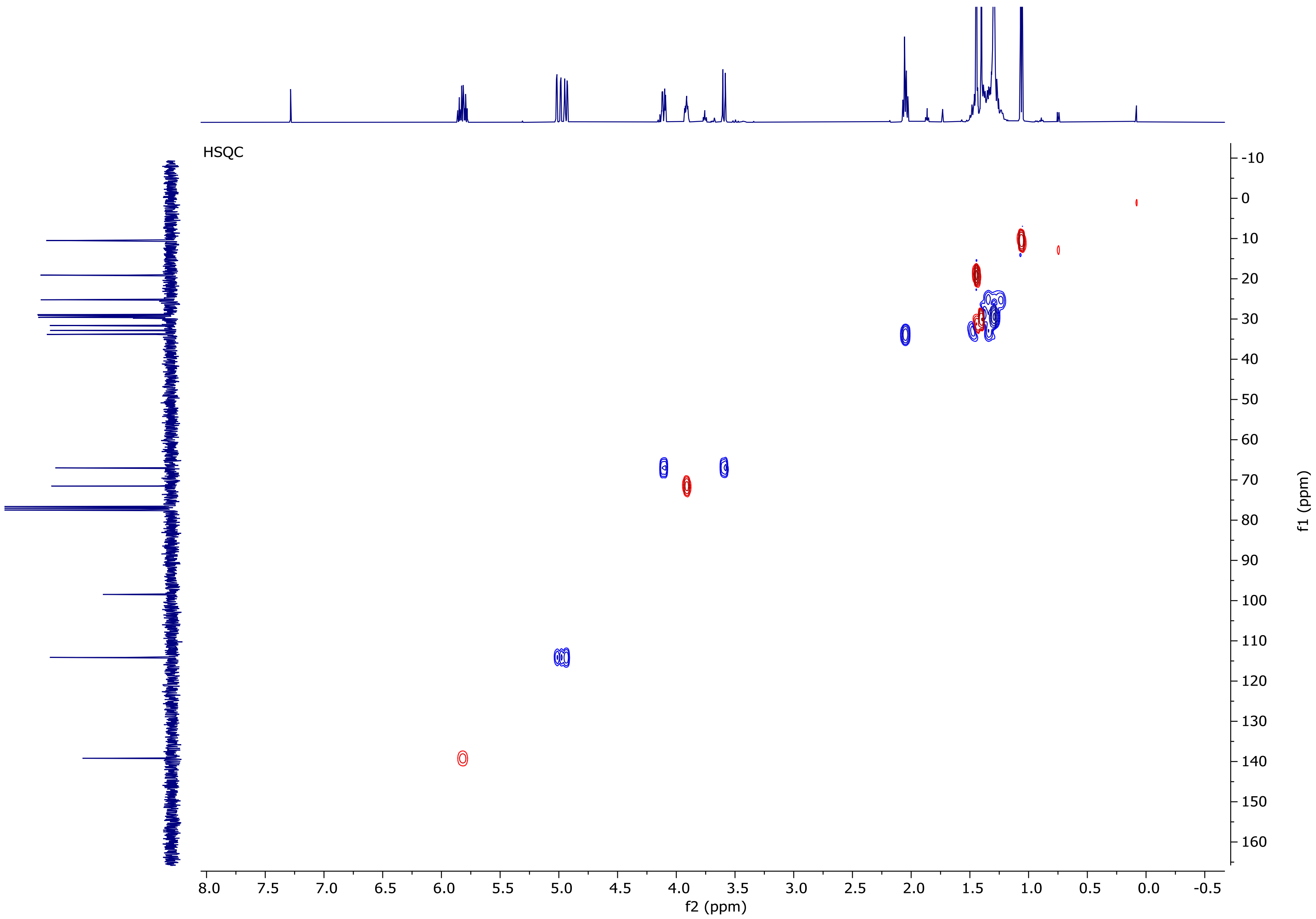

HSQC
8.0
7.5
7.0
6.5
6.0
5.5
5.0
4.5
4.0
3.5
3.0
2.5
2.0
1.5
1.0
0.5
0.0
-0.5
f2 (ppm)
-10
0
10
20
30
40
50
60
70
80
90
100
110
120
130
140
150
160
f1 (ppm)

Figure S57

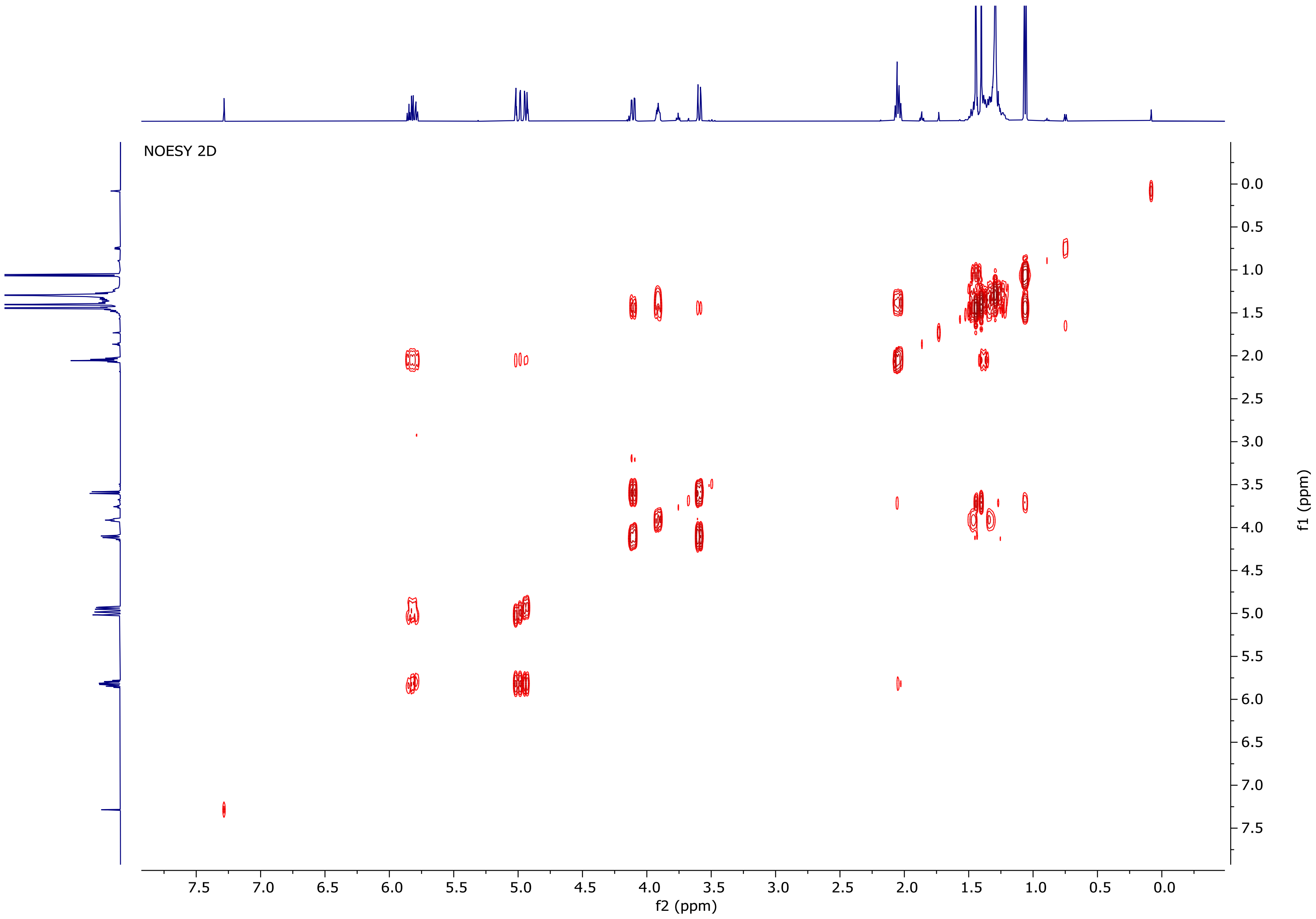

Figure S58

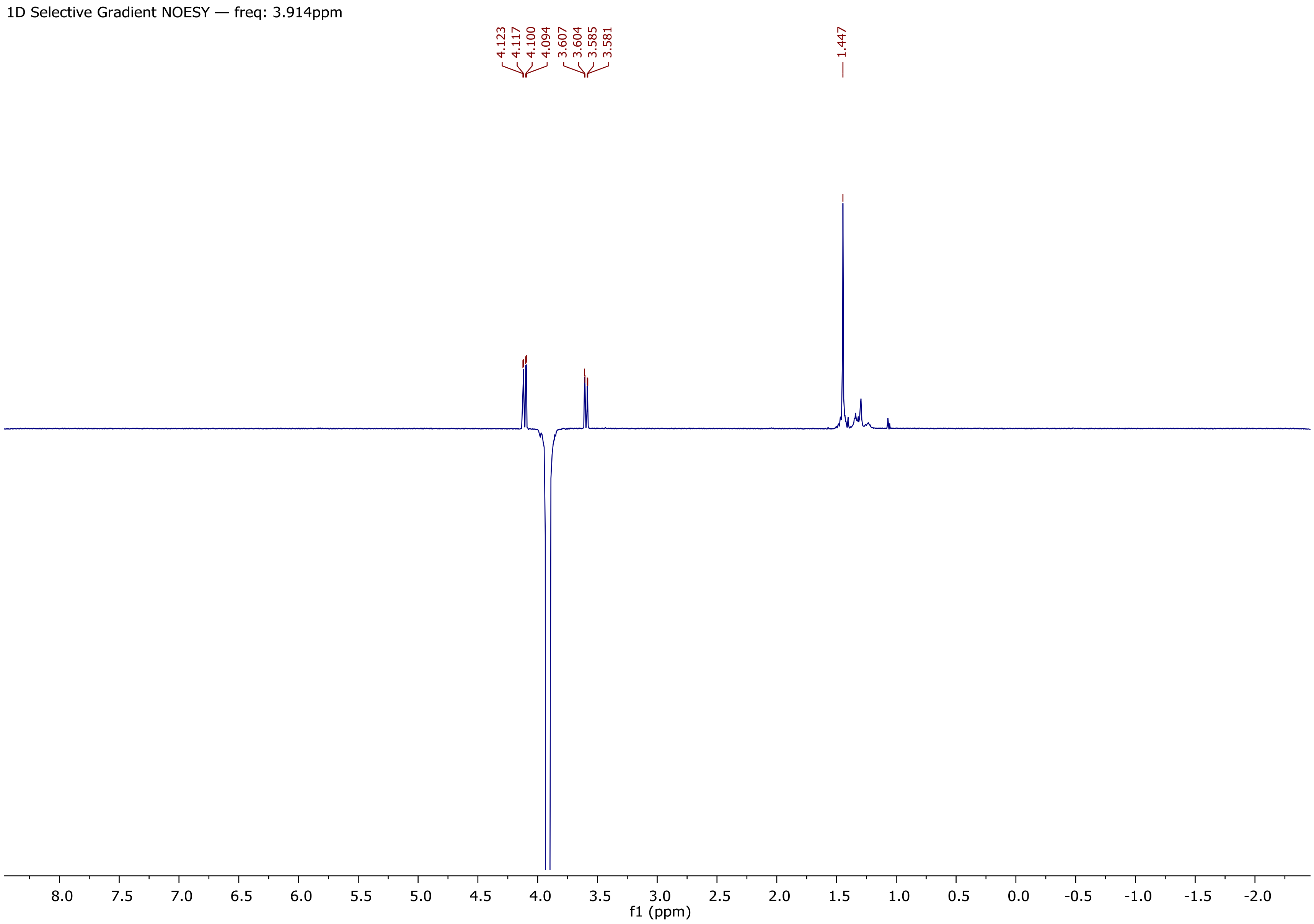
1D Selective Gradient NOESY — freq: 3.914ppm
4.123
4.117
4.100
4.094
3.607
3.604
3.585
3.581
1.447
8.0
7.5
7.0
6.5
6.0
5.5
5.0
4.5
4.0
3.5
3.0
2.5
2.0
1.5
1.0
0.5
0.0
-0.5
-1.0
-1.5
-2.0
f1 (ppm)

Figure S59

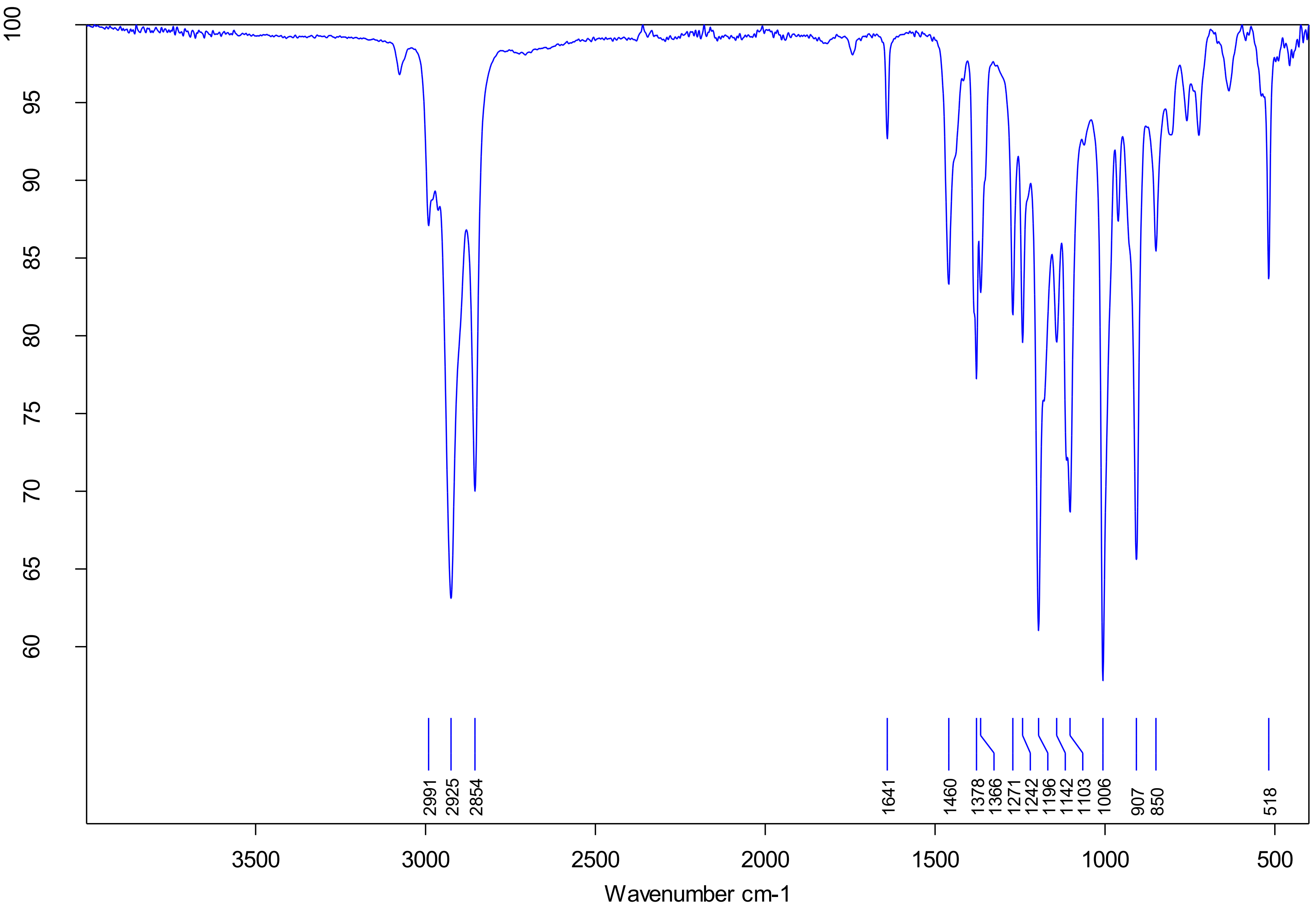

100
95
90
85
80
75
70
65
60
2991
2925
2854
1641
1460
1378
1366
1271
1242
1196
1142
1103
1006
907
850
518
3500
3000
2500
2000
1500
1000
500
Wavenumber cm-1

Figure S60

^{1}H NMR, DEPT 135, ^{13}C NMR and IR of (2*R**,3*R**)-2-methyloctane-1,3-diol

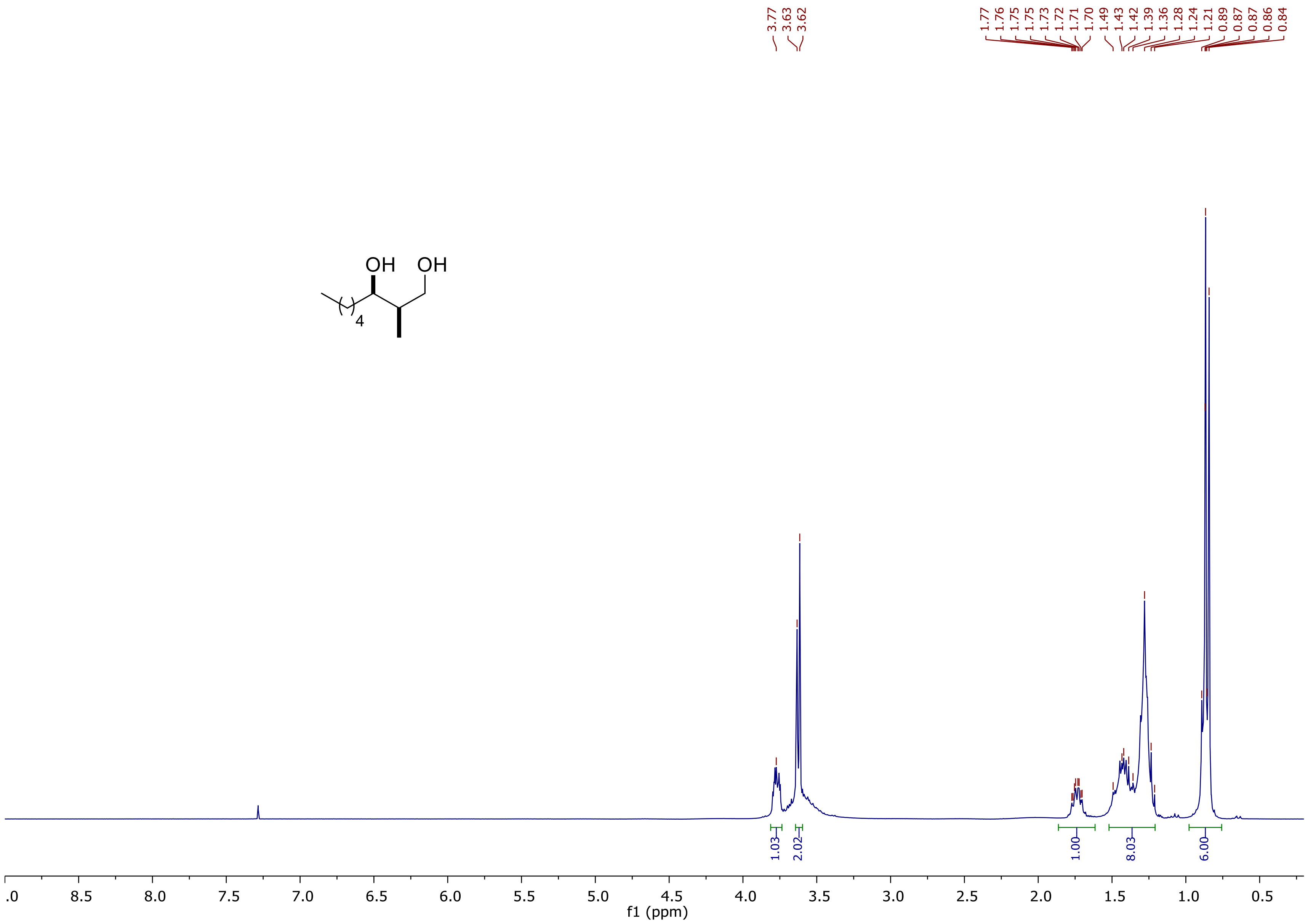

Figure S61

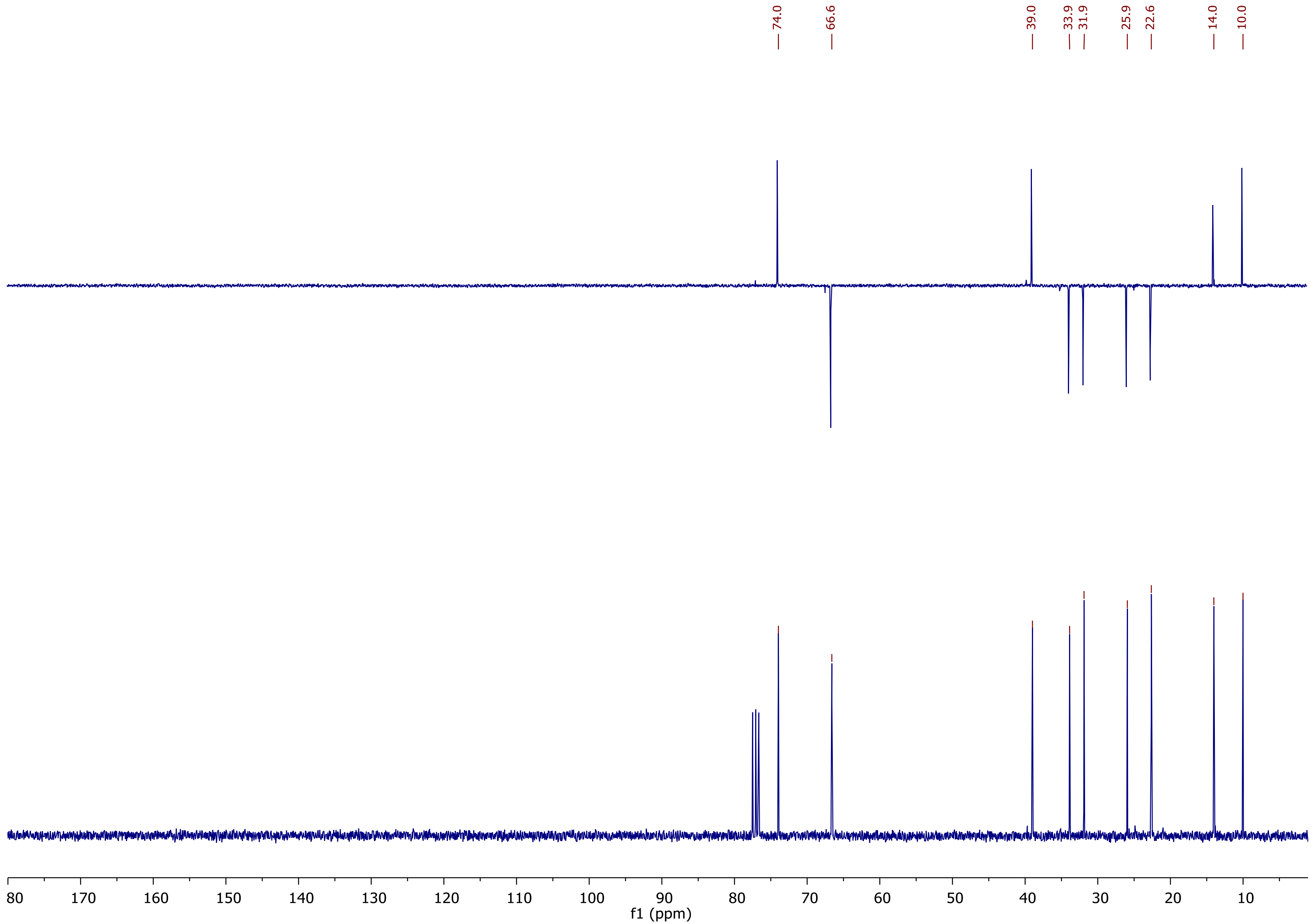

74.0
66.6
39.0
33.9
31.9
25.9
22.6
14.0
10.0
80
170
160
150
140
130
120
110
100
90
80
70
60
50
40
30
20
10
f1 (ppm)

Figure S62

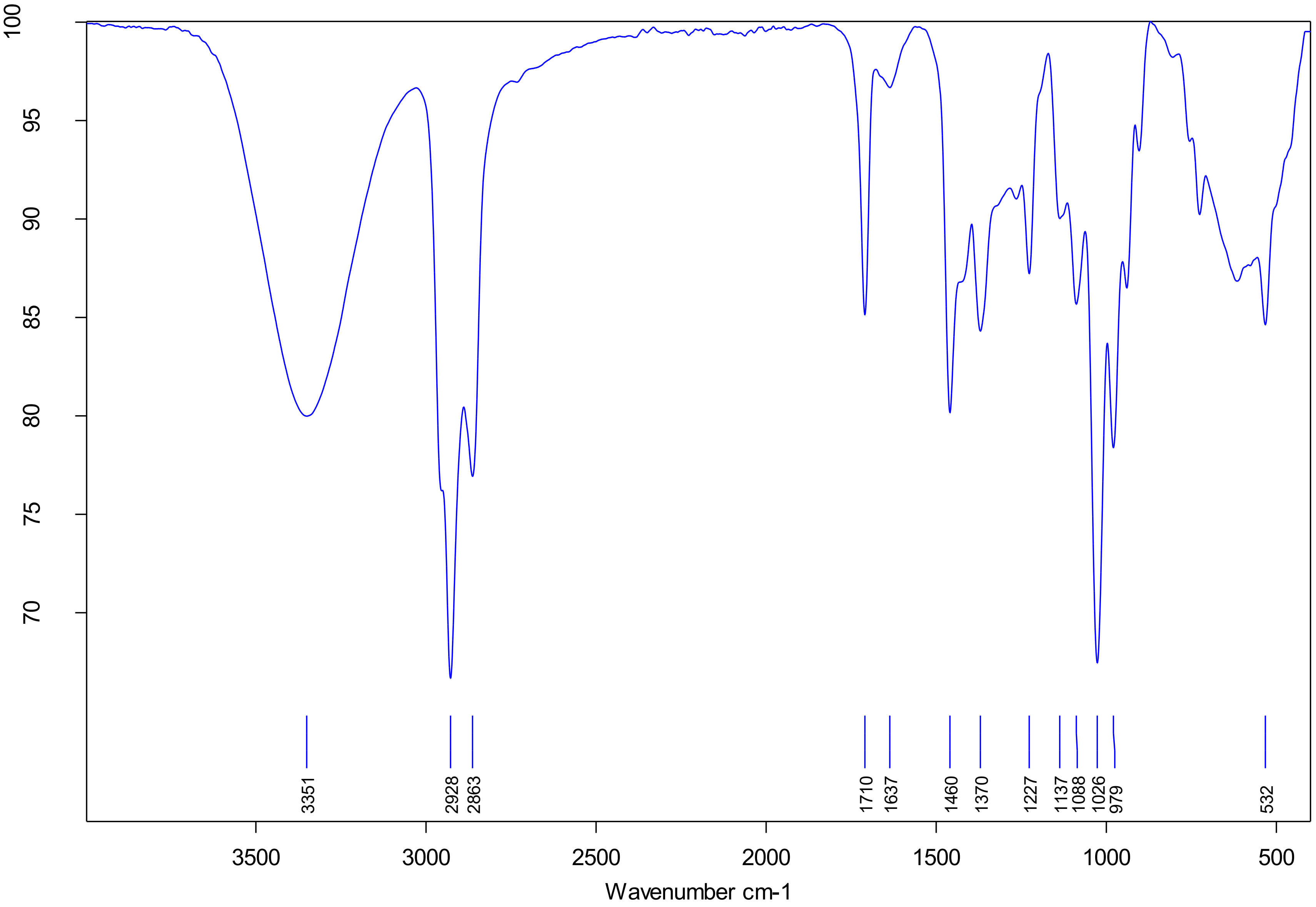

100
95
90
85
80
75
70
3351
2928
2863
1710
1637
1460
1370
1227
1137
1088
1026
979
532
3500
3000
2500
2000
1500
1000
500
Wavenumber cm-1

Figure S63

^{1}H NMR, DEPT 135, ^{13}C NMR, HSQC, NOESY 2D, NOESY 1D and IR of 7

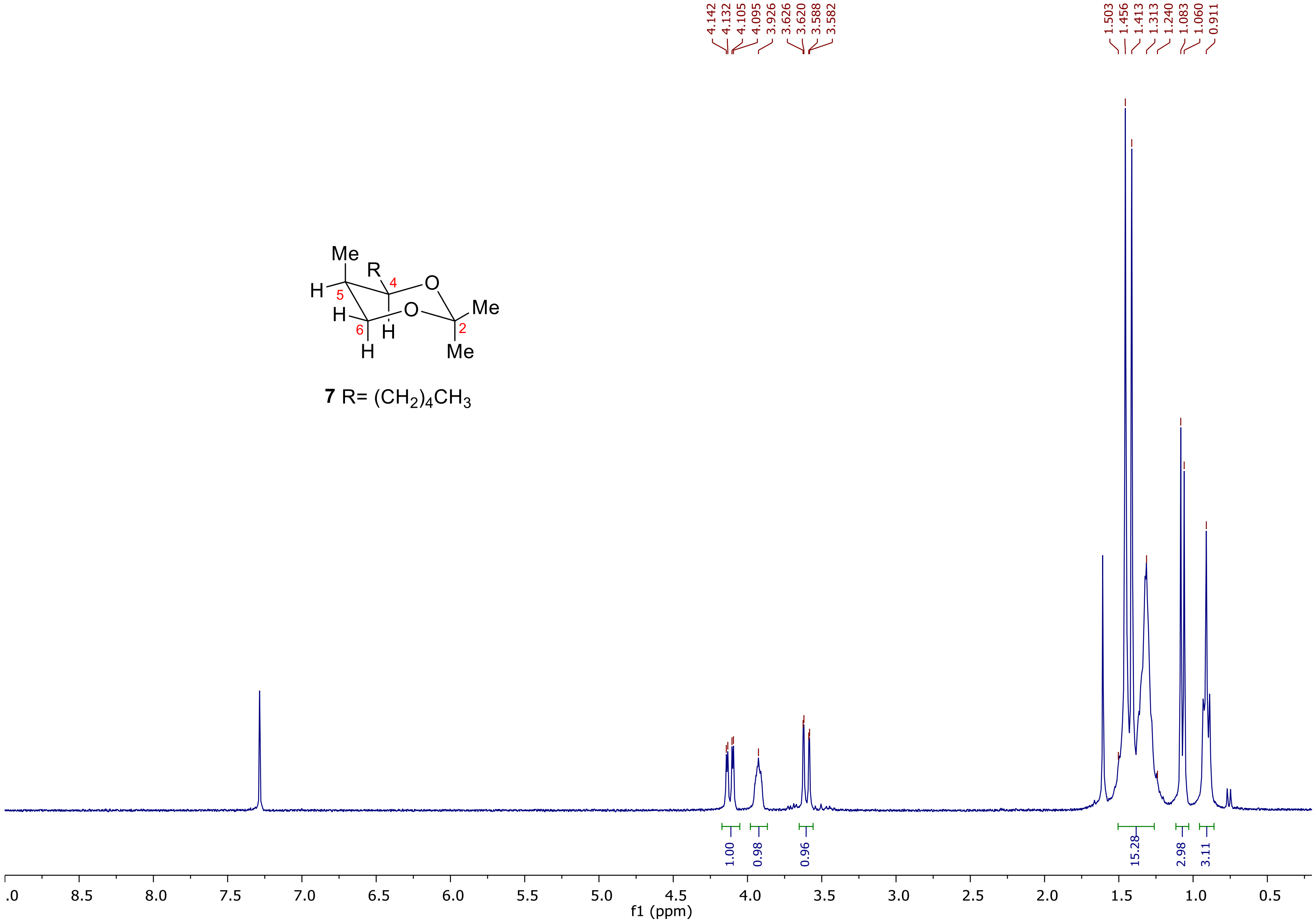

7 R= $(CH_2)_4CH_3$

Figure S64

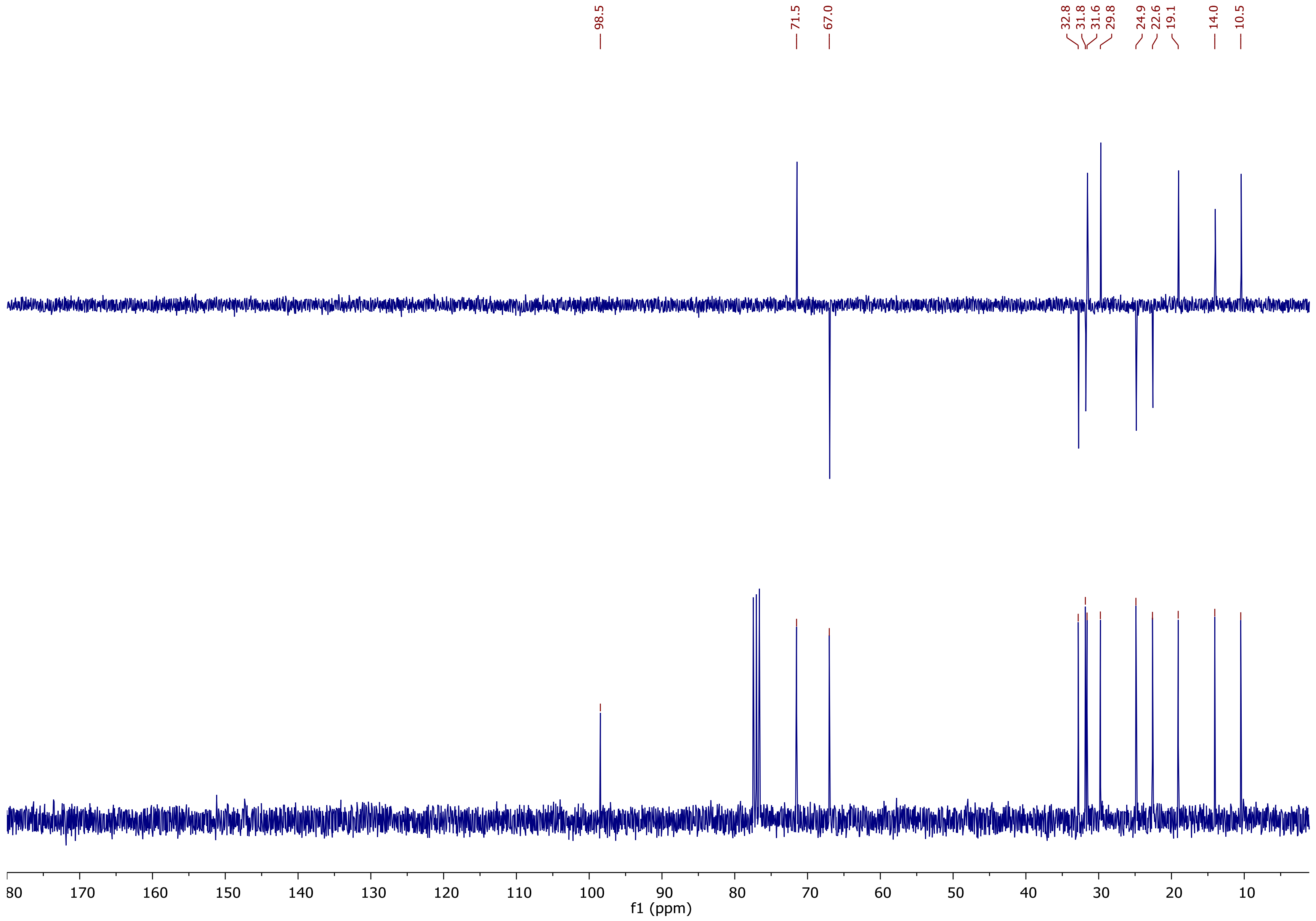

98.5
71.5
67.0
32.8
31.8
31.6
29.8
24.9
22.6
19.1
14.0
10.5
80
170
160
150
140
130
120
110
100
90
80
70
60
50
40
30
20
10
f1 (ppm)

Figure S65

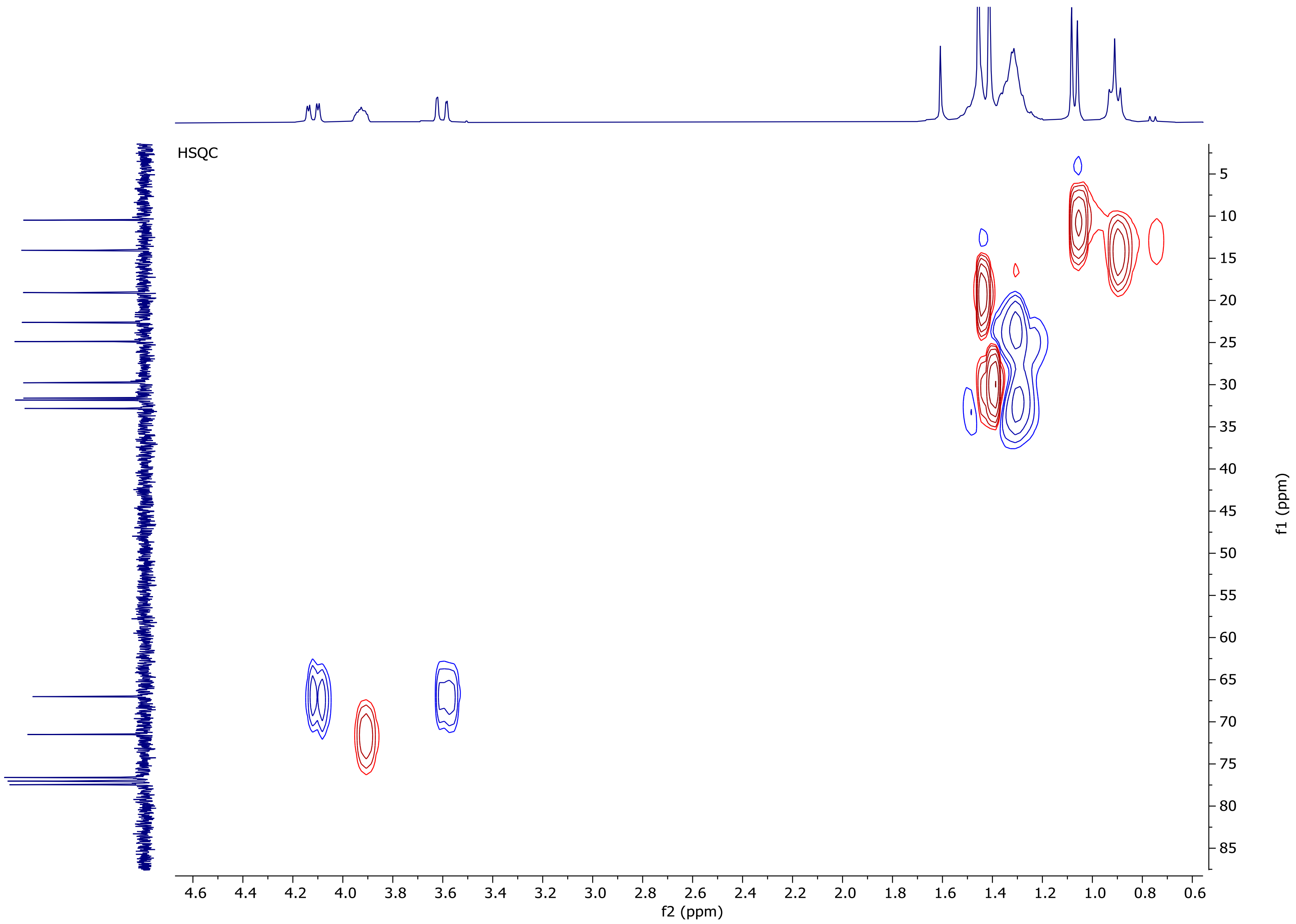
HSQC
f2 (ppm)
f1 (ppm)
4.6
4.4
4.2
4.0
3.8
3.6
3.4
3.2
3.0
2.8
2.6
2.4
2.2
2.0
1.8
1.6
1.4
1.2
1.0
0.8
0.6
5
10
15
20
25
30
35
40
45
50
55
60
65
70
75
80
85

Figure S66

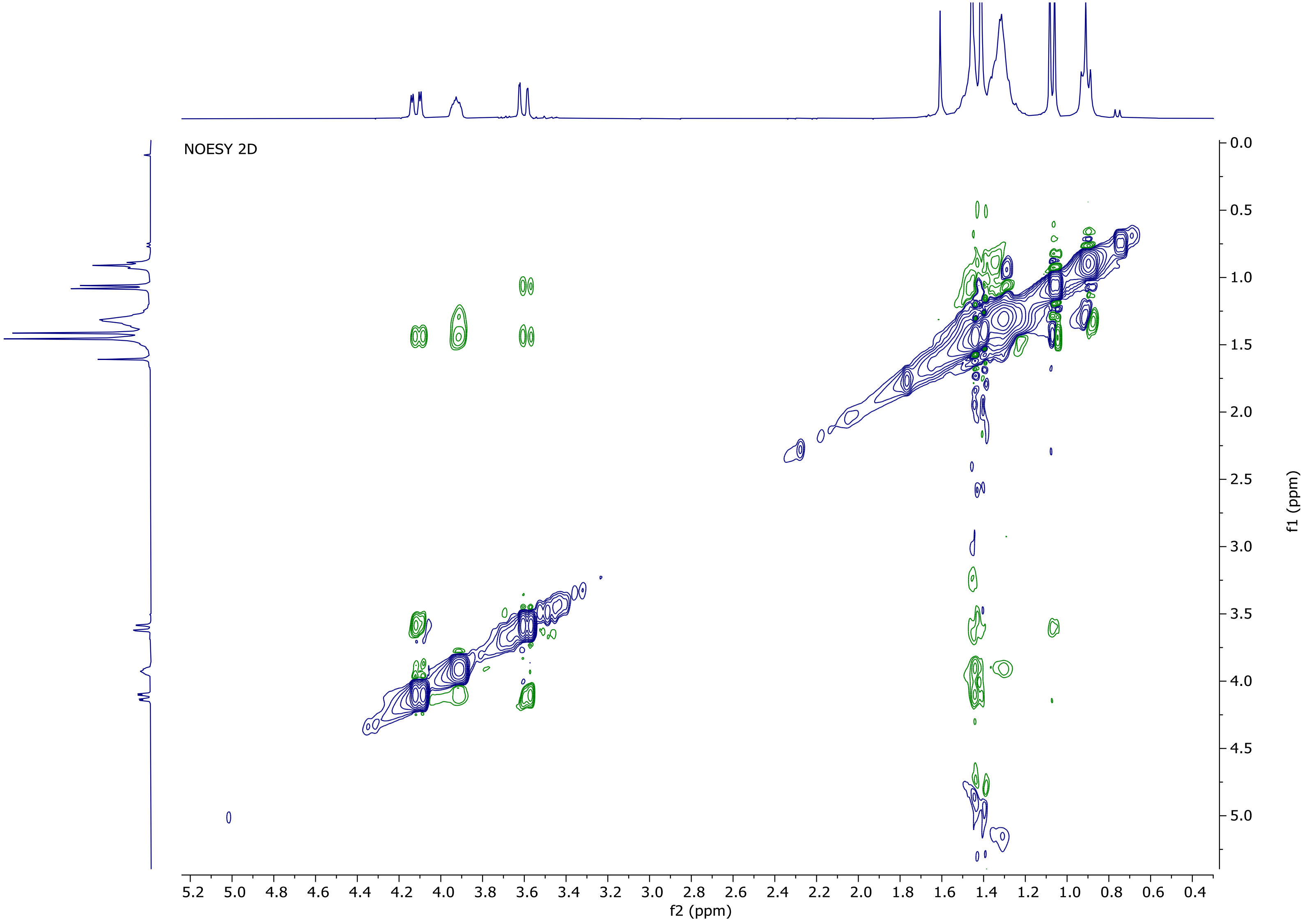
NOESY 2D
f2 (ppm)
f1 (ppm)
5.2 5.0 4.8 4.6 4.4 4.2 4.0 3.8 3.6 3.4 3.2 3.0 2.8 2.6 2.4 2.2 2.0 1.8 1.6 1.4 1.2 1.0 0.8 0.6 0.4
0.0 0.5 1.0 1.5 2.0 2.5 3.0 3.5 4.0 4.5 5.0

Figure S67

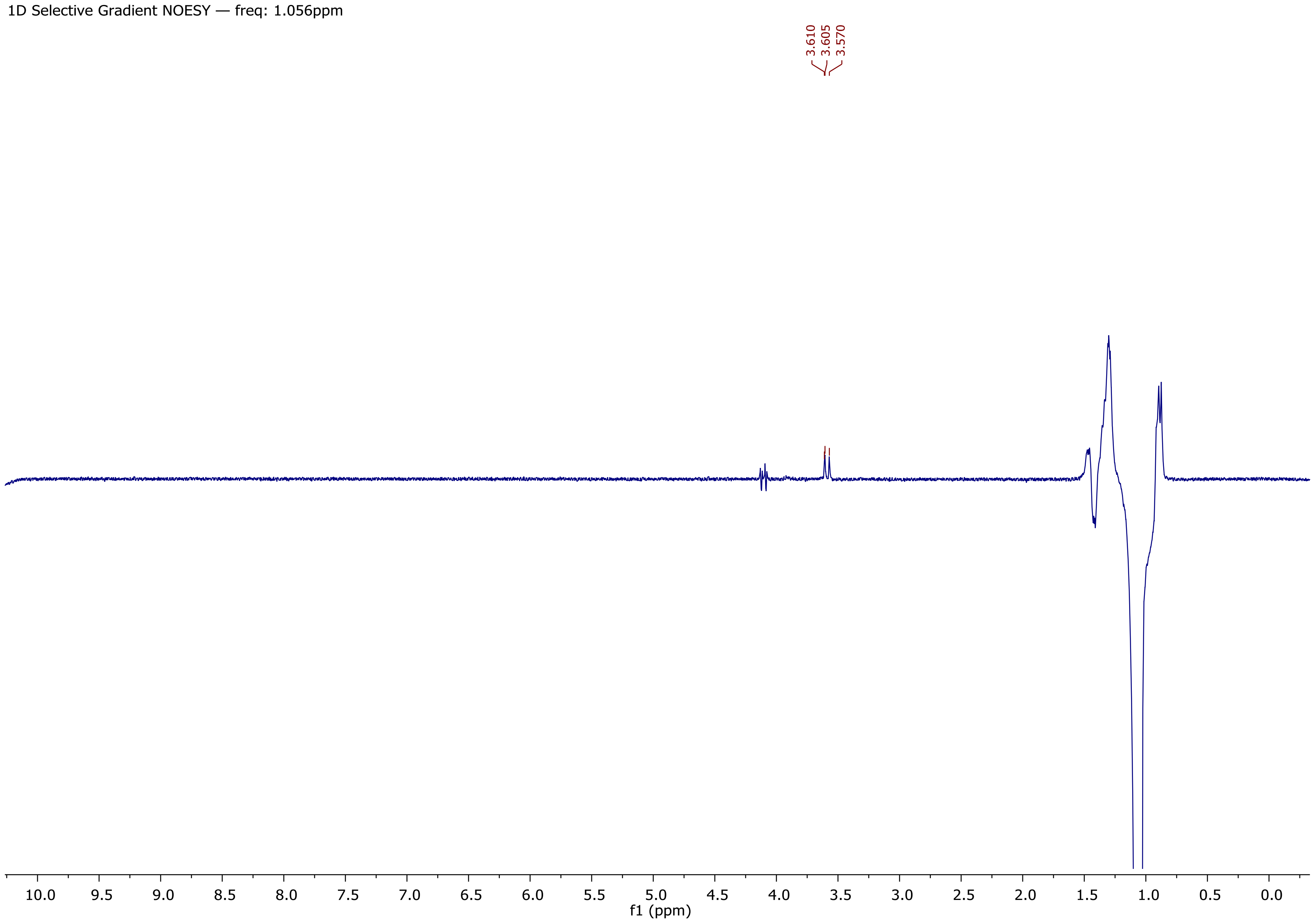
1D Selective Gradient NOESY — freq: 1.056ppm
3.610
3.605
3.570
10.0
9.5
9.0
8.5
8.0
7.5
7.0
6.5
6.0
5.5
5.0
4.5
4.0
3.5
3.0
2.5
2.0
1.5
1.0
0.5
0.0
f1 (ppm)

Figure S68

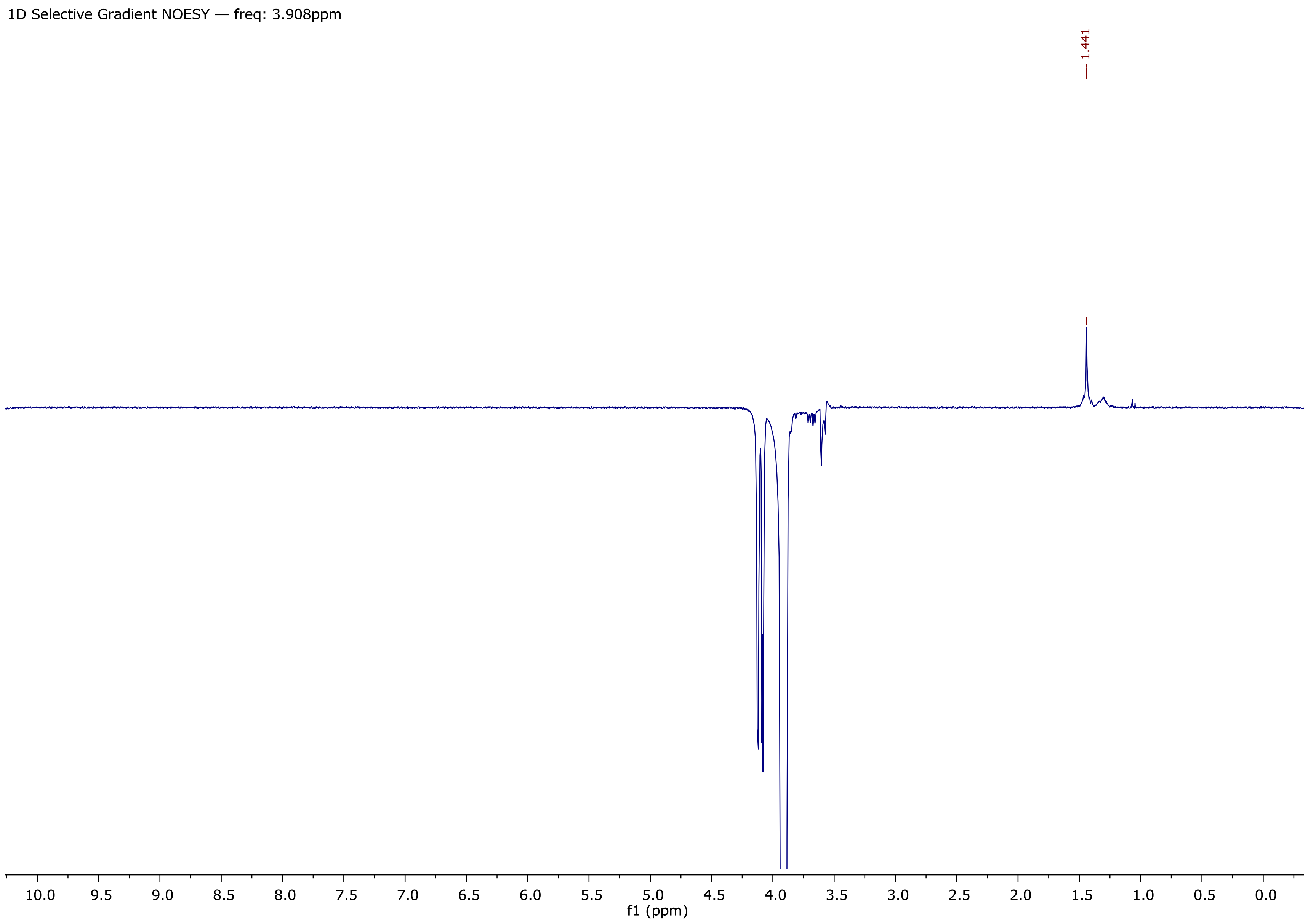
1D Selective Gradient NOESY — freq: 3.908ppm
1.441
10.0
9.5
9.0
8.5
8.0
7.5
7.0
6.5
6.0
5.5
5.0
4.5
4.0
3.5
3.0
2.5
2.0
1.5
1.0
0.5
0.0
f1 (ppm)

Figure S69

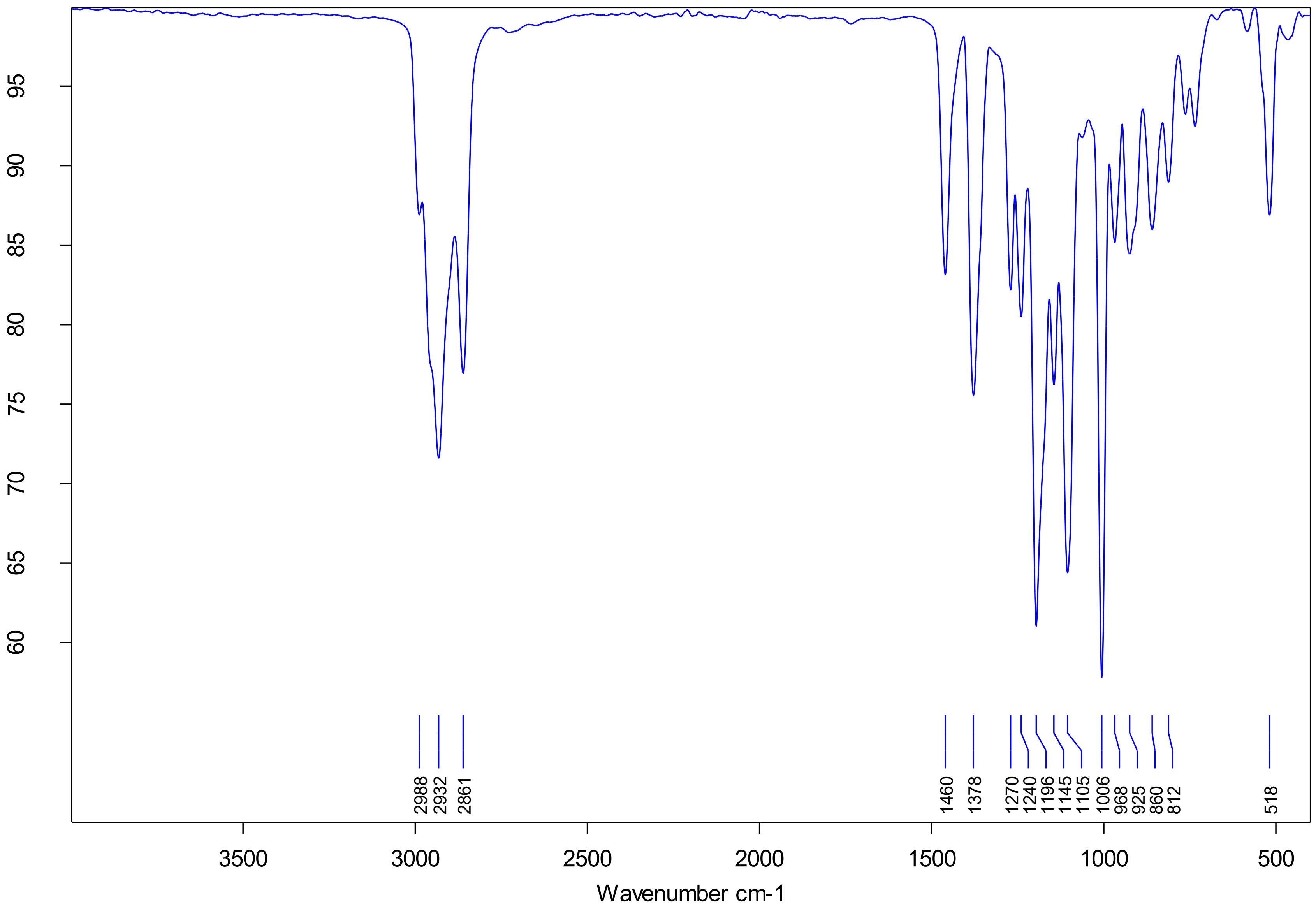
95
90
85
80
75
70
65
60
2988
2932
2861
1460
1378
1270
1240
1196
1145
1105
1006
968
925
860
812
518
3500
3000
2500
2000
1500
1000
500
Wavenumber cm-1

Figure S70
